# Rewiring Oncogenic Transcriptional Complexes with Domain-ALTeration Chimeras (DALTACs) in Prostate Cancer

**DOI:** 10.64898/2026.04.24.720638

**Published:** 2026-04-28

**Authors:** Jie Luo, Jianzhang Yang, Jean Ching-Yi Tien, Mi Wang, Sumit Das, Weiguo Xiang, Eleanor Young, Jelena Tosovic, Rahul Mannan, Jocelyn Cai, Yihan Liu, Kenneth Gu, Somnath Mahapatra, Shiting Li, Yitong Yin, Sanjana Eyunni, Abigail J. Todd, Shicheng Jin, Xuhong Cao, Stephanie J. Miner, Ranga Sudharshan, Arvind Rao, Abhijit Parolia, Yuanyuan Qiao, Shaomeng Wang, Arul M. Chinnaiyan

**Affiliations:** 1. Michigan Center for Translational Pathology, University of Michigan, Ann Arbor, MI, USA.; 2. Department of Pathology, University of Michigan, Ann Arbor, MI, USA.; 3. Division of Hematology-Oncology, Department of Internal Medicine, University of Michigan, Ann Arbor, MI, USA.; 4. Rogel Cancer Center, University of Michigan, Ann Arbor, MI, USA.; 5. Cancer Biology Program, University of Michigan, Ann Arbor, MI, USA.; 6. Howard Hughes Medical Institute, University of Michigan, Ann Arbor, MI, USA.; 7. Department of Computational Medicine and Bioinformatics, University of Michigan, Ann Arbor, MI, USA.; 8. Department of Urology, University of Michigan, Ann Arbor, MI, USA.; 9. Department of Pharmacology, University of Michigan, Ann Arbor, MI, USA; 10. Department of Medicinal Chemistry, University of Michigan, Ann Arbor, MI, USA.; 11. These authors contributed equally.

**Keywords:** androgen receptor, p300/CBP, DALTAC, chemical-induced proximity, neo-enhanceosome, prostate cancer

## Abstract

Transcriptional addiction to the androgen receptor (AR) underlies metastatic castration-resistant prostate cancer (mCRPC), where AR maintains oncogenic enhancer programs through dynamic, domain-specific interactions with the lysine acetyltransferases p300/CBP and associated cofactors. Here, we describe a mechanistically distinct therapeutic modality, Domain-ALTeration Chimeras (DALTACs), designed to rewire endogenous protein complexes by enforcing non-native domain–domain interactions rather than degrading or inhibiting individual components. Our first-in-class molecule, AR–p300/CBP DALTAC-1, induces a synthetic proximity between the AR ligand-binding domain and the p300/CBP bromodomain, thereby misconfiguring the native AR–p300/CBP interface and locking the complex into a non-productive, transcriptionally inert state. DALTAC-1 triggers a profound “super-inhibitory” effect, suppressing AR-driven transcription and proliferation more potently than combined AR and p300/CBP inhibition. Mechanistically, DALTAC-1 reprograms the substrate specificity of p300/CBP, extinguishing the enhancer-associated histone mark H2B N-terminal acetylation (H2BNTac) while inducing neomorphic acetylation of AR and SRC2/3, culminating in collapse of the AR neo-enhanceosome. Chromatin profiling revealed widespread redistribution of AR and p300 toward canonical palindromic AREs, coupled with attenuation of ERG/BRD4 recruitment and a near complete loss of histone H2BNTac acetylation and RNA polymerase II loading at oncogenic AR/ERG neo-enhancers. Strikingly, DALTAC-1 exhibits exquisite lineage selectivity, displaying potent activity in AR-positive prostate cancer cells and patient-derived organoids while sparing AR-negative or non-prostate lineages. In multiple in vivo models, including castration-resistant and patient-derived xenograft tumors, DALTAC-1 induces deep and durable tumor regressions with favorable tolerability. Together, these findings establish DALTACs as a broadly applicable strategy to rewire disease-defining protein complexes by altering their domain topology, expanding the conceptual and therapeutic landscape of induced proximity agents. The precision and lineage-selective action of DALTAC-1 highlight its strong translational potential for treating AR-driven prostate cancer.

## Introduction

Aberrant transcriptional programs are a hallmark of cancer, driving malignant transformation, particularly in ehancer-driven cancers such as metastatic castration-resistant prostate cancer (mCRPC)^1,2^. The central driver of mCRPC is the androgen receptor (AR), which is activated upon androgen binding and subsequently dimerizes and loads onto enhancer regions enriched with androgen response elements (AREs)^3^. Upon chromatin engagement, AR recruits epigenetic coregulators to assemble cancer-specific neo-enhanceosome complexes^4^. These complexes establish highly active chromatin environments that promote oncogenic gene expression programs through enhancer–promoter looping interactions which recruit the transcriptional machinery^2,5–9^.

Enhancer activation requires specific histone modifications^10^. Among the key coactivators in mCRPC that sustain enhancer activity are the paralogous lysine acetyltransferases p300 and CBP^11,12^. These enzymes act as essential transcriptional coactivators by acetylating histone and non-histone proteins, facilitating the recruitment of transcriptional machinery, and serving as scaffolds for enhancer-associated protein assemblies^13–15^. Our previous study revealed that p300/CBP and histone H2B N-terminal multisite acetylation (H2BNTac), a modification exclusively dependent on p300/CBP and a hallmark of active intergenic enhancers^16^, are consistently and significantly elevated in prostate tumor samples^17^. Furthermore, AR and p300 co-bound enhancers are hyperactivated, highlighting the critical role of p300 in driving the oncogenic AR transcriptional program in prostate cancer^17^. Although inhibitors and degraders have been developed to therapeutically target these critical enzymes in enhancer-driven malignancies, including CRPC^17–19^, global inhibition of p300/CBP raises the concern of potential side effects in normal tissues^20^, emphasizing the need for tissue-selective targeting strategies.

Protein–protein interactions (PPIs) are tightly regulated to ensure the functional activation of protein partners, with domain-level compatibility serving as a key determinant of specificity and efficacy^21,22^. In transcriptional regulation, the formation of multi-component enhancer complexes relies on precise domain–domain interactions that orchestrate cofactor recruitment, enzymatic activation, and chromatin engagement^23,24^. For instance, upon chromatin binding and dimerization, the N-terminal domain (NTD) of AR interacts with p300^25^. This interaction stimulates the histone acetyltransferase (HAT) activity of p300, leading to enhancer remodeling and transcriptional activation through the establishment of a permissive chromatin landscape^26,27^. Disrupting the precise AR-p300/CBP protein interaction therefore represents an intriguing strategy to selectively target p300/CBP in AR-positive prostate cancer cells.

Chemical-induced proximity (CIP) strategies have revolutionized therapeutic design by reprogramming protein–protein interactions to manipulate cellular function^28^. Traditional CIP modalities include proteolysis-targeting chimeras (PROTACs), which promote target protein degradation via E3 ligase recruitment^29^, and more recent approaches such as regulated induced proximity targeting chimeras (RIPTACs) and transcriptional/epigenetic chemical inducers of proximity (TCIPs). RIPTACs are heterobifunctional molecules that induce stable interactions between a target protein and a pan-essential effector, resulting in effector inactivation and selective cell death^30,31^. TCIPs, by contrast, redirect transcriptional coactivators, such as BRD4, CDK9, CDK12, or p300, to activate pro-apoptotic genes normally repressed by BCL6^32-34^. Another emerging class, termed targeted relocalization-activating molecules (TRAMs), induces subcellular relocalization of endogenous proteins, effectively displacing them from their native compartments and disrupting their physiological functions^35^. Despite their mechanistic differences, current CIP approaches predominantly function by inducing proximity of non-native interacting proteins.

Here, we introduce a mechanistically distinct modality, Domain-ALTeration Chimeras (DALTACs), that selectively miswire endogenous protein complexes by inducing non-functional domain–domain interactions between physiological partners. In contrast to previously reported CIP strategies that induce proteolysis or repurpose coactivators, DALTACs function as chemical dominant-negatives, locking transcriptional regulators into inert conformations that disable complex activity. As a proof of concept, we developed the first-in-field AR–p300/CBP DALTAC compound, named DALTAC-1 (JYZ3032). DALTAC-1 exhibits superior inhibitory efficacy in AR-positive prostate cancer cells compared to combination treatment with an AR inhibitor and p300/CBP bromodomain (BRD) inhibitor. Importantly, DALTAC-1 limits its action to AR-positive, p300-dependent cancer cells, highlighting its striking lineage-selective tumor inhibitory properties. Mechanistic analyses revealed that DALTAC-1 rewires the native AR-NTD and p300/CBP complex by enforcing neomorphic interactions between the AR ligand-binding domain (LBD) and the p300/CBP BRD, leading to reprogramming of the p300 substrate spectrum. Intriguingly, in contrast to p300/CBP BRD inhibitors, DALTAC-1 rapidly eliminates H2BNTac, the active enhancer mark which is independent of p300/CBP BRD activity^17^, and disrupts the oncogenic program driven by AR and p300/CBP. Profiling of the chromatin landscape revealed a striking enhancement of AR-p300 co-occupancy at AR palindromic binding sites in DALTAC-1 treated cells, yet this is accompanied by disruption of the active AR neo-enhanceosome complex. In multiple preclinical models, DALTAC-1 significantly inhibits tumor growth or induces tumor regression without causing overt toxicity. These findings establish DALTACs as a novel therapeutic strategy to selectively inhibit cancer cell growth by miswiring native oncogenic protein complex.

## Results

### Development of the AR-p300/CBP DALTAC to rewire AR-p300/CBP interactions

**Figure 1.**
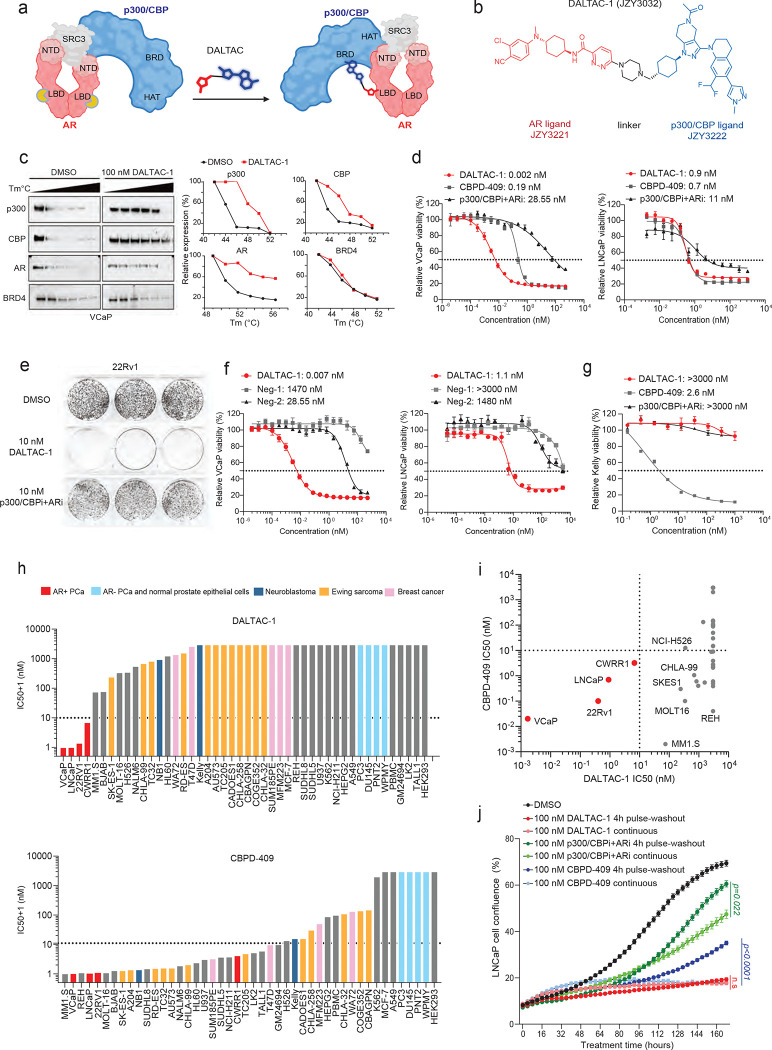
AR-p300/CBP DALTAC-1 selectively suppresses the growth of AR-positive prostate cancer cells. a. Schematic showing AR-p300/CBP DALTAC-1 rewires the domain-domain interactions of the native AR/p300/CBP complex. AR, androgen receptor; NTD, N-terminal domain; CTD, C-terminal domain; BRD, bromodomain; HAT, histone acetyltransferase. b. Schematic illustrating the structure of AR-p300/CBP DALTAC-1 (JZY3032). c. Left: Immunoblot analysis demonstrating stabilization of p300, CBP, and AR, but not BRD4, by DALTAC-1 in a cellular thermal shift assay in VCaP cells. Right: Graphs of relative expression of the indicated proteins in vehicle (dimethyl sulfoxide, DMSO) or DALTAC-1 treated VCaP cells. d. Dose-response curves and IC50 of AR-positive VCaP and LNCaP cells treated with DALTAC-1, CBPD-409, or p300/CBPi+ARi. n=3 biologically replicated wells. e. Representative images of colonies of DMSO, 10 nM DALTAC-1, or 10 nM p300/CBPi+ARi treated 22Rv1 cells. n=3 biologically replicated wells. f. Dose-response curves and IC50 of AR-positive VCaP and LNCaP cells treated with DALTAC-1, Neg-1 (Negative control-1), or Neg-2 (Negative control-2). n=3 biologically replicated wells. g. Dose-response curves and IC50 of Kelly cells treated with DALTAC-1, CBPD-409, or p300/CBPi+ARi. n=3 biologically replicated wells. h. Rank-order plot of IC50 values for DALTAC-1 and CBPD-409 across different human normal and cancer cell lines following 5 days of treatment. Models of AR-positive prostate cancer, AR-negative prostate cancer, and non-neoplastic prostatic cells, neuroblastoma cell lines, Ewing sarcoma cell lines, and breast cancer cell lines are highlighted in specific colors. i. Scatterplot illustrating IC50 values of individual cell lines treated with DALTAC-1 (x-axis) and CBPD-409 (y-axis). AR-positive prostate cancer cell lines are highlighted in red. j. Growth curves of LNCaP cells treated with 100 nM DALTAC-1, combined p300/CBPi + ARi, or CBPD-409 under washout or continuous treatment conditions, monitored using Incucyte Live-Cell Analysis. Data represent mean ± standard deviation (SD), n=6 biologically replicated wells. Statistical significance was assessed using two-way ANOVA.

Previous studies established that AR physically interacts with the coactivator p300 through its N-terminal domain (NTD)^25^, and that dimerized transcription factors can stimulate p300 histone acetyltransferase activity^26^. To reprogram this native interaction, we proposed that by tethering an AR LBD inhibitor with a p300/CBP BRD inhibitor through an appropriate linker, we can develop heterobifunctional molecules to induce non-native AR/p300 interactions and effectively inhibit cell growth of AR-positive cancer cells by concurrently inhibiting both AR and p300 functions (Fig. 1a).

**Extended Data Figure 1.**
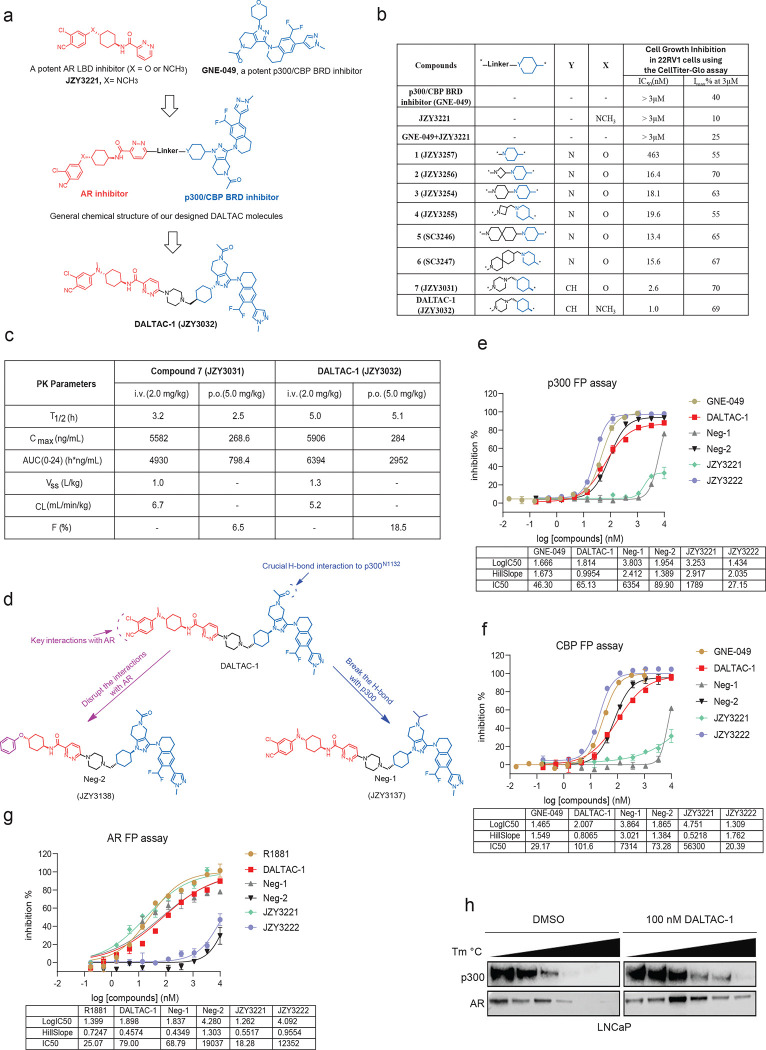
Development of AR-p300/CBP DALTAC-1. a. Chemical structure of the AR ligand-binding domain inhibitor JZY3221 (ARi) and p300/CBP bromodomain inhibitor GNE-049 (p300/CBPi) and the workflow of AR-p300/CBP DALTAC compound design. b. Structure–activity relationship (SAR) table summarizing linker structures, IC50 values, and Imax in 22Rv1 cells for all tested compounds. c. Key PK parameters were measured in mice after intravenous (2.0 mg/kg) or oral (5.0 mg/kg) dosing. The table reports T_1_/_2_, C , AUC_0–24_, Vss, CL, and oral bioavailability (F). d. Modifications of JZY3032 (DALTAC-1) generated the inactive AR–p300 DALTAC analogs JZY3137 (Neg-1, impaired p300/CBP binding, retained AR binding) and JZY3138 (Neg-2, impaired AR binding, retained p300/CBP binding). e. Dose–response curves from FP assays measuring p300 binding for Neg-1, Neg-2, GNE-049 (p300 bromodomain inhibitor), JZY3221, JZY3222, and DALTAC-1. n=2 biologically replicated samples. f. Dose–response curves from FP assays measuring CBP binding for Neg-1, Neg-2, GNE-049 (p300 bromodomain inhibitor), JZY3221, JZY3222, and DALTAC-1. n=2 biologically replicated samples g. Dose–response curves from FP assays measuring AR binding for Neg-1, Neg-2, R1881, JZY3221, JZY3222, and DALTAC-1. n=2 biologically replicated samples. h. Immunoblot analysis demonstrating stabilization of p300 and AR by DALTAC-1 in a cellular thermal shift assay in LNCaP cells.

To test this premise, we designed and synthesized a series of heterobifunctional molecules using a potent p300/CBP BRD inhibitor (GNE-049^19^) and a potent AR inhibitor^36^ tethered through a linker (Extended Data Fig. 1a). We selected the tethering site in the p300/CBP BRD inhibitor based upon the co-crystal structure of GNE-049 and its analogues, which showed that our selected tethering site was exposed to the solvent environment^37^. Similarly, we chose the tethering site in the AR inhibitor based upon our modeled complex structure with AR and extensive structure-activity relationships reported for this class of AR inhibitors^36,38^. We evaluated our synthesized compounds with proliferation assays in AR-positive 22Rv1 cells, which were shown to be responsive to p300/CBP BRD inhibitors but minimally responsive to traditional AR inhibitors^18,39^. GNE-049, the AR inhibitor (JZY3221), and their combination were included as controls. Consistently, the individual treatment of GNE-049 and JZY3221 or the combination of the two compounds displayed limited inhibitory activity (Extended Data Fig. 1b).

To facilitate the synthesis of the designed heterobifunctional compounds, we replaced the tetrahydropyran group in GNE-049 with a piperidinyl group, which was shown to not affect the binding to p300^37^. Directly connecting the AR inhibitor through its pyridazine to the nitrogen atom of the piperidinyl group in the p300/CBP inhibitor yielded heterobifunctional compound **1**, which showed a reasonably potent cell growth inhibition activity with IC50 = 463 nM and Imax = 55% (Extended Data Fig. 1b). Inserting a short and rigid 4-membered azetidine between the pyridazine of the AR inhibitor and the nitrogen atom of the piperidinyl group in the p300/CBP inhibitor as the linker in compound **1** generated compound **2**, which had an IC50 value of 16.4 nM and Imax = 70% (Extended Data Fig. 1b). Replacing the 4-membered azetidine linker in compound **2** with a 6-membered piperidine linker generated compound **3**, which was equally potent and effective compared to compound **2** (Extended Data Fig. 1b). Extending the linker by one methylene group in compound **2** led to compound **4**, which was equally potent (IC50 = 19.6 nM) but less effective (Imax = 55%) as compared to compound **2** (Extended Data Fig. 1b). Changing the 4-membered azetidine in compound **2** with a [6,6] spiro ring system resulted in compound **5**, which was also similarly potent and effective as compared to compound **2** (Extended Data Fig. 1b). Inserting a methylene between the [6,6] spiro ring and the piperidinyl group in compound **5** generated compound **6**, which was similarly potent and effective as compared to compound **5** (Extended Data Fig. 1b). We next synthesized compound **7** using a methyl-piperazinyl group as the linker but changing the piperidinyl group in the p300/CBP inhibitor with a trans-cyclohexyl group for chemical stability considerations. Compound **7** (JZY3031) achieved IC50 = 2.6 nM and Imax = 70%, and was thus >1,000-times more potent than the combination of the AR inhibitor and GNE-049 (Extended Data Fig. 1b).

Pharmacokinetic (PK) data in mice showed that compound **7** had a slow clearance, a moderate volume of distribution, and an excellent exposure with intravenous administration. Surprisingly, despite its high molecular weight (MW = 962), compound **7** achieved appreciable oral exposure with C_max_ = 269 ng/ml and AUC_0-t_ = 798 h* ng/ml at 5.0 mg/kg PO dose and an oral bioavailability of 6.5% (Extended Data Fig. 1c). Our previous study showed that replacement of the oxygen atom with a *N*-methyl group in the AR inhibitor significantly improved oral bioavailability^38^. Accordingly, we replaced the oxygen atom with a *N*-methyl group in the AR inhibitor portion in compound **7**, which yielded JZY3032 (Fig. 1b). JZY3032 attained an IC50 value of 1.0 nM and Imax = 69% and was therefore more potent than compound **7** (Extended Data Fig. 1b). A PK study revealed that JZY3032 had an improved PK profile with intravenous administration over compound **7**, characterized by a slower clearance, a higher volume of distribution, and a higher exposure. Even more significantly, JZY3032 had an improved oral PK profile over compound **7,** achieving a good oral exposure (C_max_ = 284 ng/ml and AUC_0-t_ = 2952 h* ng/ml at 5.0 mg/kg dose) and an oral bioavailability of 18.5% (Extended Data Fig. 1c). Based upon our extensive mechanistic data obtained, we named JZY3032 as DALTAC-1 (Domain-ALTeration Chimera 1) and employed it for our subsequent in vitro and in vivo investigations. To verify that the activity of DALTAC-1 was dependent on its binding to both AR and p300, two negative control compounds, Negative control-1 (Neg-1) and Negative control-2 (Neg-2), were designed by disrupting the critical H-bond with p300 N1132^37^ and AR R752^40^, respectively (Extended Data Fig. 1d). Fluorescence polarization (FP) assays confirmed that DALTAC-1 bound both p300 and AR with comparable affinities to their respective parent inhibitors, whereas Neg-1 and Neg-2 exhibited markedly reduced affinities toward p300 or AR (Extended Data Fig. 1e-g).

A cellular thermal shift assays (CESTA) revealed a pronounced increase in the thermal stability of AR, p300, and its analog protein CBP, but not BRD4, upon DALTAC-1 treatment, supporting strong and specific engagement of DALTAC-1 with its intended targets (Fig. 1c and Extended Data Fig. 1h). Together, these data demonstrate that by tethering an AR LBD inhibitor and a p300/CBP BRD domain inhibitor with appropriate linkers, we have identified a series of heterobifunctional molecules, which achieved improved cell growth inhibitory activity than the combination of the parent AR inhibitor and the corresponding p300/CBP BRD inhibitor. In particular, DALTAC-1 is >3,000 times more potent than the combination of the AR inhibitor and the p300/CBP BRD inhibitor in cell growth inhibition in the 22Rv1 cell line and is more effective (Imax = 69% vs 25%). Despite its high MW of 975, DALTAC-1 achieves a good oral bioavailability and an excellent PK profile with intravenous administration.

### AR-p300/CBP DALTAC-1 selectively suppresses AR-positive prostate cancer cell growth

**Extended Data Figure 2.**
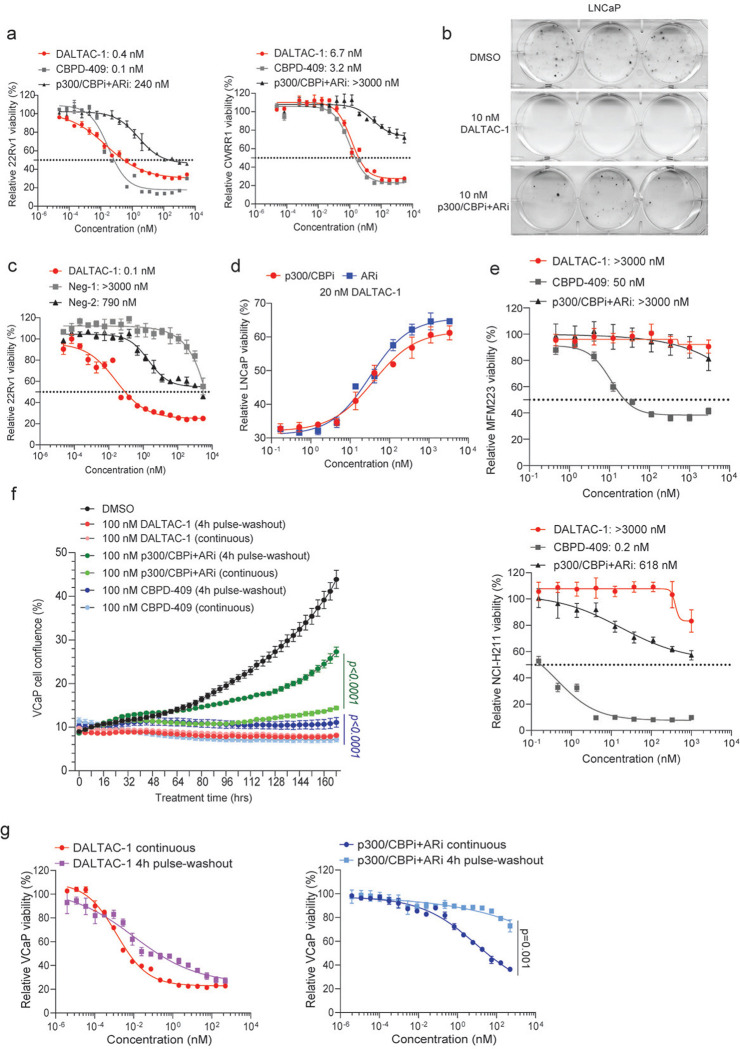
AR-p300/CBP DALTAC-1 specifically inhibits AR-positive prostate cancer growth. a. Dose-response curves and IC50 of AR-positive 22Rv1 and CWRR1 cells treated with DALTAC-1, CBPD-409, or p300/CBPi+ARi. n=3 biologically replicated wells. b. Representative images of colonies of DMSO, 10 nM DALTAC-1, or 10 nM p300/CBPi+ARi treated LNCaP cells. n=3 biologically replicated wells. c. Dose-response curves and IC50 of AR-positive 22Rv1 cells treated with DALTAC-1, Neg-1, or Neg-2. n=3 biologically replicated wells d. Dose-response curves of LNCaP cells treated with 20 nM DALTAC-1 and increasing concentrations of p300/CBPi or ARi. n=6 biologically replicated wells. e. Dose-response curves and IC50 of MFM223 and NCI-H211 cells treated with DALTAC-1, CBPD-409, or p300/CBPi+ARi. n=3 biologically replicated wells. f. Growth curves of VCaP cells treated with 100 nM DALTAC-1, combined ARi + p300/CBPi, or CBPD-409 under washout or continuous treatment conditions, monitored using Incucyte Live-Cell Analysis. Data represent mean ± SD. n=6 biologically replicated wells. Statistical significance was assessed using two-way ANOVA. g. Dose-response curves of LNCaP cells treated with DALTAC-1 or combined ARi + p300/CBPi under washout or continuous treatment conditions. Data represent mean ± SD. n=6 biologically replicated wells. Statistical significance was assessed using two-way ANOVA.

To further assess the activity and selectivity of AR–p300/CBP DALTAC-1, we compared its cell growth inhibitory effects with those of the potent and efficacious p300/CBP dual degrader CBPD-409^41^. We also compared DALTAC-1 to the combination of p300/CBP BRD inhibitor GNE-049 (p300/CBPi) and AR-LBD inhibitor JZY3221 (ARi). Treatment with DALTAC-1 resulted in potent cytotoxicity across all tested AR-positive prostate cancer cell lines (VCaP, LNCaP, 22Rv1, and CWRR1), similar to or better than that observed with CBPD-409 (Fig. 1d and Extended Data Fig. 2a). Notably, DALTAC-1 demonstrated superior activity relative to the combined treatment with AR-LBD and p300/CBP-BRD inhibitors in all tested AR-positive prostate cancer cells, indicating a gain-of-function mechanism arising from enforced miswiring of the AR–p300 domain interactions (Fig. 1d and Extended Data Fig. 2a). Consistently, colony formation assays revealed that DALTAC-1 abolished the clonogenic potential of AR-positive prostate cancer cells, outperforming the combined p300/CBPi and ARi treatment (Fig. 1e and Extended Data Fig. 2b). In contrast, the inactive analogs Neg-1 and Neg-2 exhibited dramatically reduced cytotoxic effects (Fig. 1f and Extended Data Fig. 2c). Furthermore, addition of p300/CBPi or ARi in molar excess competitively blocked DALTAC-1 binding to p300/CBP or AR and significantly attenuated its activity, indicating that engagement of both AR and p300/CBP is essential for the superior activity of DALTAC-1 (Extended Data Fig. 2d).

Compared with CBPD-409, DALTAC-1 exhibited markedly reduced cytotoxicity in AR-negative yet p300/CBP-dependent cancer cells (Kelly, NCI-H211, and MFM223) (Fig. 1g and Extended Data Fig. 2e), highlighting its specificity toward dual AR–p300/CBP dependency. To validate this selectivity, we systematically compared the anti-proliferative effects (IC50 values) of DALTAC-1 and CBPD-409 across a broad panel of cancer cell lines. In contrast to the broad anti-proliferative activity of CBPD-409 across diverse cancer lineages, DALTAC-1 exhibited remarkable lineage selectivity, effectively inhibiting proliferation exclusively in AR-positive prostate cancer cells (Fig. 1h, i). Notably, DALTAC-1 also exhibited prolonged cytotoxic effects. A 4-hour pulse treatment of DALTAC-1 followed by compound washout resulted in comparable inhibition to continuous exposure, whereas washout of CBPD-409 or the p300/CBPi + ARi combination led to a marked reduction in anti-proliferative activity (Fig. 1j and Extended Data Fig. 2f, g). These results highlight the durable and sustaining nature of DALTAC-1 activity. Collectively, these findings establish DALTAC-1 as a potent and selective heterobifunctional compound with strong inhibitory activity in AR-positive prostate cancer cells.

### DALTAC-1 induces AR and p300 proteins into proximity to form a stable ternary complex

**Extended Data Figure 3.**
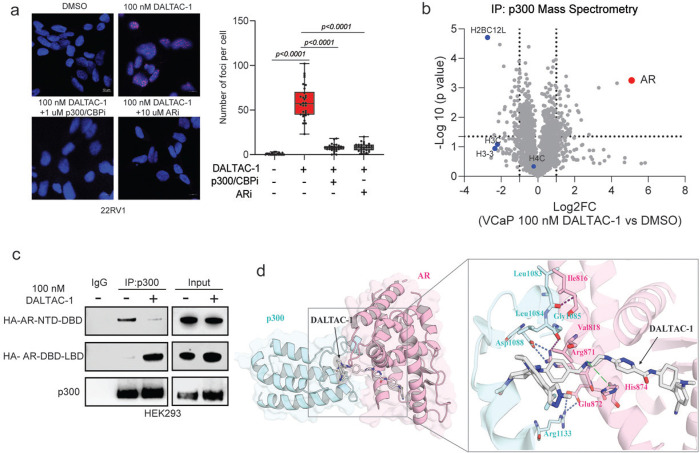
DALTAC-1 induces formation of an AR-p300-DALTAC-1 ternary complex. a. Left: Representative images of AR–p300 PLA foci in 22Rv1 cells treated with DMSO, 100 nM DALTAC-1, 100 nM DALTAC-1 + 1 μM p300/CBPi, or 100 nM DALTAC-1 + 10 μM ARi for 4 hours, scalebar = 10 μm. Right: Quantification of AR–p300 PLA foci per cell (n = 30 cells). P values were calculated using a two-sided t-test. b. Volcano plot showing changes in p300-interacting proteins in VCaP cells treated with 100 nM DALTAC-1 versus DMSO for 4 hours by IP-mass spectrometry. Histone proteins are highlighted in blue and AR in red. Data represent n = 3 independent experiments. P values were calculated using a two-sided t-test. c. Immunoblot analysis of co-IP assay showing the interaction of AR-NTD-DBD or AR-DBD-LBD domains with full-length p300 in HEK293 cells treated with DMSO or 100 nM DALTAC-1 for 4 hours. d. Computational modeling of the AR-LBD/DALTAC-1/p300-BRD ternary complex. DALTAC-1 (gray sticks), AR-LBD (pink, PDB: 2AXA), p300 (cyan, PDB: 5W0E), hydrogen bonds (purple), salt bridges (blue), and π–cation interactions between the piperazine of DALTAC-1 and His874 (green), nitrogen atoms in blue, oxygen atoms in red and F atoms in light blue.

**Figure 2.**
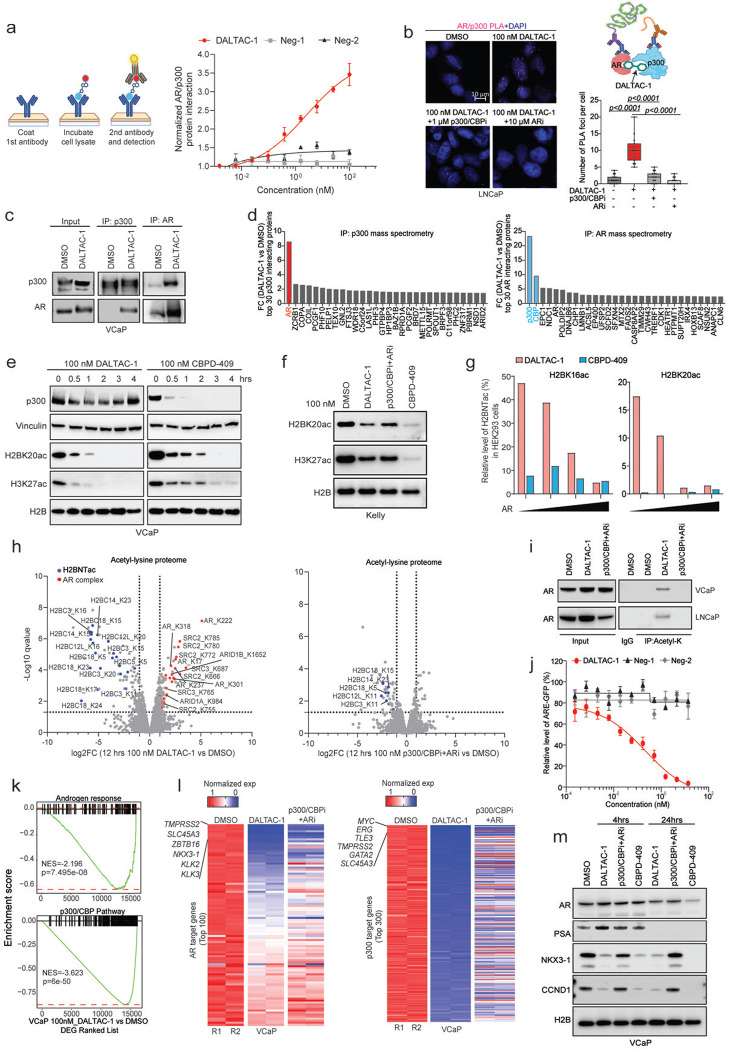
DALTAC-1-induced proximity of AR and p300 proteins inhibits their oncogenic transcriptional output in AR-positive prostate cancer cells. a. Left: Schematic illustrating the workflow of the ternary complex ELISA. Right: Dose–response curves showing DALTAC-1, Neg-1, and Neg-2 induced formation of the AR/p300 ternary complex. Data represent mean ± SD. n=4 biologically replicated wells. b. Left: Representative images of AR–p300 PLA foci in LNCaP cells treated with DMSO, 100 nM DALTAC-1, 100 nM DALTAC-1 + 1 μM p300/CBPi, or 100 nM DALTAC-1 + 10 μM ARi for 4 hours, scalebar = 10 μm. Upper right: Schematic depicting the working model of the PLA assay. Lower right: Quantification of AR–p300 PLA foci per cell (n = 20 cells). P values were calculated using a two-sided t-test. c. Immunoblot analysis of the co-immunoprecipitation (Co-IP) assay showing that 100 nM DALTAC-1 increases the interaction between AR and p300 proteins. d. Bar graphs showing the top 30 proteins whose interactions with p300 (left) or AR (right) are significantly increased in DALTAC-1 treated VCaP cells, as determined by IP–mass spectrometry analysis. AR is highlighted in red, and p300/CBP are highlighted in blue. e. Immunoblot analysis of indicated proteins and histone marks in VCaP cells treated with 100 nM DALTAC-1 or CBPD-409 for noted durations. f. Immunoblot analysis of H2BK20ac and H3K27ac in Kelly cells treated with 100 nM DALTAC-1, combination of p300/CBPi and ARi, or CBPD-409 for 4 hours. g. Bar charts showing the relative expression of the indicated histone marks in HEK293 cells expressing varying levels of ectopic AR and treated with 100 nM DALTAC-1 or CBPD-409 for 4 hours, as determined by immunoblot analysis. h. Volcano plots showing altered lysine acetylation levels in VCaP cells treated with 100 nM DALTAC-1 versus DMSO (left) or a combination of p300/CBPi and ARi (right) versus DMSO for 12 hours. H2BNTac sites are highlighted in blue, and components of the AR complex are highlighted in red. Data plotted from n=3 independent samples. Statistical tests were two-tailed t-tests. i. Immunoblot analysis showing AR acetylation in VCaP and LNCaP cells treated with 100 nM DALTAC-1 or the combination of p300/CBPi and ARi for 4 hours. j. Dose–response curves of GFP fluorescence in LNCaP-ARE-GFP reporter cells treated with DALTAC-1, Neg-1, or Neg-2 for 48 hours. Data represent mean ± SD. n=3 biologically replicated wells. k. GSEA plots for AR and p300/CBP pathway-related genes using the fold change rank-ordered gene signature from the DALTAC-1 treated VCaP cells. NES, net enrichment score; adj P, adjusted p-value; DEGS, differentially expressed genes. n=2 biological replicates. Statistical significance was assessed using a two-sided GSEA permutation test with adjustment for multiple comparisons. l. RNA-seq heatmaps for top 100 AR target genes (left) and top 300 p300/CBP target genes (right) in VCaP cells treated with 100 nM DALTAC-1 or 100 nM combination of p300/CBPi and ARi for 4 hours. n=2 biological replicates. m. Immunoblot analysis of indicated proteins in VCaP cells treated with 100 nM DALTAC-1, combination of p300/CBPi and ARi, or CBPD-409 for the noted durations.

Ternary complex formation between target proteins and a small molecule is a central mechanism underlying CIP strategies^42^. To determine whether DALTAC-1 mediated ternary complex formation, we performed a ternary complex ELISA and observed that DALTAC-1 markedly enhanced AR–p300 interactions in VCaP cells (Fig. 2a). In contrast, the inactive analogs Neg-1 and Neg-2, deficient in binding to p300 or AR, respectively, failed to promote this interaction (Fig. 2a). A proximity ligation assay (PLA) further confirmed that DALTAC-1 treatment substantially increased the number of AR–p300 PLA foci, indicating enhanced spatial proximity between the two proteins (Fig. 2b and Extended Data Fig. 3a). This effect was competitively blocked by either a p300 inhibitor or an AR inhibitor, supporting the formation of a DALTAC-1-induced AR–p300 ternary complex (Fig. 2b and Extended Data Fig. 3a).

Co-immunoprecipitation (Co-IP) assays further demonstrated that DALTAC-1 robustly enhanced AR–p300 interactions in AR-positive VCaP cells (Fig. 2c). To comprehensively characterize the DALTAC-1–induced complex, we performed immunoprecipitation followed by mass spectrometry (IP–MS) in VCaP cells treated with DMSO or DALTAC-1. In DALTAC-1–treated samples, p300 immunoprecipitates were strongly enriched for AR, whereas AR immunoprecipitates showed p300 and its paralog CBP as the top interactors (Fig. 2d and Extended Data Fig. 3b). These reciprocal enrichments provide proteomic evidence that DALTAC-1 promotes the assembly of a stable AR–p300 complex in cells.

To validate the domain-alteration mechanism of DALTAC-1, we ectopically co-expressed AR-NTD–DBD, AR-DBD–LBD, and p300 constructs in HEK293 cells, followed by treatment with DMSO or DALTAC-1. Co-IP analysis revealed that DALTAC-1 inhibited the native interaction between the AR-NTD and p300, while markedly enhancing a neomorphic interaction between the AR-LBD and p300. These results demonstrate that DALTAC-1 redirects p300 engagement from the AR-NTD to the AR-LBD (Extended Data Fig. 3c).

To gain structural insights into its mechanism of action, we conducted a computational modeling of the AR-LBD/DALTAC-1/p300-BRD ternary complex. The top-scoring complex was subjected to a 1 μs molecular dynamics (MD) simulation (Extended Data Fig. 3d). In addition to those interactions observed for ARi and p300/CBPi for their respective proteins, the predicted AR- DALTAC-1-p300 ternary complex revealed a well-defined interface between AR and p300, stabilized by a network of polar and hydrophobic interactions (Extended Data Fig. 3d). A strong salt bridge was formed between Arg871 in AR and Asp1088 in p300, complemented by a hydrogen bond between Arg871 and the backbone carbonyl of Gly1085. A second key contact involved a backbone–backbone hydrogen bond between Ile816 (AR) and Leu1083 (p300), further supported by hydrophobic packing between their side chains. Additional hydrophobic stabilization was provided by interactions between Val818 (AR) and Leu1084 (p300). Notably, a π–cation interaction was detected between His874 (AR) and the piperazine moiety of DALTAC-1. Taken together, our data establish that DALTAC-1 induces a stable AR–p300 ternary complex and rewires their domain-level interactions, providing the mechanistic foundation for its downstream epigenetic and transcriptional effects.

### DALTAC-1 selectively reprograms the acetylome in AR-positive prostate cancer cells

**Extended Data Figure 4.**
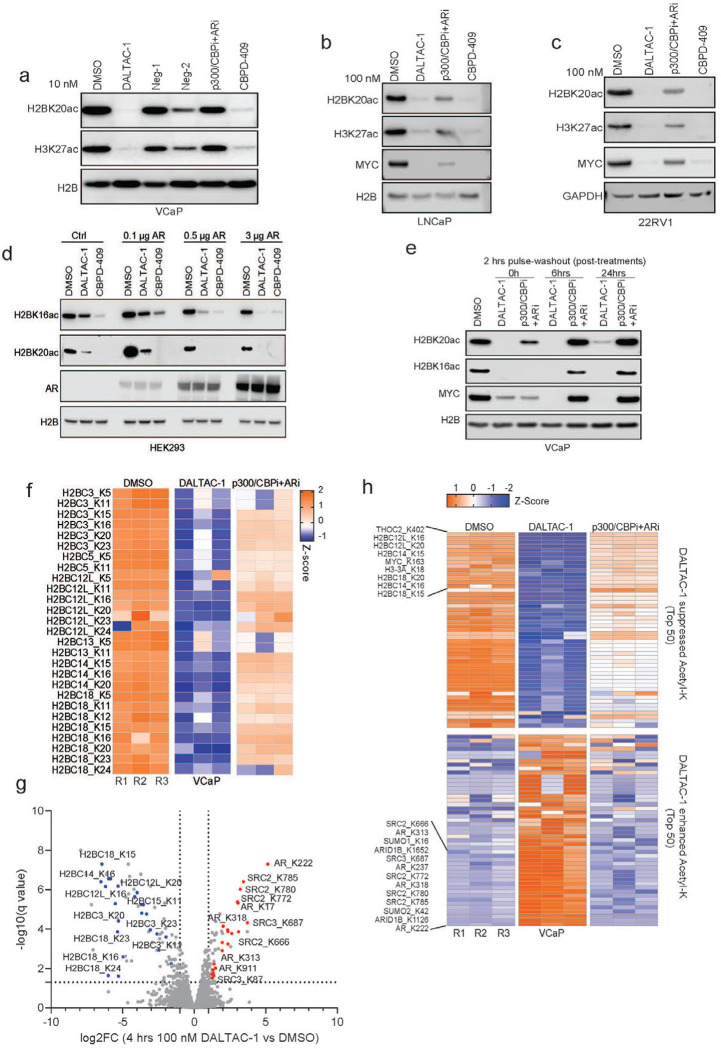
DALTAC-1 reprograms the substrate spectrum of p300/CBP in an AR-dependent manner. a. Immunoblot analysis of indicated histone marks in VCaP cells treated with DMSO, 10 nM DALTAC-1, 10 nM Neg-1, Neg-2, 10 nM combination of p300/CBPi and ARi, or 10 nM CBPD-409 for 4 hours. b. Immunoblot analysis of indicated histone marks and proteins in LNCaP cells treated with DMSO, 100 nM DALTAC-1, 100 nM combination of p300/CBPi and ARi, or 100 nM CBPD-409 for 4 hours. c. Immunoblot analysis of indicated histone marks and proteins in 22Rv1 cells treated with DMSO, 100 nM DALTAC-1, 100 nM combination of p300/CBPi and ARi, or 100 nM CBPD-409 for 4 hours. d. Immunoblot analysis of indicated histone marks and proteins in HEK293 cells expressing varying levels of ectopic AR and treated with 10 nM DALTAC-1 or CBPD-409 for 4 hours. e. Immunoblot analysis of indicated histone marks and proteins in VCaP cells pre-treated with 100 nM DALTAC-1 or a combination of p300/CBPi and ARi for 2 hours, followed by washout and culture for the indicated durations. f. Acetyl-lysine mass spectrometry heatmap showing H2BNTac levels in VCaP cells treated with DMSO, 100 nM DALTAC-1, or a combination of 100 nM p300/CBPi and ARi for 12 hours. Data represent n = 3 biological replicates. g. Volcano plots showing altered lysine acetylation levels in VCaP cells treated with 100 nM DALTAC-1 versus DMSO for 4 hours. H2BNTac sites are highlighted in blue, and components of the AR complex are highlighted in red. Data plotted from n=3 independent samples. Statistical tests were two-tailed t-tests. h. Acetyl-lysine mass spectrometry heatmap showing the top 50 acetyl-lysine sites most increased or decreased in VCaP cells treated with DMSO, 100 nM DALTAC-1, or a combination of 100 nM ARi and p300/CBPi for 3 hours. Data represent n = 3 biological replicates.

In contrast to the p300/CBP dual degrader CBPD-409, DALTAC-1 did not reduce p300 protein abundance; instead, it rapidly and robustly suppressed enhancer-associated histone acetylation marks, including H3K27ac and H2BNTac (H2BK20ac) (Fig. 2e). Notably, H2BNTac is a key chromatin mark of active enhancers in prostate cancer cells and is exclusively catalyzed by p300/CBP through bromodomain-independent activities^16,17^. Our data demonstrate that DALTAC-1 exerts a *de novo* heterobifunctional effect by potently suppressing this critical histone mark without degrading p300. This level of H2BNTac suppression was not observed with the inactive analogs Neg-1 and Neg-2, nor with the combination of p300/CBP and AR inhibitors (Extended Data Fig. 4a–c). In AR-negative yet p300/CBP-dependent Kelly neuroblastoma cells^43^, DALTAC-1 failed to abolish H2BNTac, confirming its lineage selectivity (Fig. 2f). Furthermore, in HEK293 cells, the degree of H2BNTac reduction by DALTAC-1 scaled proportionally with the level of ectopically expressed AR, reinforcing that both AR and p300/CBP are required to mediate DALTAC activity (Fig. 2g and Extended Data Fig. 4d). Consistent with its durable pharmacologic effect, a 2-hour pulse treatment of DALTAC-1 followed by compound washout resulted in robust inhibition of histone acetylation and MYC protein expression, whereas washout of the p300/CBP and AR inhibitor combination markedly attenuated these effects (Extended Data Fig. 4e).

Beyond histone substrates, p300/CBP also acetylate a range of non-histone proteins that regulate transcriptional complexes and signal transduction^44^. Previous studies using p300/CBP HAT inhibitors or PROTAC degraders demonstrated that acute p300 inhibition results in broad suppression of the cellular acetylome, reducing acetylation on both histone and non-histone targets^12,17^. Acetyl-lysine mass spectrometry (acetyl-K MS) profiling in VCaP cells revealed that DALTAC-1, unlike conventional p300/CBP inhibition, exerted a distinct acetylation signature. DALTAC-1 markedly suppressed global histone acetylation, most prominently the enhancer-associated H2BNTac mark, surpassing the effect of combined p300/CBP and AR inhibitors (Fig. 2h and Extended Data Fig. 4f, g). Consistent with this observation, immunoprecipitation-mass spectrometry analysis demonstrated that H2B was the most significantly displaced p300-interacting protein upon DALTAC-1 treatment (Extended Data Fig. 3b), indicating a selective disruption of p300’s association with its canonical histone substrate.

Unexpectedly, DALTAC-1 simultaneously induced specific non-histone acetylation events, particularly within components of the AR transcriptional complex. Prominent increases in acetylation were observed in AR as well as its cofactors SRC2, SRC3, ARID1A, and ARID1B^45,46^ (Fig. 2h, i and Extended Fig. 4g, h). These acetylation sites were located within the NTD of AR, which mediates AR–cofactor interactions^47–49^, suggesting that the robust ternary complex formation between AR and p300 redirects p300’s catalytic activity toward the AR-NTD and AR-associated cofactors. Importantly, such acetylation increases were not observed in cells treated with the combination of the p300/CBP inhibitor and AR inhibitor (Fig. 2h, i and Extended Fig. 4h). These combined data demonstrate that DALTAC-1 induces a selective acetylation pattern in AR-positive prostate cancer cells, characterized by strong suppression of enhancer-linked histone acetylation and concurrent increases in acetylation of AR and its associated cofactors.

### DALTAC-1 suppresses the oncogenic transcriptional program in AR-positive prostate cancer cells

**Extended Data Figure 5.**
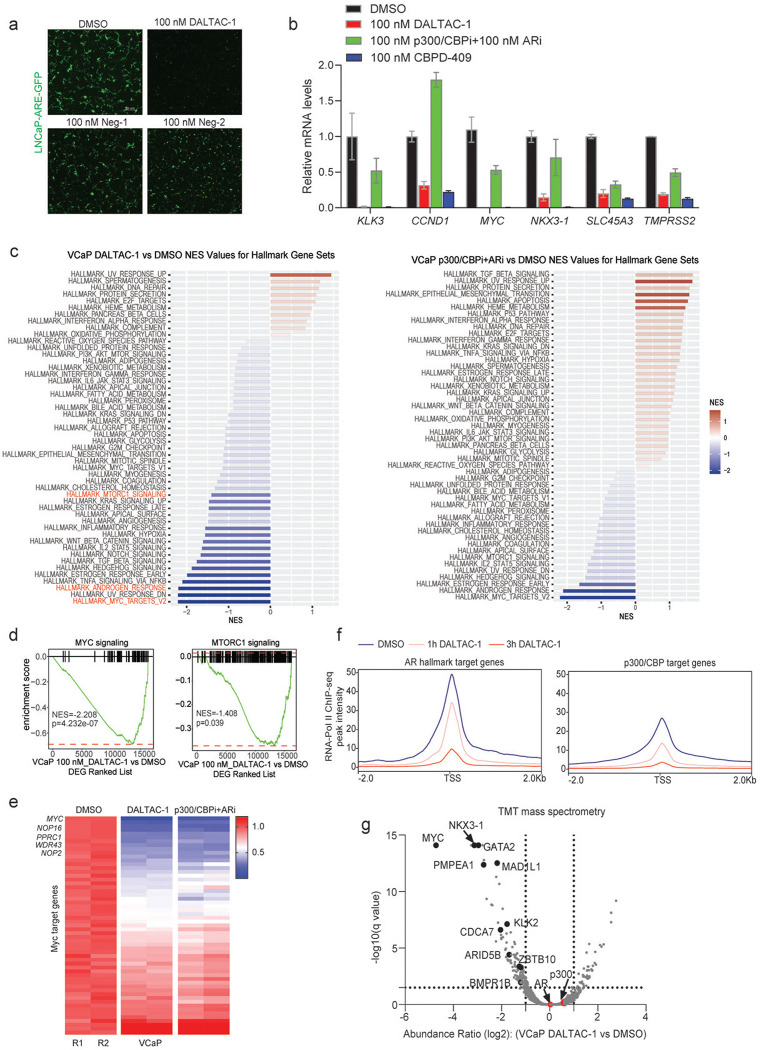
DALTAC-1 inhibits oncogenic transcriptional programs in prostate cancer cells. a. Representative fluorescence microscopy images of LNCaP-ARE-GFP cells treated with DMSO, 100 nM DALTAC-1, Neg-1, or Neg-2 for 48 hours. Scalebar = 50 μm. b. qPCR of *KLK3*, *MYC*, *CCND1*, *NKX3–1*, *SLC45A3,* and *TMPRSS2* expression in VCaP cells treated with DALTAC-1, combination of p300/CBPi and ARi, or CBPD-409 for 4 hours. n=3 biological replicates. c. GSEA net enrichment score (NES) plot of significantly altered hallmark pathways in VCaP cells treated with 100 nM DALTAC-1 or 100 nM combination of p300/CBPi and ARi for 4 hours. d. GSEA plots for MYC and MTORC1 pathway-related genes using the fold change rank-ordered gene signature from the DALTAC-1 treated VCaP cells. NES, net enrichment score; adj P, adjusted p-value; DEGS, differentially expressed genes. n=2 biological replicates. Statistical significance was assessed using a two-sided GSEA permutation test with adjustment for multiple comparisons. e. RNA-seq heatmaps for MYC target genes in VCaP cells treated with 100 nM DALTAC-1 or 100 nM combination of p300/CBPi and ARi for 4 hours. n=2 biological replicates. f. RNA polymerase II ChIP–seq signal intensities within ±2 kb of transcription start sites (TSS) of AR- and p300/CBP-regulated genes in VCaP cells treated with 100 nM DALTAC-1 for 1 hour and 3 hours. g. TMT (tandem mass tag)–based mass spectrometry analysis of VCaP cells treated with 100 nM DALTAC-1 for 12 hours. Data are presented as log2 fold change (FC) relative to DMSO control versus –log2 p-value for each protein, based on n = 3 independent experiments. Statistical significance was determined by two-tailed t-tests assuming equal variances. AR target proteins, MYC target proteins, as well as AR and p300 are highlighted.

Given the distinct reprogramming of p300/CBP substrate engagement by DALTAC-1, we next examined its impact on transcriptional output. An androgen response element (ARE) reporter assay demonstrated that DALTAC-1 potently suppressed AR transcriptional activity in LNCaP cells, producing a markedly greater reduction in ARE–GFP reporter signal compared with Neg-1 and Neg-2 (Fig. 2j and Extended Data Fig. 5a). Consistently, qPCR analysis showed strong repression of canonical AR target genes by DALTAC-1, whereas the combined inhibition of p300 and AR elicited only moderate effects (Extended Data Fig. 5b). To further investigate the global transcriptional effects of DALTAC-1, we performed RNA sequencing (RNA-seq) in VCaP cells. Gene set enrichment analysis (GSEA) revealed that DALTAC-1 profoundly downregulated hallmark pathways associated with androgen response, p300/CBP targets, MYC targets, and MTORC1 signaling (Fig. 2k, l and Extended Data Fig. 5c-e). These data indicate that DALTAC-1 preferentially represses AR- and p300/CBP-dependent oncogenic transcriptional circuits. Chromatin immunoprecipitation followed by sequencing (ChIP–seq) further demonstrated that DALTAC-1 rapidly displaced RNA polymerase II from AR and p300 target loci within hours of treatment, signifying prompt transcriptional shutdown (Extended Data Fig. 5f).

At the protein level, both DALTAC-1 and the p300/CBP degrader CBPD-409 markedly reduced PSA, CCND1, and NKX3–1 expression, whereas the combination of p300/CBP and AR inhibitors produced only modest effects (Fig. 2m). Quantitative proteomic profiling further confirmed that DALTAC-1 selectively downregulated AR and MYC-regulated proteins without altering the abundance of AR or p300 themselves (Extended Data Fig. 5g). Combined, these findings demonstrate that DALTAC-1 reprograms p300/CBP substrate utilization and enforces broad suppression of AR-driven oncogenic transcriptional programs in prostate cancer cells.

### DALTAC-1 reshapes the AR–p300 cistrome and remodels the oncogenic enhancer landscape

**Extended Data Figure 6.**
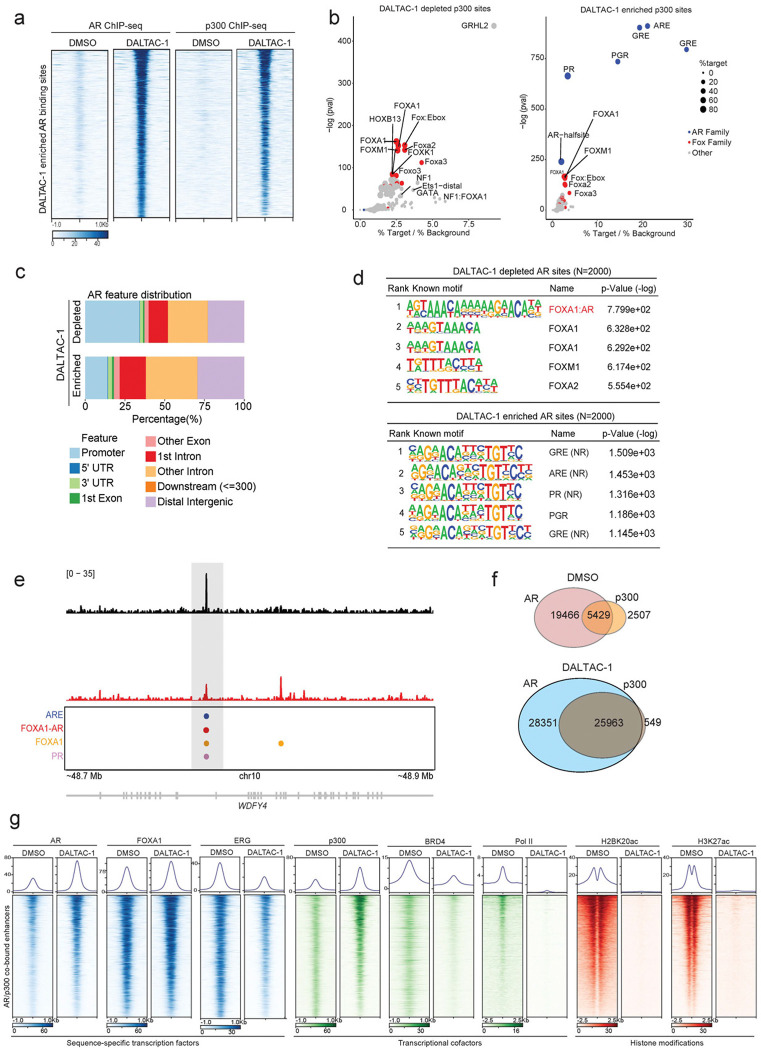
DALTAC-1 remodels the chromatin landscape of the AR neo-enhanceosome. a. ChIP-seq read-density heatmaps of p300 and AR at DALTAC-1 enriched AR binding sites in VCaP cells treated with DMSO or 100 nM DALTAC-1 for 3 hours. b. Analyses of %target vs %background and significance levels for HOMER motifs enriched at p300 non-promoter binding sites depleted by 100 nM DALTAC-1 (left) or enriched by 100 nM DALTAC-1 in VCaP cells. AR family motifs are highlighted in blue; FOX family motifs are highlighted in red. P-values were calculated using HOMER’s binomial test (two-sided) and adjusted for multiple comparisons using the Benjamini–Hochberg method. c. Genomic distribution of DALTAC-1 depleted AR ChIP-seq peaks and DALTAC-1 enriched AR peaks in VCaP cells. d. Top five known HOMER motifs enriched within top 2000 DALTAC-1 depleted or enriched AR sites in VCaP cells. P-values were calculated using HOMER’s binomial test (two-sided). e. AR ChIP–seq tracks on *WDFY4* gene loci in VCaP cells treated with 100 nM DALTAC-1 or DMSO for 3 hours. Highlighted motifs are shown in different colors. f. Venn diagram illustrating overlap of genome-wide AR and p300 ChIP-seq peaks in VCaP cells treated with DMSO (top) or 100 nM DALTAC-1 (bottom) for 3 hours. g. ChIP-seq read-density heatmaps of indicated transcription factors, transcriptional cofactors, and histone modifications at AR/p300 co-bound non-promoter sites in VCaP cells treated with DMSO or 100 nM DALTAC-1 for 3 hours.

**Figure 3.**
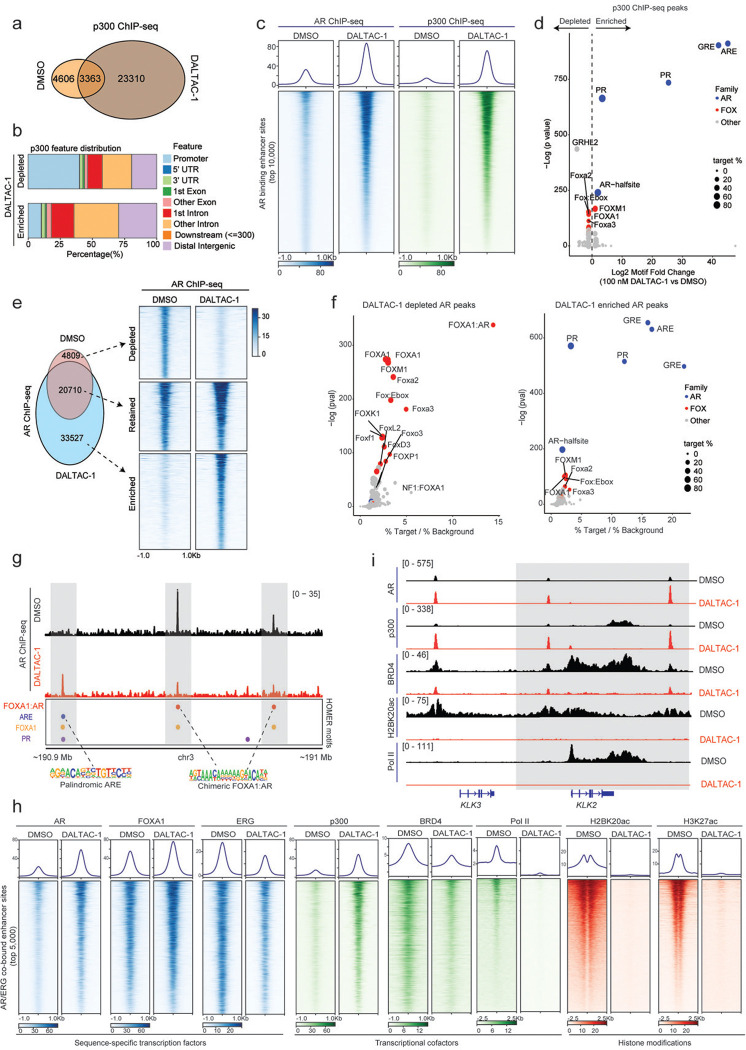
DALTAC-1 reshapes the AR–p300 cistrome and remodels the oncogenic enhancer landscape. a. Venn diagram illustrating overlap of genome-wide p300 ChIP-seq peaks in VCaP cells treated with DMSO or 100 nM DALTAC-1 for 3 hours. b. Genomic distribution of DALTAC-1 depleted p300 ChIP-seq peaks and DALTAC-1 enriched p300 ChIP-seq peaks in VCaP cells treated with DMSO of 100 nM DALTAC-1 for 3 hours. c. ChIP-seq read-density heatmaps of AR and p300 at AR binding enhancer sites in VCaP cells treated with DMSO or 100 nM DALTAC-1 for 3 hours. d. Fold-change and significance of HOMER motifs enriched within p300 non-promoter binding sites in 100 nM DALTAC-1 treated versus DMSO treated VCaP cells. AR family motifs are highlighted in blue; FOX family motifs are highlighted in red. P-values were calculated using HOMER’s binomial test (two-sided) and adjusted for multiple comparisons using the Benjamini–Hochberg method. e. Left: Venn diagram illustrating overlap of genome-wide AR ChIP-seq peaks in VCaP cells treated with DMSO or 100 nM DALTAC-1 for 3 hours. Right: ChIP-seq read-density heatmaps of AR in VCaP cells treated with DMSO or 100 nM DALTAC-1 for 3 hours. f. Analyses of %target vs %background and significance levels for HOMER motifs enriched at AR non-promoter binding sites depleted by 100 nM DALTAC-1 (left) or enriched by 100 nM DALTAC-1 in VCaP cells. AR family motifs are highlighted in blue; FOX family motifs are highlighted in red. P-values were calculated using HOMER’s binomial test (two-sided) and adjusted for multiple comparisons using the Benjamini–Hochberg method. g. AR ChIP–seq tracks on chromosome 3 (190.9–191.0 Mb) in VCaP cells treated with 100 nM DALTAC-1 or DMSO for 3 hours. Highlighted motifs are shown in different colors. h. ChIP-seq read-density heatmaps of indicated transcription factors, transcriptional cofactors, and histone modifications at AR/ERG co-bound non-promoter sites in VCaP cells treated with DMSO or 100 nM DALTAC-1 for 3 hours. i. ChIP–seq tracks of indicated proteins and histone mark on *KLK3* and *KLK2* gene loci in VCaP cells treated with 100 nM DALTAC-1 or DMSO for 3 hours.

p300 serves as a central coactivator that binds to active AR enhancer sites and catalyzes histone acetylation to sustain oncogenic transcriptional programs in prostate cancer cells^17^. To investigate how DALTAC-1 modulates p300 chromatin occupancy, we performed p300 ChIP–seq in VCaP cells. DALTAC-1 treatment led to a striking expansion of p300-binding events genome-wide (Fig. 3a). Genomic annotation revealed distinct redistribution patterns of p300 chromatin binding upon DALTAC-1 treatment. Among the DALTAC-1–depleted peaks, approximately 40% were located at promoter-proximal regions (Fig. 3b). In contrast, the DALTAC-1–gained peaks were predominantly mapped to intronic and distal intergenic regions, with only about 10% residing near promoters (Fig. 3b), indicating increased engagement of enhancer-associated chromatin. Since DALTAC-1 enforces AR–p300 proximity, we next evaluated how p300 occupancy changed at AR-binding sites. At AR-binding enhancer sites identified under DMSO (control) conditions, DALTAC-1 induced a marked increase in AR occupancy, accompanied by a proportional enhancement in p300 occupancy (Fig. 3c and Extended Data Fig. 6a). This reciprocal recruitment pattern suggests that DALTAC-1 acts as a molecular glue, stabilizing chromatin-anchored AR–p300 complexes. Motif analysis revealed that DALTAC-1–induced p300 peaks were dominated by canonical palindromic androgen response elements (AREs), which emerged as the most significantly enriched motif (Fig. 3d and Extended Data Fig. 6b). Glucocorticoid receptor (GR) and progesterone receptor (PR) motifs also appeared among the top hits, but given their near-identical sequence to AREs^50^, these likely represent the same AR-binding elements. Together, these findings demonstrate that DALTAC-1 amplifies and redistributes p300 enhancer occupancy through AR-dependent recruitment, likely reflecting DALTAC-1–induced alteration of domain–domain connectivity within AR and p300 that stabilizes a neomorphic ternary complex on enhancer chromatin.

We next investigated how DALTAC-1 reshapes the AR cistrome. Consistent with the strengthened AR–p300 interaction, DALTAC-1 markedly increased AR occupancy at palindromic AREs (Fig. 3e, f and Extended Data Fig. 6c, d). Unexpectedly, DALTAC-1 simultaneously reduced AR binding at FOXA1-AR chimeric motifs, the non-canonical ARE configurations previously reported to be associated with the AR neo-enhanceosome^51^ (Fig. 3f, g and Extended Data Fig. 6d, e). Thus, instead of uniformly increasing AR binding, DALTAC-1 redirects AR away from chimeric oncogenic enhancers and concentrates it at canonical AREs, effectively remodeling the AR enhancer network. In line with this selective redistribution, DALTAC-1 led to a profound loss in coactivator (BRD4, ERG) recruitment, RNA polymerase II loading, and histone acetylation (H3K27ac and H2BK20ac) at AR/p300 co-bound enhancers (Extended Data Fig. 6f, g), demonstrating that DALTAC-1 functionally decommissions the active AR transcriptional complex.

**Extended Data Figure 7.**
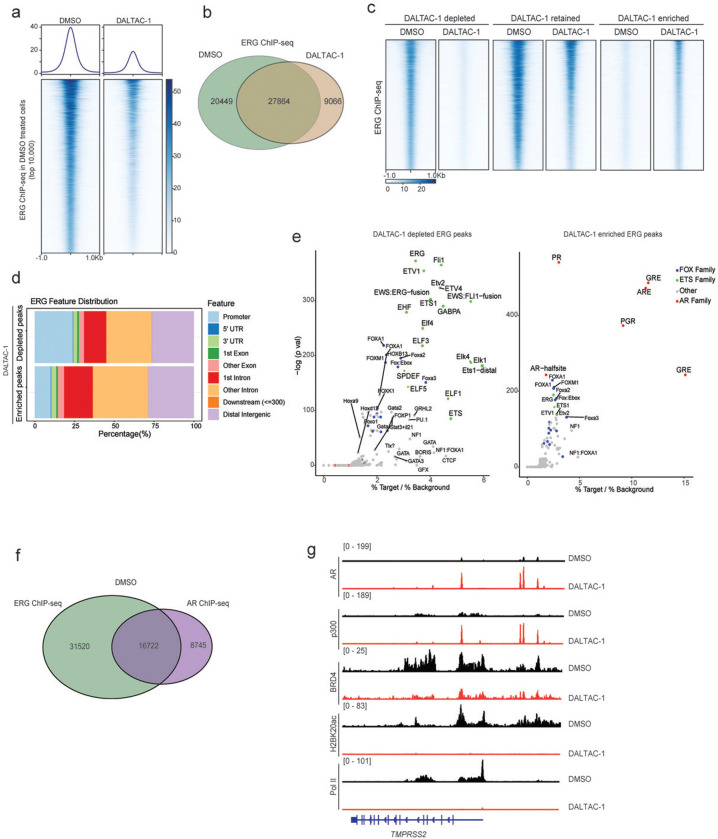
DALTAC-1 inhibits the activity of the AR/ERG neo-enhanceosome. a. ChIP-seq read-density heatmaps of ERG at top 10,000 ERG binding sites in VCaP cells treated with DMSO or 100 nM DALTAC-1 for 3 hours. b. Venn diagram illustrating overlap of genome-wide ERG ChIP-seq peaks in VCaP cells treated with DMSO or 100 nM DALTAC-1 for 3 hours. c. ChIP-seq read-density heatmaps of ERG at indicated sites in VCaP cells treated with DMSO or 100 nM DALTAC-1 for 3 hours. d. Genomic distribution of DALTAC-1 depleted ERG ChIP-seq peaks and DALTAC-1 enriched ERG peaks in VCaP cells. e. Analyses of %target vs %background and significance levels for HOMER motifs enriched at ERG non-promoter binding sites depleted by 100 nM DALTAC-1 (left) or enriched by 100 nM DALTAC-1 in VCaP cells. AR family motifs are highlighted in red; FOX family motifs are highlighted in blue; ETS family motifs are highlighted in green. P-values were calculated using HOMER’s binomial test (two-sided) and adjusted for multiple comparisons using the Benjamini–Hochberg method. f. Venn diagram illustrating overlap of genome-wide AR and ERG ChIP-seq peaks in VCaP cells treated with DMSO. g. ChIP–seq tracks of indicated proteins and histone mark on *TMPRSS2* gene loci in VCaP cells treated with 100 nM DALTAC-1 or DMSO for 3 hours.

Oncogenic transcription factors such as ERG and FOXA1 mutants are known to hijack AR to non-canonical AREs, forming cancer-specific AR neo-enhanceosomes that drive lineage-dependent oncogene programs^5,6,51,52^. To evaluate how DALTAC-1 influences this architecture, we examined its effects on AR/ERG neo-enhanceosomes in *TMPRSS2–ERG* fusion positive VCaP cells^53,54^. DALTAC-1 globally suppressed ERG chromatin occupancy (Extended Data Fig. 7a, b). Interestingly, a subset of ERG-binding peaks emerged following treatment, predominantly within distal intergenic and intronic regions (Extended Data Fig. 7b-d). Furthermore, motif analysis revealed that DALTAC-1 depleted ERG binding at canonical ETS motifs while redirecting a fraction of ERG binding toward palindromic AR motifs (Extended Data Fig. 7e), suggesting that ERG can be shifted from its native ETS-binding sites and tethered to AR-dominant enhancers within the reconfigured complex.

We next analyzed the chromatin state at the AR/ERG co-bound non-promoter regions, which represent the AR/ERG neo-enhancers (Extended Data Fig. 7f). DALTAC-1 markedly increased AR and p300 occupancy at these loci (Fig. 3h), consistent with its ability to stabilize AR–p300 interactions. In stark contrast, these same regions became transcriptionally inert: ERG and BRD4 binding were diminished, and, strikingly, RNA polymerase II occupancy and enhancer-associated histone acetylation marks were almost completely extinguished (Fig. 3h). The pronounced increase in AR/p300 occupancy alongside the sharp loss of ERG, BRD4, RNA polymerase II, and histone acetylation marks demonstrates that DALTAC-1 collapses AR–ERG neo-enhancers into an inactive epigenetic state, thereby suppressing ERG engagement and dismantling the transcriptional output of the AR–ERG neo-enhanceosome (Fig. 3i and Extended Data Fig. 7g).

### DALTAC-1 potently suppresses the growth of prostate cancer organoids

**Extended Data Figure 8.**
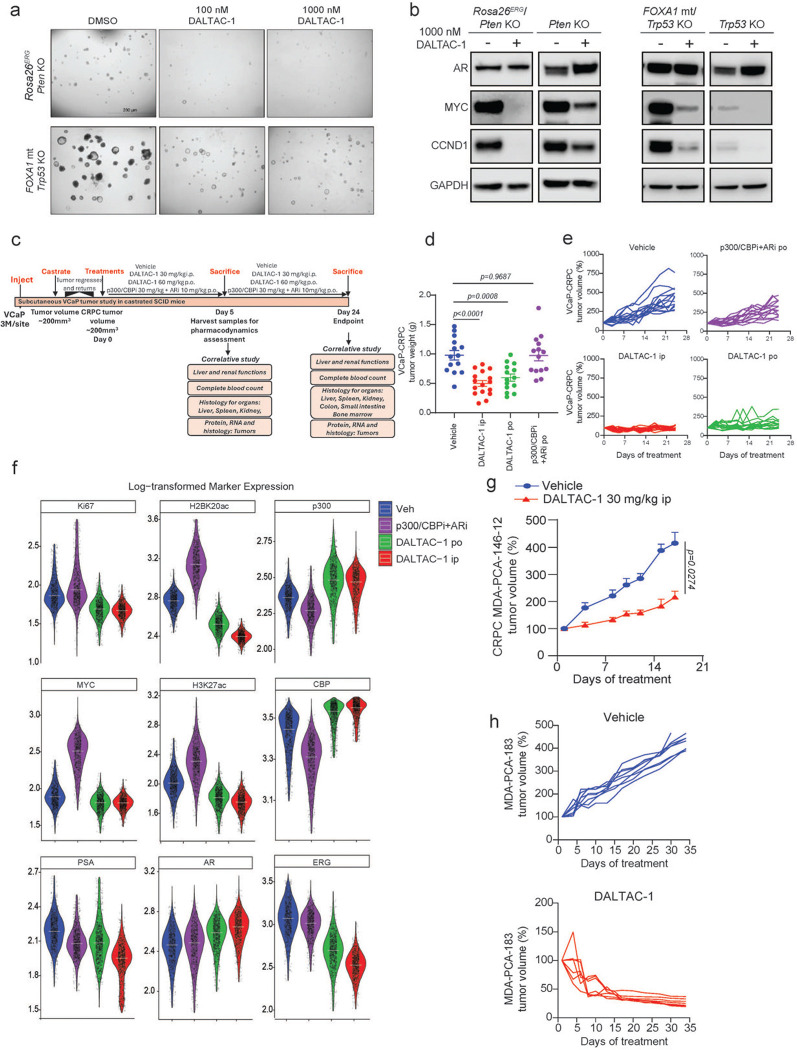
DALTAC-1 suppresses prostate cancer growth across multiple preclinical models. a. Representative images of *Rosa26*^*ERG*^*/Pten−/−* and *FOXA1* mt*/Trp53−/−* organoids treated with DMSO, 100 nM DALTAC-1, or 1000 nM DALTAC-1. Scalebar = 200 μm b. Immunoblot analysis of indicated proteins in *Rosa26*^*ERG*^*/Pten−/−, Pten−/−, FOXA1* mt*/Trp53−/−* and *Trp53−/−* organoids treated with DMSO or 1000 nM DALTAC-1. c. Schematic of the DALTAC-1 in vivo efficacy study utilizing the VCaP-CRPC xenograft model. d. Tumor weights in the VCaP-CRPC model treated with vehicle, DALTAC-1 ip, DALTAC-1 po, or combination of p300/CBPi and ARi po. Data are mean ± SEM; n = 16 per group. Two-sided t-test. e. Graph depicting the tumor volume curves of individual tumors from the VCaP-CRPC study. f. Violin plots showing the log10-transformed expression levels of individual targets across single cells based on Seq-mIF staining from PD5 tumors in the VCaP-CRPC study (n > 30,000 cells). The violin outline represents the kernel density estimate; grey dots indicate individual cells; and the central white line denotes the median. g. Graph depicting the tumor volume curves of MDA-PCA-146–12 CRPC PDX tumors treated with vehicle or 30 mg/kg DALTAC-1 ip. Data are presented as mean +/− SD (vehicle: n = 16, DALTAC-1 ip: n=7). P values were calculated using a two-sided t-test. h. Graph depicting the tumor volume curves of individual tumors from MDA-PCA-183 PDX study.

**Figure 4.**
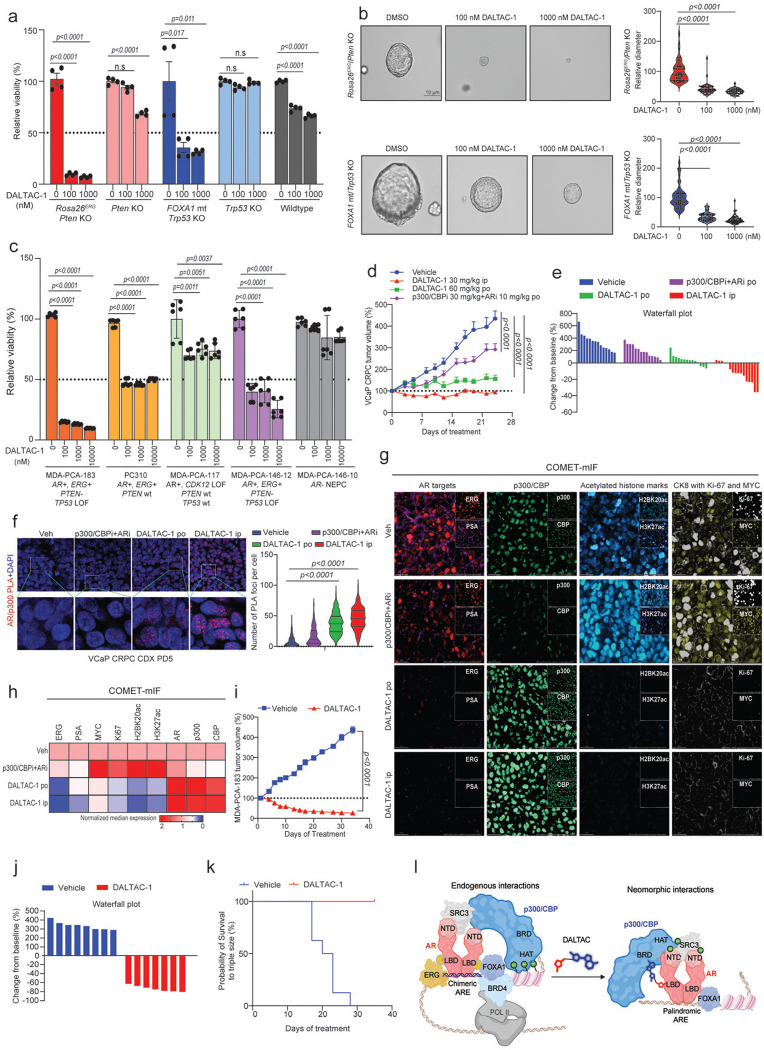
DALTAC-1 potently suppresses prostate cancer growth in preclinical models. a. Bar charts show the relative viability of indicated mouse organoids (derived from respective GEMM models) treated with DMSO, 100 nM DALTAC-1, or 1000 nM DALTAC-1 for 7 days. P values were calculated using a two-sided t-test. n=4 biologically replicated wells. b. Left: representative images of *Rosa26*^*ERG*^*/Pten*^*−/−*^ and *FOXA1* mt*/Trp53*^−/−^ organoids treated with DMSO, 100 nM DALTAC-1, or 1000 nM DALTAC-1 for 7 days. Right: quantification of relative diameters of results from left. P values were calculated using a two-sided t-test (n = 50 organoids). Scalebar = 10 μm. c. Bar charts showing the relative viability of indicated human patient-derived xenograft (PDX) organoids treated with DMSO, 100 nM DALTAC-1, 1000 nM DALTAC-1, or 10000 nM DALTAC-1 for 14 days. P values were calculated using a two-sided t-test. n=6 biologically replicated wells. d. Tumor volume curves and weights in the VCaP-CRPC model treated with vehicle, DALTAC-1 (30 mg/kg, ip, 5×/week during the first week, then 3×/week for an additional 3 weeks), DALTAC-1 (60 mg/kg, po, 5×/week during the first week, then 3×/week for an additional 3 weeks), or combination of p300/CBPi and ARi (30 mg/kg and 10 mg/kg respectively, po, 5x/week). Data are mean ± standard error of the mean (SEM); n = 16 per group. P values were calculated using a two-sided t-test. e. Waterfall plot showing the change in tumor volume after 24 days of treatment from the VCaP-CRPC study. f. Left: representative images of AR–p300 PLA assays in PD5 tumor samples from the VCaP-CRPC study. Right: quantification of AR–p300 PLA foci per cell (n=200 cells). P values were calculated using a two-sided t-test. Violin plot: central line, median; dotted lines, 25th–75th percentiles; violin, kernel density estimate; whiskers, minimum–maximum values. g. Representative COMET-mIF merged images (ERG, PSA, p300, CBP, H2BK20ac, H3K27ac, MYC, ki67 and CK8) from PD5 tumors in the VCaP-CRPC study. Scalebar = 20 μm. h. Heatmap showing normalized median expression levels of the indicated targets derived from the COMET-mIF analyses. i. Tumor volume curves in the MDA-PCA-183 PDX model treated with vehicle or DALTAC-1 (30 mg/kg, ip, 5×/week during the first week, then 2×/week for an additional 4 weeks). Data are mean ± SEM; n = 8 in vehicle group and n=7 in DALTAC-1 treated group. P values were calculated using a two-sided t-test. j. Waterfall plot showing the change in tumor volume after 35 days of treatment from the MDA-PCA-183 study. k. Kaplan–Meier survival curve of MDA-PCA-183 PDX–bearing mice, with survival defined as the time for tumors to reach threefold the initial volume. l. Proposed mechanism of action of AR–p300/CBP DALTAC-1. In prostate cancer cells, AR binds to chimeric AREs, and the AR-NTD interacts with p300/CBP and SRC3 to recruit transcriptional coactivators, assemble the AR neo-enhanceosome, and catalyze histone acetylation to activate oncogene expression. DALTAC-1 rewires the AR-LBD to engage the p300/CBP BRD, redirecting AR binding toward palindromic AREs, displacing coactivators from chromatin, and erasing histone acetylation marks, ultimately leading to collapse of the AR neo-enhanceosome.

To assess the efficacy of DALTAC-1, we interrogated distinct molecular subtypes of prostate cancer represented by organoids generated from genetically engineered mouse models (GEMMs). We treated organoids derived from the prostate tissues of wild-type, *Rosa26*^*ERG*^*/Pten*^*−/−*^, *Pten*^−/−^, *FOXA1 R265–71del*^*+/+*^*/Trp53*^−/−^ (class1 FOXA1 mutant), and *Trp53*^−/−^ transgenic animals with DALTAC-1^52,55–57^. Compared to wild-type, *Pten*^−/−^, and *Trp53*^−/−^ organoids, DALTAC-1 markedly suppressed the growth of *Rosa26*^*ERG*^*/Pten*^*−/−*^ and *FOXA1 R265–71del*^*+/+*^*/Trp53*^−/−^ organoids, leading to a significant reduction in both organoid number and size (Fig. 4a, b and Extended Data Fig. 8a). Correspondingly, DALTAC-1 treatment potently downregulated key oncogenes, including MYC and CCND1, in these lineage-defined organoids relative to *Pten*^−/−^ and *Trp53*^−/−^ controls (Extended Data Fig. 8b). Given that ERG and mutant FOXA1 enhance AR activity and sustain the AR neo-enhanceosome^7,52,55,58^, these findings suggest that DALTAC-1 effectively suppresses the AR neo-enhanceosome driving prostate cancer.

To further evaluate DALTAC-1 activity in human prostate cancer models, we treated a panel of organoids derived from patient-derived xenograft (PDX) tumors with DALTAC-1^59,60^. Notably, DALTAC-1 exhibited robust growth inhibitory effects in AR-positive, particularly *AR*⁺/*ERG*⁺, organoids, aligning with its potent blockade of the AR neo-enhanceosome (Fig. 4c). In contrast, AR-negative neuroendocrine prostate cancer (NEPC) organoids displayed minimal response, consistent with the AR-dependent mechanism of action of DALTAC-1 (Fig. 4c). These results establish DALTAC-1 as a potent suppressor of AR-driven prostate cancer growth across both murine and human organoid models, highlighting its broad yet lineage-selective anti-tumor activity.

### DALTAC-1 exhibits potent in vivo efficacy against prostate cancer

To evaluate the anti-tumor activity of DALTAC-1, we employed a VCaP castration-resistant prostate cancer (VCaP-CRPC) xenograft model established by subcutaneous implantation of VCaP cells into castrated male NSG mice (Extended Data Fig. 8c). Once tumors reached ~150 mm^3^, mice were randomized and treated with DALTAC-1 via intraperitoneal (ip) injection (30 mg/kg) or orally (po) (60 mg/kg). For comparison, a combination of a p300 bromodomain inhibitor and an AR-LBD inhibitor was administered at equipotent doses. IP administration of DALTAC-1 as a single agent elicited markedly superior anti-tumor activity, inducing tumor regression in 75% (12 of 16) of treated tumors (Fig. 4d, e and Extended Data Fig. 8d, e). Oral dosing of DALTAC-1 also achieved robust tumor suppression and outperformed the dual inhibitor combination, underscoring its favorable translational properties (Fig. 4d, e and Extended Data Fig. 8d, e). Mechanistically, in vivo PLA revealed a marked increase in AR–p300 proximity in DALTAC-1–treated tumors, providing direct evidence for induced formation of the AR–p300–DALTAC-1 ternary complex in vivo (Fig. 4f). Consistently, COMET multiplex immunofluorescence (COMET-mIF) analyses^61^ of VCaP-CRPC tumors collected after five days of treatment revealed marked reductions in ERG, PSA, MYC, Ki67, H3K27ac, and H2BK20ac signals, while AR, p300, and CBP levels did not decrease, consistent with the mechanism of action of DALTAC-1 (Fig. 4g, h and Extended Data Fig. 8f).

**Extended Data Figure 9.**
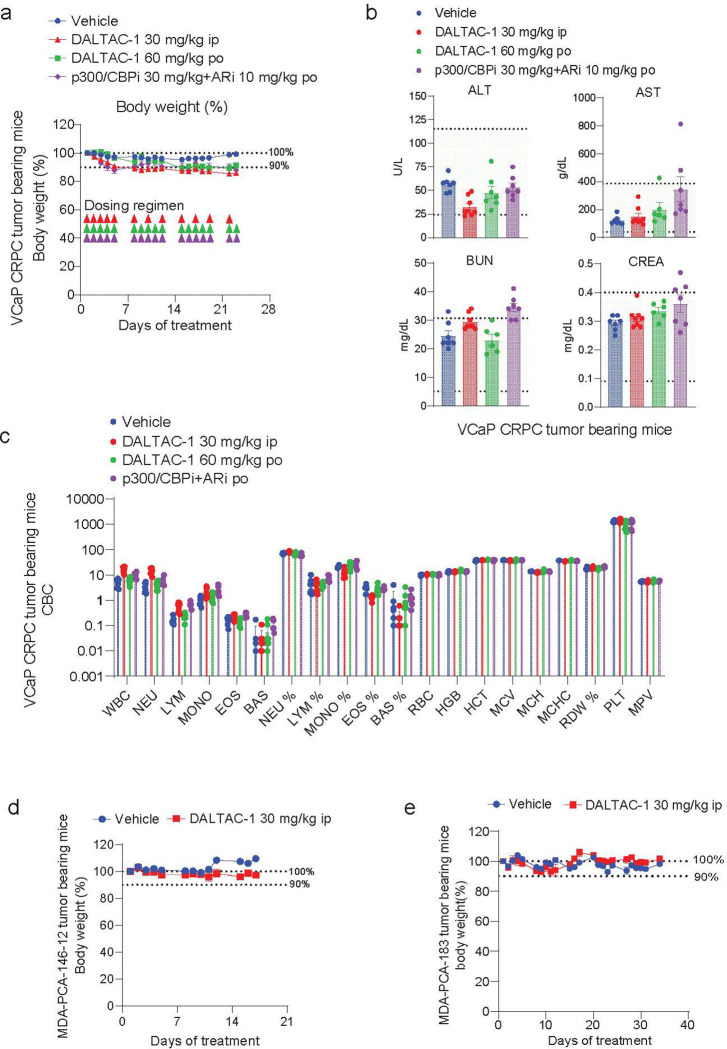
DALTAC-1 exhibits no apparent toxicity in tumor-bearing mice. a. Mice body weight (%) measurements throughout the treatment period from VCaP-CRPC study (two-sided t-test). Data are presented as mean +/− SEM (vehicle: n = 7, DALTAC-1 IP: n=8, DALTAC-1 PO: n=8, p300/CBPi+ARi: n=7). b. Liver function and kidney function tests from VCaP-CRPC mouse study. Data are presented as mean +/− SD (vehicle: n = 7, DALTAC-1 IP: n=8, DALTAC-1 PO: n=8, p300/CBPi+ARi: n=7). c. Complete blood count from VCaP-CRPC mice. Data are presented as mean +/− SD (vehicle: n = 7, DALTAC-1 IP: n=8, DALTAC-1 PO: n=8, p300/CBPi+ARi: n=7). d. Mice body weight (%) measurements throughout the treatment period from MDA-PCA-146–12 CRPC study, Data are presented as mean +/− SEM (vehicle: n = 9, DALTAC-1 ip: n=4). e. Mice body weight (%) measurements throughout the treatment period from MDA-PCA-183 study. Data are presented as mean +/− SEM (vehicle: n = 4, DALTAC-1 ip: n=5).

To extend these findings to clinically relevant models, we assessed the efficacy of DALTAC-1 in two PDX models, MDA-PCa-146–12 and MDA-PCa-183^59^. Consistent with the lineage-selective activity observed in organoids, DALTAC-1 treatment significantly inhibited tumor growth in both models (Fig. 4i and Extended Data Fig. 8g). The response in MDA-PCa-183 was particularly striking. This is a treatment-naïve, bone metastatic prostate cancer PDX model harboring a TMPRSS2–ERG fusion, TP53 loss-of-function, and PTEN loss.. All treated mice showed rapid and consistent tumor shrinkage, and every tumor fully regressed during the treatment window (Fig. 4i-j and Extended Data Fig. 8h). The survival analysis therefore remained at 100% for all treated mice, with no signs of progression after 35 days of treatment (Fig. 4k). Importantly, throughout all the studies, DALTAC-1 was well tolerated, with no significant changes in body weight, serum liver or kidney function, or hematologic parameters (Extended Data Fig. 9a-e). Together, these results demonstrate that DALTAC-1 is an effective and well-tolerated anti-tumor agent that exerts durable efficacy across multiple prostate cancer models through selective AR–p300 domain alterations and chromatin reprogramming, establishing it as a promising first-in-class therapeutic with strong potential for clinical translation.

## Discussion

Recent advances in chemical-induced proximity (CIP) have led to the development of a diverse array of compounds capable of modulating protein functions through enforced protein–protein interactions^62,63^. For instance, RIPTACs induce proximity between a target protein and a pan-essential effector that disrupts the essential effector’s function, leading to selective cancer cell death while sparing healthy cells^30,31^. In contrast, TCIPs recruit transcriptional coactivators to specific genomic loci, paradoxically activating the target pathway by enhancing transcription of pro-apoptotic genes normally repressed by corepressors such as BCL6^32,33^. These contrasting mechanisms illustrate that bringing two unrelated proteins into proximity can yield distinct outcomes. In the present study, we described the development of the DALTAC strategy. Instead of forcing proximity between unrelated proteins, DALTACs perturb interactions between protein partners that normally function together by inducing a non-native domain configuration. Therefore, DALTACs act as allosteric saboteurs—throwing a molecular “monkey wrench” into essential protein assemblies, and in doing so, disable the normal function of the complex. Our proof-of-concept compound, AR–p300/CBP DALTAC-1, targets the AR–p300 complex by repurposing their natural interfaces to block activation of the AR oncogenic enhanceosome. Mechanistically, DALTAC-1 potently suppresses AR transactivation by disrupting the chromatin landscape at AR-bound enhancer sites, particularly the AR/ERG co-bound neo-enhancer sites, as evidenced by reduced cofactor recruitment, loss of histone acetylation marks, and displacement of RNA polymerase II. Functionally, this shutdown of AR neo-enhanceosome activity translates into strong anti-tumor efficacy in AR-positive prostate cancer models, underscoring the translational potential of DALTAC-1 as a precision therapeutic.

The activities of multi-protein transcriptional complexes are governed by finely tuned protein–protein interactions (PPIs) at the sub-domain level^21,64^. As a master transcription factor driving prostate cancer progression, the AR-NTD serves as a dynamic platform for coactivator recruitment^25,65^. The catalytic module of p300, comprising its BRD and HAT domain, resides within the central region of the protein^66^, yet distinct transcription factors engage p300 through its N- or C-terminal domains to facilitate histone acetylation and transcriptional activation^26,67^. Our results show that DALTAC-1 effectively redirects AR–p300 interactions, inhibiting p300 binding to the AR-NTD while enhancing its interaction with the AR-LBD. Remarkably, DALTAC-1 strongly suppresses histone protein acetylation, including H2BNTac levels, even though its p300-binding moiety is a BRD inhibitor incapable of directly blocking this modification^17^. In parallel, DALTAC-1 induces acetylation of the AR-NTD and core components of the AR complex, including SRC2/3 and ARID1A/B^2,45,68^. Notably, previous studies have shown that p300 acetylates AR primarily at the hinge domain, not the NTD, under androgen stimulation^69,70^. Thus, the emergence of AR-NTD acetylation in DALTAC-1–treated cells suggests that the compound creates a non-canonical acetylation pattern, potentially through a secondary, DALTAC-induced proximity between the AR-NTD and the p300 HAT domain. This “substrate reprogramming” represents a unique mechanism by which DALTAC-1 interferes with p300 activity, offering advantages over conventional p300 inhibitors or degraders. Unlike BRD inhibitors, DALTAC-1 abolishes enhancer-associated H2BNT acetylation, resulting in comprehensive transcriptional repression. Moreover, unlike PROTAC degraders, DALTAC-1 acts independently of the ubiquitin-proteasome system, ensuring rapid onset of action and mitigating resistance associated with E3-ligase loss or mutations^71,72^.

Because many oncogenes also play essential roles in normal physiology, their pan-inhibition often causes severe side effects^73^. For example, systemic inhibition of p300 disrupts hematopoiesis and leads to thrombocytopenia^20^. Thus, achieving lineage-selective targeting of oncogenic pathways is an urgent therapeutic need. Existing approaches such as antibody–drug conjugates (ADCs) deliver cytotoxic payloads selectively to cells expressing target surface antigens^74^. However, their broader applicability is limited by the restricted repertoire of tumor-specific surface markers and, in many cases, by suboptimal pharmacokinetic properties^75,76^. By contrast, DALTACs offer a fundamentally distinct strategy, selectively rewiring lineage-specific protein complexes that drive oncogenic transcription. In this study, DALTAC-1 specifically inhibits the growth of AR-positive prostate cancer cells with potency comparable to or better than the p300/CBP degrader CBPD-409, but shows dramatically reduced activity in p300/CBP-dependent, AR-negative cancers such as neuroblastoma^43^, Ewing sarcoma^77^, and hematologic malignancies^78,79^. Notably, in AR-negative cells, DALTAC-1 fails to suppress H2BNTac, underscoring that DALTAC-1–mediated inhibition of p300 histone acetylation is context-dependent and conferred by domain reprogramming within AR-p300 complexes.

In summary, we establish DALTACs as a new type of chemical proximity modulators that rewire domain-specific protein interactions to selectively deactivate oncogenic complexes. The prototype molecule, AR–p300/CBP DALTAC-1, exhibits potent, rapid, and lineage-restricted inhibition of AR-driven transcription and tumor growth. Mechanistic studies reveal that DALTAC-1 induces formation of an AR-p300-DALTAC-1 ternary complex and reprograms the substrate spectrum of p300, markedly suppressing global histone acetylation while enhancing the acetylation of core components within the AR complex. Consequently, DALTAC-1 reinforces AR/p300 co-occupancy at palindromic ARE loci but disrupts the higher-order assembly of the AR complex and erases histone activation marks at AR neo-enhanceosomes (Fig. 4l). This work expands the conceptual framework of induced-proximity therapeutics beyond degradation and coactivator hijacking, paving the way for the rational design of domain-alteration strategies for precise control of disease-relevant protein complexes. Future optimization of DALTAC pharmacologic properties and extension of this approach to other lineage-specific transcriptional networks may enable a new generation of selective, mechanism-guided cancer therapies.

## Methods

### Cell lines

All cell lines used in this study were originally sourced from ATCC, DSMZ, ECACC, Lonza, or maintained as authenticated internal stocks. The CWR-R1 prostate cancer line was kindly provided by Dr. David Vander Griend (University of Illinois at Chicago). Cell line authentication was performed semi-annually through genotyping at the University of Michigan DNA Sequencing Core, and routine Mycoplasma testing was carried out biweekly. LNCaP, 22Rv1, CWR-R1, and DU145 cells were cultured in RPMI-1640 medium supplemented with 10% fetal bovine serum (FBS; Thermo Fisher Scientific). VCaP cells were maintained in DMEM GlutaMAX supplemented with 10% FBS (Thermo Fisher Scientific).

### Chemistry

Detailed synthesis and chemical data for all designed DALTAC molecules and negative controls are provided in the Supplementary Notes.

### Antibodies

For immunoblotting, the following antibodies were used: p300 (Invitrogen: MA1–16608); AR (Abcam: ab133273); H2BK20ac (Cell Signaling Technology: 34156S); H2BK16ac (Abcam: ab177427); H3K27ac (Cell Signaling Technology: 8173S); H2B (Active Motif: 39210); H3 (Cell Signaling Technology: 3638S); c-Myc (Cell Signaling Technology: 9402S); c-Myc/N-Myc (Cell Signaling Technology: 13987S); KLK3/PSA (Dako: A0562); NKX3–1 (Cell Signaling Technology:83700S); CCND1 (Abcam: ab16663); Vinculin (Cell Signaling Technology: 18799S); GAPDH (Santa Cruz Biotechnology: sc-47724); BRD4 (Bethyl Laboratories: A700–004CF), HA-Tag (Cell Signaling Technology: 3724S).

For IP and ChIP-seq, the following antibodies were used: H3K27ac (Diagenode: C15410196); p300 (active motif: 61401); H2BK20ac (Cell Signaling Technology: 34156S); AR (Abcam: ab108341); FOXA1 (Thermo Fisher Scientific: PA5–27157); BRD4 (Diagenode: C15410337); ERG (Cell Signaling Technology: 97249S); RNA Pol II (Diagenode: C15200253); acetylated-lysine (Cell Signaling Technology: 9441S)

For immunofluorescent staining (COMET-mIF) and PLA assay, the following antibodies were used: p300 (Invitrogen: MA1–16608); CBP (Invitrogen: PA5–27369); AR (Abcam: ab108341); H3K27ac (Cell Signaling Technology: 8173S); Ki67 (Abcam: ab243878); H2BK20ac (Abcam: ab177430); CK8 (Abcam, ab53280); ERG (Abcam, ab92513); PSA (Cell Marque, 324M-16); MYC (Abcam, ab32072).

For ELISA, the following antibodies were used: p300 (Cell Signaling Technology: 86377S); AR (Thermo Fisher Scientific: MA5–13426).

### Cell viability assay

VCaP cells were seeded at a density of 10,000 cells per well, and all other prostate cancer cell lines were seeded at 3,000 cells per well in poly-D-lysine–coated 96-well plates (Corning) and maintained at 37 °C with 5% CO_2_. After 24 hours, cells were treated with a serial dilution of test compounds, with six replicate wells for each concentration. Following 120 hours of incubation, cell viability was determined using the CellTiter-Glo Luminescent Cell Viability Assay (Promega) according to the manufacturer’s instructions. Luminescence was measured on an Infinite M1000 Pro plate reader (Tecan), and data were analyzed using GraphPad Prism (GraphPad Software).

### Incucyte proliferation assays

A total of 4,000 VCaP cells or 1,500 LNCaP cells per well were seeded into poly-D-lysine–coated 96-well plates. After 24 hours of incubation, cells were treated with a fixed concentration (100 nM) or serial dilutions of compounds and incubated for 4 hours. The medium was then removed, and cells were washed three times with PBS before fresh medium containing either vehicle or compounds was added. Phase-contrast images were collected every 4 hours, and cell proliferation was quantified by measuring the percentage of confluence (phase object area).

### Immunoblotting

Cells were lysed in RIPA buffer (Thermo Fisher Scientific) supplemented with 1× Halt^™^ Protease Inhibitor Cocktail (Thermo Fisher Scientific) and denatured in NuPAGE^™^ 1× LDS sample buffer containing a reducing agent (Invitrogen) by incubation at 70 °C for 15 minutes. Protein concentrations were determined using the Pierce^™^ BCA Protein Assay Kit (Thermo Fisher Scientific). Equal amounts of protein (15–30 μg) were resolved on NuPAGE^™^ 3–8% Tris-Acetate or 4–12% Bis-Tris protein gels (Thermo Fisher Scientific) and transferred onto 0.45-μm nitrocellulose membranes (Thermo Fisher Scientific) using the iBlot^™^ 3 Dry Blotting System (Thermo Fisher Scientific). Membranes were blocked in TBS-T (Tris-buffered saline with 0.1% Tween-20) containing 5% non-fat dry milk for 1 hour at room temperature and incubated overnight at 4 °C with primary antibodies. After washing, membranes were incubated with HRP-conjugated secondary antibodies and imaged using an Odyssey CLx Imager (LI-COR Biosciences).

### Pharmacokinetics study

Pharmacokinetic (PK) studies were performed by Shanghai Medicilon Inc (Shanghai, China) under the institutional-approved animal protocol. Specific pathogen-free male ICR mice were purchased from Shanghai Xipuer-BK Laboratory Animal Co., Ltd. Each compound was dosed in solution formulation for both intravenous and oral administration with the following formulation: 5% DMSO, 10% Solutol, and 85% normal saline. Blood samples were collected at 0.083, 0.25, 0.5, 1, 2, 4, 8, and 24 hours for intravenous administration, 0.25, 0.5, 1, 2, 4, 6, 8, and 24 hours for oral administration. Blood samples were collected from sets of three mice for each dosing group at each time point in labeled microcentrifuge tubes containing heparin sodium as an anticoagulant. Plasma samples were separated by centrifugation (2−8 ℃, 6800 g for 6 minutes) within 1 hour and stored at −80 ℃ until bioanalysis. All samples were processed for analysis by precipitation using acetonitrile and analyzed with a partially validated LC/MS/MS method. Pharmacokinetic parameters were calculated using the noncompartmental analysis tool of WinNonlin Enterprise software.

### Fluorescence polarization (FP) binding assays

The binding affinities of test compounds to the androgen receptor (AR) and CBP/p300 were measured using fluorescence polarization (FP)–based competitive binding assays. AR binding was assessed using the PolarScreen^™^ Androgen Receptor Competitor Assay Kit (Thermo Fisher Scientific, Cat. #A15880) following the manufacturer’s instructions.

CBP/p300 FP assays were performed in 96-well plates (MTP96VPWB-EP, Eppendorf) using a CLARIOstar microplate reader (BMG Labtech). Serial dilutions of test compounds were incubated with fluorescein-labeled tracer (2 nM final) and CBP/p300 protein (20 nM final) in assay buffer (PBS pH 7.4 supplemented with 0.01% BGG, 0.01% Tween-20, and 2 mM DTT) to a final reaction volume of 100 μL per well. Plates were mixed for 15 minutes and incubated at room temperature for 1 hour to reach equilibrium. Fluorescence polarization (mP units) was measured at 485 nm excitation and 530 nm emission.

For both AR and CBP/p300 assays, data analysis was performed in GraphPad Prism 10 using nonlinear regression. IC_50_ values were determined by fitting competition curves, and K values for competitive inhibitors were derived using nonlinear regression based on the tracer K and the concentrations of tracer and protein in the reaction. All FP experiments were conducted in duplicates across three independent biological replicates.

### Computational modeling

All modelling and simulations were performed with Schrödinger 2025–2 (Schrödinger, LLC). Because co-crystal structures of the androgen receptor (AR) ligand-binding domain (LBD) in an open antagonist conformation were not available, we used our previously reported pseudo-open model^80^. The bound AR ligand was modified to generate JZY3221, the AR ligand used in our design of DALTACs. To model the p300 bromodomain interaction with GNE-049, we used the CBP bromodomain GNE-781 co-crystal structure (PDB 5W0E) as the starting structure^81^. CBP and p300 are highly homologous proteins, especially in their bromodomains which share conserved sequence and structures. Both proteins were prepared for further modeling using the Protein Preparation Wizard^82^.

We employed the following structures as inputs to generate the initial AR:DALTAC-1:p300 complex: (i) the AR in complex with JZY3221, (ii) the p300 complex with GNE-049, and (iii) JZY3032 (DALTAC-1) prepared with LigPrep module^83^. These inputs were submitted to Generate Degrader Complex (default settings: MCMM 10 000 steps, maximum clashes 5, maximum poses 50) to construct the ternary AR:JZY3032:p300 complex. The top 20 predicted complexes were further refined using Prime^84^. The top-scoring ternary complex was then selected for molecular dynamics (MD) simulations.

To perform MD simulations, the predicted ternary complex was solvated in an explicit water box with periodic boundary conditions. A cubic box was constructed with a 10 Å buffer between solute and box edge and filled with TIP4P water^85^. The OPLS4 force field was used for all components. Production dynamics used a RESPA integrator with a 2 fs time step.

Temperature was maintained at 300 K with a Nosé–Hoover chain thermostat (relaxation time 1.0 ps)^86^. Pressure was controlled at 1 atm with a Martyna–Tobias–Klein barostat (relaxation time 2.0 ps)^87^. Long-range electrostatics used a 9 Å real-space cutoff with particle–mesh Ewald for reciprocal space^88^. One continuous 1 μs simulation was performed without restraints.

Root-mean-square deviation (RMSD) analysis of protein backbones and ligand position indicated that the system equilibrated after approximately 750 ns. Accordingly, trajectory analysis focused on the last 250 ns. Protein–protein interface features were defined as minimum heavy-atom distances between AR and p300. Contacts shorter than 0.5 nm (5 Å) were retained. Principal component analysis (PCA) was applied to these interface distances, and components explaining 95% of the variance were kept. The reduced data were clustered with HDBSCAN (min_cluster_size 25, centroids enabled)^89^. For each cluster, the frame closest to the centroid in PCA space was saved as a representative PDB. Cluster occupancies are reported as percentages of frames per cluster. Protein–protein interaction fingerprints were computed with Schrödinger’s analyze_trajectory_ppi.py. Clustering in this reduced dimensional space revealed two dominant populations, with the most prevalent state comprising nearly 80% of the ensemble. The interaction analysis was performed on snapshots representative of this dominant state.

### Proximity ligation assay (PLA)

PLA was performed according to the manufacturer’s instructions (Navinci Diagnostics) using the Naveni^™^ TriFlex Cell kit for cultured cells (AR–p300) and the NaveniFlex^™^ Tissue Red kit for VCaP-CRPC tumor sections.

Cultured cells (Naveni TriFlex Cell): Cells were fixed, permeabilized, and blocked per kit protocol instructions. Primary antibodies against AR (1:400) and p300 (1:200) were applied in antibody diluent and incubated as recommended. After washes, Naveni proximity probes were added, followed by ligation and rolling-circle amplification steps according to the vendor’s workflow. Slides were counterstained with DAPI and mounted in antifade medium. PLA signals were visualized on a Zeiss LSM900 fluorescence microscope under identical exposure settings across conditions.

FFPE tumor sections (NaveniFlex Tissue Red): Sections were deparaffinized, rehydrated, and subjected to heat-induced epitope retrieval as specified by the kit. After blocking, primary antibodies against AR (1:1000) and p300 (1:100) were applied in antibody diluent and incubated per protocol. Following washes, NaveniFlex proximity probes, ligation, and amplification were performed according to the manufacturer’s instructions. Nuclei were counterstained with DAPI, slides were mounted, and images were acquired on a Zeiss LSM900 microscope using matched acquisition parameters.

Where indicated, PLA puncta were quantified as foci per nucleus using standardized thresholds across groups.

### Ternary complex ELISA assay

VCaP cells were plated in 96-well plates (50,000 cells per well) and cultured for 3 days in medium containing 5% charcoal-stripped FBS. Cells were treated with a serial dilution of DALTAC-1, Neg-1, or Neg-2 compounds for 3 hours. Following treatment, cells were lysed in 1× AlphaLISA SureFire Ultra Lysis Buffer (PerkinElmer) for 30 minutes at 4 °C with gentle mixing.

For detection, high-binding MaxiSorp 96-well plates (Thermo Fisher Scientific) were coated overnight at 4 °C with 50 μL of anti-AR antibody (clone AR441, Thermo Fisher Scientific) diluted 1:50 in PBS. After washing with TBST and blocking with 5% BSA for 2 hours at room temperature, 50 μL of cell lysate was added to each well and incubated for 1 hour at room temperature. Plates were washed three times with TBST and then incubated overnight at 4 °C with rabbit anti-p300 (D8Z4E, #86377, Cell Signaling Technology) antibody diluted 1:5000 in blocking buffer.

After washing, HRP-conjugated goat anti-rabbit secondary antibody (Invitrogen, #32260; 1:100,000 dilution) was added and incubated for 1 hour at room temperature. Plates were washed 7–8 times with TBST, and chemifluorescent signals were developed using QuantaRed^™^ Enhanced Chemifluorescent HRP Substrate (Thermo Fisher Scientific). Fluorescence (Ex = 570 nm, Em = 585 nm) was immediately measured using a Tecan Infinite M1000 Pro plate reader, with readings taken at 5 and 15 minutes.

### Nuclear immunoprecipitation

Cells (50 million) were washed once with ice-cold phosphate-buffered saline (PBS), scraped, and pelleted at 500 g for 5 minutes at 4 °C. The cell pellet was resuspended in 2 mL hypotonic buffer (20 mM HEPES-KOH pH 7.5, 10 mM KCl, 1 mM MgCl_2_, 0.1% Triton X-100, 15 mM β-mercaptoethanol) supplemented with protease and phosphatase inhibitors, and rotated for 1 hour at 4 °C. Nuclei were collected by centrifugation at 2,000 g for 5 minutes at 4 °C, the supernatant (cytoplasmic fraction) was discarded, and nuclei were washed once with hypotonic buffer.

The nuclear pellet was lysed in 1 mL nuclear lysis buffer (20 mM HEPES-KOH pH 7.5, 150 mM NaCl, 10 mM KCl, 1 mM MgCl_2_, 0.5% Triton X-100, 15 mM β-mercaptoethanol) containing protease and phosphatase inhibitors, and rotated for 1 hour at 4 °C. Benzonase^®^ (500 U mL⁻¹; Sigma-Aldrich #E1014) was added during lysis to digest nucleic acids. Lysates were clarified by centrifugation at 16,000 g for 20 minutes at 4 °C, and the supernatant was collected and precleared with 50 μL Dynabeads^™^ Protein G magnetic beads (Thermo Fisher #10004D). Protein concentration was determined using the Pierce^™^ 660 nm Protein Assay (Thermo Fisher #22660).

For each immunoprecipitation, 400 μg of precleared nuclear lysate was incubated overnight at 4 °C with either anti-androgen receptor antibody (Abcam #ab108341) or anti-p300 antibody (Active Motif #61401). Antigen–antibody complexes were captured with 30 μL Dynabeads^™^ Protein G for 2 hours at 4 °C. Beads were washed three times with wash buffer (20 mM HEPES-KOH pH 7.5, 150 mM NaCl, 10 mM KCl, 1 mM MgCl_2_).

For immunoblotting, bound proteins were eluted in 1× NuPAGE^™^ LDS Sample Buffer (Thermo Fisher #NP0007) supplemented with 50 mM DTT and subjected to SDS–PAGE. For mass spectrometry, washed beads were flash-frozen and stored at −80 °C for subsequent onbead trypsin digestion.

### RNA extraction and quantitative polymerase chain reaction

Total RNA was isolated using QIAzol^™^ Lysis Reagent (QIAGEN). Briefly, 1 × 10 cells were lysed in 700 μL QIAzol and incubated for 5 minutes at room temperature to allow for complete dissociation of nucleoprotein complexes. Following the addition of 140 μL chloroform, samples were vigorously mixed and centrifuged at 12,000 × g for 15 minutes at 4 °C. The aqueous phase was collected, mixed with 1.5 volumes of ethanol, and applied to a RNeasy Mini spin column (QIAGEN) for purification according to the miRNeasy Mini Kit protocol. RNA was eluted in RNase-free water, and the concentration and purity were assessed using a NanoDrop spectrophotometer (Thermo Fisher Scientific).

For quantitative PCR analysis, cDNA was synthesized from purified RNA using the Maxima^™^ First Strand cDNA Synthesis Kit (Thermo Fisher Scientific) according to the manufacturer’s instructions. qPCR reactions were performed with SYBR^™^ Green PCR Master Mix (Applied Biosystems) on a QuantStudio^™^ 7 Real-Time PCR System (Applied Biosystems). Primers used in this study are listed below: *GAPDH* forward (F): ACAACTTTGGTATCGTGGAAGG and reverse (R): GCCATCACGCCACAGTTTC; *KLK3/PSA* F: GACCAAGTTCATGCTGTGTGC and R: CCACTCACCTTTCCCCTCAAG; *TMPRSS2* F: GTCCCCACTGTCTACGAGGT and R: CAGACGACGGGGTTGGAAG; *NKX3–1* F: CCCACACTCAGGTGATCGAG and R: GAGCTGCTTTCGCTTAGTCTT; *MYC* F: ATGGCCCATTACAAAGCCG and R: TTTCTGGAGTAGCAGCTCCTAA; *CCND1* F: GCTGCGAAGTGGAAACCATC and R: CCTCCTTCTGCACACATTTGAA.

### Acetyl (lysine)-proteomics analysis

VCaP cells were treated with DMSO, 100 nM DALTAC-1 for 4 or 12 hours, or a combination of p300/CBPi and ARi for 12 hours, each in three biological replicates. The cells were then subjected to acetyl-lysine proteomics analysis as previously described^17^. In brief, total proteins were extracted, digested with trypsin, and acetylated peptides were enriched using anti–acetyl-lysine antibody–conjugated beads (Cell Signaling Technology). Enriched peptides were analyzed by nanoLC-MS/MS on a Q Exactive^™^ HF mass spectrometer (Thermo Fisher Scientific), and raw data were processed using MaxQuant (v2.3.0.0) with acetyl-lysine and protein N-terminal acetylation set as variable modifications.

#### Protein Identification by LC–tandem mass spectrometry In-solution digestion:

Co-IP assays were performed as described above. After the final wash, magnetic beads were resuspended in 50 μl of 0.1 M ammonium bicarbonate buffer (pH ~8). Cysteines were reduced by adding 50 μl of 10 mM DTT and incubating at 45 °C for 30 minutes. Samples were cooled to room temperature and alkylated with 65 mM 2-chloroacetamide for 30 minutes in the dark. Proteins were digested overnight (~16 hours) with 1 μg of sequencing-grade modified trypsin at 37 °C with constant shaking in a ThermoMixer. Digestion was quenched by acidification, and peptides were desalted using SepPak C18 cartridges (Waters) according to the manufacturer’s instructions. Eluates were dried completely in a vacuum concentrator and resuspended in 8 μl of 0.1% formic acid/2% acetonitrile. Two microliters of each sample were injected for LC–MS/MS analysis.

#### LC–MS/MS analysis:

Peptides were separated on a nano-capillary reverse-phase column (Acclaim PepMap C18, 2 μm, 50 cm; Thermo Scientific) using a linear gradient of 0.1% formic acid/2% acetonitrile (Buffer A) and 0.1% formic acid/95% acetonitrile (Buffer B) at a flow rate of 300 nl/minute over 180 minutes (2–22% B in 110 minutes; 22–40% B in 25 minutes; 40–90% B in 5 minutes; hold at 90% B for 5 minutes; equilibrate in Buffer A for 25 minutes). Eluting peptides were analyzed on an Orbitrap Fusion Tribrid mass spectrometer (Thermo Scientific) equipped with an EasySpray source. MS1 scans were acquired at 120,000 resolution (AGC target 1×10 ; maximum injection time 50 ms). Data-dependent MS/MS acquisition was performed in topspeed mode (3 s cycle) using collision-induced dissociation (NCE 32%; AGC target 1×10; maximum injection time 45 ms).

#### Protein identification:

Proteins were identified by searching MS/MS spectra against the Homo sapiens UniProtKB/Swiss-Prot database (UniProt release 2025_05; ~20,500 reviewed entries; downloaded May 2025) using Proteome Discoverer v3 (Thermo Scientific). Search parameters included a precursor mass tolerance of 10 ppm and fragment ion tolerance of 0.2 Da. Up to two missed cleavages were allowed. Carbamidomethylation of cysteine was set as a fixed modification, and oxidation of methionine and deamidation of asparagine/glutamine were included as variable modifications. Peptide-spectrum matches were filtered using Percolator, and proteins and peptides with a false discovery rate (FDR) ≤1% were retained for downstream analysis.

### RNA-seq and data analysis

VCaP cells were treated with 100 nM DALTAC-1 or a combination of 100 nM p300/CBPip300/CBPi and ARi for 4, 12, or 24 hours, each in two biological replicates. The cells were then subjected to RNA sequencing as previously described^17^. In brief, total RNA was extracted, and ribosomal RNA was removed using the RiboErase module of the KAPA RNA HyperPrep Kit (Roche). rRNA-depleted RNA was used for library preparation following the manufacturer’s protocol, and libraries were quality-checked on an Agilent 2100 Bioanalyzer before sequencing on an Illumina HiSeq 2500 platform (paired-end, 2 × 100 bp). Sequencing reads were quantified with Kallisto (v0.46.1), normalized with edgeR (v3.39.6), and analyzed for differential expression using limma-voom (v3.53.10). Gene set enrichment analysis was performed using fgsea (v1.24.0), and data visualization employed tidyverse, gplots, ggplot2, and EnhancedVolcano (v1.15.0) packages in R.

### ChIP-seq and data analysis

Chromatin immunoprecipitation followed by sequencing (ChIP-seq) was performed as previously described^17^. In brief, cells were crosslinked in 1% formaldehyde, quenched with glycine, and lysed for chromatin extraction. Chromatin was sheared to 200–600 bp fragments using a Bioruptor (Diagenode) and subjected to immunoprecipitation with antibodies against transcription factors (AR, FOXA1, p300, ERG, and BRD4) or histone marks (H3K27ac, H2BK20ac, and RNA Pol II) using the iDeal ChIP-seq Kit (Diagenode). Drosophila spike-in chromatin (Active Motif, 53083) and spike-in antibody (Active Motif, 61686) were added during the overnight incubation. After reverse crosslinking and DNA purification, sequencing libraries were prepared and sequenced on an Illumina HiSeq 2500 platform.

ChIP-seq reads were trimmed using Trimmomatic (v0.39). Alignment was then performed separately to both the human genome (hg38) and the drosophila genome (dm6) using BWA (v0.7.17). Low-quality and duplicate reads were filtered with SAMtools and Picard, respectively. Normalization ratios were calculated based on the lowest amount of uniquely aligned reads per ChIP target to each genome as recommended on the vendor’s website. Reads were then downsampled per the normalization ratios. Peak calling was then performed with MACS2 using narrow or broad settings depending on the target, and blacklisted genomic regions were removed using BEDTools with the ENCODE exclusion list. Processed data were converted to BigWig format using UCSC wigToBigWig for visualization.

### Enrichment heatmaps and profile plots

Read density heatmaps and average enrichment profiles were generated using deepTools. For histone marks, signals were plotted within ±2.5 kb of the reference point, and for transcriptome factors within ±1 kb. The parameters --skipZeros, --averageType mean, and --plotType se were applied. ENCODE blacklist regions (ENCFF356LFX) were excluded from analysis. Non-promoter regions were defined using the ChIPseeker R package tool annotatePeak excluding ±1 kb windows surrounding annotated gene TSS regions.

### Prostate organoid culture

Mouse prostate organoids and patient-derived xenograft organoids were established and maintained following previously described protocols^52,90^. Briefly, organoids were cultured in 50 μL Matrigel domes and overlaid with prostate organoid medium, with media changed every 2–3 days and passaging performed weekly. For growth and drug-response assays, organoid fragments were dissociated into single cells and used to generate new organoids (1,000 cells per dome for standard viability assays) in complete organoid medium. Cell viability was measured using the CellTiter-Glo 3D assay (Promega) according to the manufacturer’s instructions.

### Human prostate tumor xenograft models

Male SCID mice (6–8 weeks old) were obtained from the breeding colony at the University of Michigan and maintained under specific pathogen–free conditions. All animal procedures were approved by the University of Michigan Institutional Animal Care and Use Committee (IACUC). Subcutaneous xenograft tumors were established by injecting 3 × 10 VCaP cells suspended in serum-free medium mixed 1:1 with Matrigel (BD Biosciences) into both dorsal flanks. Tumor growth was monitored weekly using digital calipers, and volumes were calculated as (π/6) × (length × width^2^). At study endpoints, mice were euthanized, and tumors were excised and weighed.

The castration-resistant VCaP model was generated as previously described^17^. Briefly, mice were castrated once tumors reached 150–200 mm^3^, and treatment with DALTAC-1 (30 mg/kg i.p. or 60 mg/kg p.o.), combination of p300/CBPi (30 mg/kg p.o.) and ARi (15 mg/kg p.o.) or vehicle was initiated after tumor regrowth to baseline size for 24 days.

### Tissue microarray generation

A tissue microarray (TMA) was created according to a design involving 8 unique cases from 4 different subgroups: vehicle (n=2), p300/CBPi+ARi (n=2), DALTAC-1 ip (n=2), and DALTAC po (n=2). Two independent samples were selected from each subgroup. To ensure data reliability and representativeness, a replicate core from each sample was included in the TMA, resulting in a focused 16-core TMA. Technical details are as follows: Briefly, FFPE donor blocks for the selected samples were chosen by two study pathologists (RM and SM) after reviewing the hematoxylin & eosin (H&E) staining. The pathologists then annotated the H&E slides to identify the most representative tumor area of interest (AOI) on the surface of each donor block. The Semiautomatic tissue arrayer from Beecher Instruments (Sun Prairie, Wisconsin, United States) was used to punch 1 mm-diameter cores from each marked region. These cores were carefully transferred into a recipient acrylic block in a grid-like pattern. Once transferred, the recipient block was incubated at 37 degrees Celsius for 30 minutes to ensure proper fusion of the cores within the paraffin. The prepared TMA block was then sectioned at 4-micron thickness and stained with H&E to assess the quality of the final TMA.

### Sequential multiplex immunofluorescence (Seq-mIF) by COMET analysis

Four-micron-thick sections from the PDX TMA, mounted on positively charged slides, were cut and prepared according to the guidelines and recommendations. Subsequently, the slides were deparaffinized using a standard xylene and graded ethanol series, followed by heat-induced epitope retrieval (HIER) as per the Lunaphore COMET protocol to unmask target epitopes^61^. Automated sequential immunofluorescence (seqIF^™^) staining was performed on the COMET^™^ instrument (Lunaphore) to enable highly multiplexed protein detection with the target primary antibodies. This process involved cycles of staining, imaging, and elution managed by the COMET Control software through controlled temperature and microfluidic reagent delivery. Each cycle included incubation with unconjugated primary antibodies, followed by species-specific fluorescently conjugated secondary antibodies (e.g., Alexa Fluor 555 and 647), automated image acquisition including DAPI counterstaining, and complete antibody removal via elution. After the final staining cycle, the COMET Control software automatically stitched, aligned, and stacked images to produce a single OME-TIFF file for each sample. During post-processing, autofluorescence background was systematically subtracted using reference images acquired from unstained tissue sections at the beginning of the protocol.

### Statistics used for Seq-mIF

The raw prostate TMA data included single-cell measurements across multiple markers along with their spatial locations. Markers were grouped into two panels based on biological relevance, as shown in the plots. To normalize marker expression and reduce skewness, a log1p transformation (log1p(Expression)) was applied. Outliers were identified and removed separately for each marker within each group. Outliers were defined as values exceeding ±5 median absolute deviations (MAD) from the group-specific median. The Median Absolute Deviation (MAD) is a robust measure of statistical dispersion that calculates the median of the absolute deviations from the median, making it less sensitive to extreme outliers than standard deviation. This approach preserves most of the data while minimizing the impact of extreme values.

### Prostate patient-derived xenograft (PDX) models

Prostate cancer PDX models MDA-PCa-146–12 and MDA-PCa-183 were established and maintained as previously described^17^. Briefly, tumor fragments (~2 mm^3^) were implanted subcutaneously into male SCID mice and propagated through serial passage. Once tumors reached approximately 200 mm^3^, mice were randomized into treatment groups and administered vehicle or DALTAC-1 (30 mg/kg, i.p.) three times per week. Tumor volumes were measured weekly, and all procedures were conducted in accordance with protocols approved by the University of Michigan Institutional Animal Care and Use Committee (IACUC).

### Statistics and reproducibility

All experimental procedures and statistical analyses were performed as previously described^17^. In brief, key experiments were independently repeated at least three times, with most quantitative assays carried out in biological replicates as noted. Representative images and immunoblots reflect reproducible results, and the number of biological replicates is provided in the figure legends. No statistical method was used to predetermine sample size, no data were excluded, and experiments were neither randomized nor blinded. Two-sided statistical tests were used unless otherwise stated. Comparisons between two groups used unpaired t-tests or Wilcoxon rank-sum tests, and multiple comparisons were corrected using the Benjamini–Hochberg method. Exact p-values are reported in the figure legends.

## Extended Data

## Supplementary Material

Supplement 1

## Data Availability

All data are available in the manuscript and the supplementary information. Raw sequencing data have been deposited in the Gene Expression Omnibus (GEO) with the accession numbers GSE310948 and GSE310949.

## Code availability

No custom code or software were used in this study. Details of all code used in the study are provided in the Methods section.
