# Supplementary material for: Rewiring Oncogenic Transcriptional Complexes with Domain-ALTeration Chimeras (DALTACs) in Prostate Cancer
Source: bioRxiv. 2026 Apr 28:2026.04.24.720638. Preprint. [Version 1] doi: 10.64898/2026.04.24.720638 (PMC13142405; doi:10.64898/2026.04.24.720638)
Supplement: Supplement 1 [file media-1.docx]

**Supplementary Notes**

**Synthesis of Compounds 1-7, DALTAC-1 (JZY3032), Neg-1, Neg-2, JZY3222, and ARi (JZY3221)**

- Compound synthesis general information
- Scheme 1 Synthetic route of **Compounds 1-6**
- Scheme 2 Synthetic route of **Compound** **7** and **DALTAC-1 (JZY3032)**
- Scheme 3 Synthetic route of **Neg-1**
- Scheme 4 Synthetic route of **Neg-2**
- Scheme 5 Synthetic route of **JZY3222, ARi (JZY3221)**
- The ^1^H NMR, ^13^C NMR, and LC-MS traces of **DALTAC-1 (JZY3032)**
- The ^1^H NMR traces of Compounds **1-7**, **Neg-1, Neg-2, JZY3222**, and **ARi (JZY3221)**

**Compound synthesis general information**

All commercially available reagents and solvents were used without further purification. All chemical reactions were monitored by thin-layer chromatography (TLC) plates with visualization under UV light (254 or 365 nm). ^1^H NMR spectra were performed with Bruker AV-400 spectrometer. ^13^C NMR spectra were recorded on Bruker AV-400 spectrometer at 101 MHz; internal reference was either TMS or deuterated NMR solvent. In reported spectral data, the format (δ) chemical shift (multiplicity, *J* values in Hz, integration) was used with the following abbreviations: s = singlet, d = doublet, t = triplet, q = quartet, hept = heptet, dd = doublet of doublets, and m = multiplet. Low-resolution mass spectrometric (MS) analysis was carried out with a Waters UPLC ACQUITY QDa mass spectrometer. Flash column chromatography was performed by Teledyne CombiFlash RF+ using RediSep Rf silica gel flash column. The final compounds were all purified by a C18 reverse phase preparative HPLC column (SunFire Prep C18 OBD 5 μm, 50 mm × 100 mm) with solvent A (0.1% TFA in H_2_O) and solvent B (0.1% TFA in MeCN) as eluents at 60 mL/min flow rate. The purity of all the final compounds was measured and confirmed to be >95% by rapid ultra-performance liquid chromatography mass spectrometry (UPLCMS) analysis (10-100% MeCN in H_2_O containing 0.1% formic acid in 5 min, 1.0 mL/min flow rate) with a C18 column (ACQUITY UPLC BEH C18 1.7 μm, 2.1 mm × 50 mm).

**Scheme 1: Synthesis of Compounds 1-6.**

***Reagents and conditions*:** (a) HATU, DMF, Et_3_N, rt, 15 min; (b) K_2_CO_3_, DMF, 65 ^o^C, 3 h. (c) Et_3_N, Sodium triacetoxyborohydride, DCM, rt, 3 h; (d) DCM, TFA, 40 ^o^C, 3 h; (e) K_2_CO_3_, DMF, 65 ^o^C, 3 h.

Intermediate **11** was prepared according to the reported procedures.^1^

***6-chloro-N-((1r,4r)-4-(3-chloro-4-cyanophenoxy)cyclohexyl)pyridazine-3-carboxamide (10)***

Step a: 6-chloropyridazine-3-carboxylic acid **9** (5.0 g, 31.54 mmol) was dissolved in DMF (100 mL), HATU (12.0 g, 31.54) and Et_3_N (4.8 g, 47.31 mmol) was added. The mixture was stirred at room temperature for 2 min, then intermediate **8** (7.9 g, 31.54 mmol) was added in to the mixture. The resulting mixture was stirred at room temperature for an additional 13 min. After that, the mixture was diluted with water and extracted with DCM. The organic phases were combined and concentrated under reduced pressure. The residue was purified by flash column chromatography (0–50% EtOAc/*n*-hexane) to give intermediate **10** (5.5 g, yield: 45%) as yellow solid. ^1^H NMR (400 MHz, DMSO-*d*_6_) δ 9.17 (d, *J* = 8.1 Hz, 1H), 8.23 (d, *J* = 8.8 Hz, 1H), 8.11 (d, *J* = 8.8 Hz, 1H), 7.86 (d, *J* = 8.8 Hz, 1H), 7.40 (d, *J* = 2.4 Hz, 1H), 7.15 (dd, *J* = 8.8, 2.4 Hz, 1H), 4.54 (tt, *J* = 10.4, 4.2 Hz, 1H), 3.92 (tdq, *J* = 11.7, 8.0, 3.9 Hz, 1H), 2.12 (d, *J* = 13.8 Hz, 2H), 1.90 (d, *J* = 14.2 Hz, 2H), 1.71 (q, *J* = 13.2 Hz, 2H), 1.52 (q, *J* = 12.9 Hz, 2H). LC-MS *m*/*z* [M+H]^+^ 391.11.

***6-(4-(5-acetyl-3-(7-(difluoromethyl)-6-(1-methyl-1H-pyrazol-4-yl)-3,4-dihydroquinolin-1(2H)-yl)-4,5,6,7-tetrahydro-1H-pyrazolo[4,3-c]pyridin-1-yl)piperidin-1-yl)-N-((1r,4r)-4-(3-chloro-4-cyanophenoxy)cyclohexyl)pyridazine-3-carboxamide (1)***

Step b: To a solution of intermediate **10** (46.9 mg, 0.12 mmol) and **11** (51.0 mg, 0.1 mmol) in DMF (5 mL), was added K_2_CO_3_ (27.6 mg, 0.2 mmol). The mixture was stirred at 65 ^o^C for 3 h. Monitor the reaction through LC/MS until the intermediate **11** was completely consumed, and then the mixture was concentrated under reduced pressure. The crude residue was purified by pre-HPLC (25−100% MeCN/H_2_O in 70 min, the desired product started eluting at ~ 46% MeCN) to give the compound **1** (64.8 mg, yield: 75%) as yellow solid. ^1^H NMR (400 MHz, DMSO-*d*_6_) δ 8.63 (d, *J* = 8.2 Hz, 1H), 7.89 – 7.81 (m, 2H), 7.75 (s, 1H), 7.49 (s, 1H), 7.46 (d, *J* = 9.9 Hz, 1H), 7.40 (d, *J* = 2.4 Hz, 1H), 7.14 (dd, *J* = 8.7, 2.5 Hz, 1H), 7.09 (s, 1H), 6.78 (td, *J* = 51.6, 6.8 Hz, 1H), 6.77 (s,1H), 4.61 (d, *J* = 13.1 Hz, 2H), 4.57 – 4.44 (m, 2H), 4.39 – 4.22 (m, 1H), 4.15 (d, *J* = 20.4 Hz, 2H), 3.86 (s, 3H), 3.80 – 3.69 (m, 2H), 3.55 (q, *J* = 5.8 Hz, 2H), 3.24 (t, *J* = 25.7 Hz, 2H), 2.95 – 2.74 (m, 4H), 2.17 – 2.04 (m, 4H), 2.04 – 1.81 (m, 9H), 1.64 (q, *J* = 12.3 Hz, 2H), 1.51 (q, *J* = 11.7 Hz, 2H). LC-MS *m*/*z* [M+H]^+^ 864.36.

***1-(1-(1-(azetidin-3-yl)piperidin-4-yl)-3-(7-(difluoromethyl)-6-(1-methyl-1H-pyrazol-4-yl)-3,4-dihydroquinolin-1(2H)-yl)-1,4,6,7-tetrahydro-5H-pyrazolo[4,3-c]pyridin-5-yl)ethan-1-one (13a)***

Step c: To a solution of intermediate **11** (51.0 mg, 0.1 mmol) and **12a** (34.2 mg, 0.2 mmol) in DCM at room temperature, was added Et_3_N (20.2 mg, 0.2 mmol). The mixture was stirred at room temperature for 30 min. Then sodium triacetoxyborohydride (31.8 mg, 0.15 mmol) was added to this reaction, and the mixture was stirred at room temperature for 3 h. Monitor the reaction through LC/MS until the intermediate **11** was completely consumed, and then the mixture was concentrated under reduced pressure. The crude residue was purified by pre-HPLC (25−100% MeCN/H_2_O in 70 min, the desired product started eluting at ~ 38% MeCN).

Step d: The product obtained above was dissolved in 6 mL of DCM : TFA (5:1) and stirred at 40 ^o^C for an additional 3 h. Monitor the reaction through LC/MS until the reaction was completed. Then the mixture was concentrated and purified by pre-HPLC (10−100% MeCN/H_2_O in 90 min the desired product started eluting at ~ 20% MeCN), to give intermediate **13a** (34 mg, yield: 60%, two steps) as a clear, gel-like solid. ^1^H NMR (400 MHz, DMSO-*d*_6_) δ 7.77 (s, 1H), 7.51 (d, *J* = 0.8 Hz, 1H), 7.13 (s, 1H), 6.80 (td, *J* = 55.2, 6.8 Hz, 1H), 6.79 (d, *J* = 3.0 Hz, 1H), 4.44 – 4.24 (m, 3H), 4.23 – 4.03 (m, 5H), 3.87 (s, 3H), 3.79 – 3.67 (m, 2H), 3.59 (t, *J* = 6.0 Hz, 2H), 3.51 – 3.36 (m ,2H), 3.03 – 2.79 (m, 5H), 2.76 (s, 1H), 2.28 – 2.02 (m, 6H), 2.01 – 1.92 (m, 3H). LC-MS *m*/*z* [M+H]^+^ 565.35.

***1-(1-([1,4'-bipiperidin]-4-yl)-3-(7-(difluoromethyl)-6-(1-methyl-1H-pyrazol-4-yl)-3,4-dihydroquinolin-1(2H)-yl)-1,4,6,7-tetrahydro-5H-pyrazolo[4,3-c]pyridin-5-yl)ethan-1-one (13b)***

Compound **13b** was synthesized from intermediates **11** and **12b** following a similar procedure to that of intermediate **13a**. ^1^H NMR (400 MHz, DMSO-*d*_6_) δ 7.77 (s, 1H), 7.51 (s, 1H), 7.13 (s, 1H), 6.81 (td, *J* = 55.2, 7.0 Hz, 1H), 6.77 (d, *J* = 3.0 Hz, 1H), 4.51 – 4.39 (s, 1H), 4.14 (d, *J* = 23.0 Hz, 2H), 3.87 (s, 3H), 3.80 – 3.69 (m, 2H), 3.67 – 3.53 m, 4H), 3.53 – 3.37 (m, 6H), 3.22 (q, *J* = 11.0 Hz, 2H), 2.95 (q, *J* = 11.9 Hz, 2H), 2.89 – 2.81 (d, *J* = 5.8 Hz, 3H), 2.79 – 2.72 (m, 1H) , 2.37 – 2.13 (m, 6H), 2.08 (d, *J* = 2.5 Hz, 2H), 1.91 – 1.78 (m, 2H). LC-MS *m*/*z* [M+H]^+^ 593.41.

***1-(1-(1-(azetidin-3-ylmethyl)piperidin-4-yl)-3-(7-(difluoromethyl)-6-(1-methyl-1H-pyrazol-4-yl)-3,4-dihydroquinolin-1(2H)-yl)-1,4,6,7-tetrahydro-5H-pyrazolo[4,3-c]pyridin-5-yl)ethan-1-one (13c)***

Compound **13c** was synthesized from intermediates **11** and **12c** following a similar procedure to that of intermediate **13a**. ^1^H NMR (400 MHz, DMSO-*d*_6_) δ 7.76 (s, 1H), 7.51 (s, 1H), 7.13 (s, 1H), 6.80 (td, *J* = 55.2, 6.8 Hz, 1H), 6.77 (d, *J* = 3.8 Hz, 1H), 4.44 – 4.33 (m, 1H), 4.14 (d, *J* = 22.3 Hz, 2H), 4.09 – 3.97 (m, 2H), 3.94 – 3.80 (s, 5H), 3.81 – 3.67 (m, 2H), 3.66 – 3.49 (m, 4H), 3.41 (s, 2H), 3.36 – 3.26 (m, 1H), 3.12 (s, 2H), 2.92 – 2.79 (m, 3H), 2.74 (t, *J* = 6.9 Hz, 1H), 2.32 – 2.17 (m, 2H), 2.17 – 2.05 (m, 4H), 2.04 – 1.90 (m, 3H).

***1-(1-(1-(3-azaspiro[5.5]undecan-9-yl)piperidin-4-yl)-3-(7-(difluoromethyl)-6-(1-methyl-1H-pyrazol-4-yl)-3,4-dihydroquinolin-1(2H)-yl)-1,4,6,7-tetrahydro-5H-pyrazolo[4,3-c]pyridin-5-yl)ethan-1-one (13d)***

Compound **13d** was synthesized from intermediates **11** and **12d** following a similar procedure to that of intermediate **13a**. ^1^H NMR (400 MHz, CD_3_OD) δ 7.65 (s, 1H), 7.52 (s, 1H), 7.11 (d, *J* = 8.5 Hz, 1H), 6.86 – 6.37 (m, 2H), 4.68 – 4.40 (m, 1H), 4.25 (dd, *J* = 23.5, 7.2 Hz, 2H), 3.93 (d, *J* = 1.0 Hz, 3H), 3.86 (dt, *J* = 25.9, 5.8 Hz, 2H), 3.73 – 3.62 (m, 4H), 3.49 – 3.23 (m, 3H), 3.23 – 3.10 (m, 4H), 2.92 (d, *J* = 13.3 Hz, 3H), 2.87 – 2.78 (m, 1H), 2.52 – 2.15 (m, 6H), 2.12 – 1.90 (m, 7H), 1.88 – 1.66 (m, 4H), 1.65 – 1.54 (m, 2H), 1.46 – 1.27 (m, 2H). LC-MS *m*/*z* [M+H]^+^ 661.29

***1-(1-(1-((3-azaspiro[5.5]undecan-9-yl)methyl)piperidin-4-yl)-3-(7-(difluoromethyl)-6-(1-methyl-1H-pyrazol-4-yl)-3,4-dihydroquinolin-1(2H)-yl)-1,4,6,7-tetrahydro-5H-pyrazolo[4,3-c]pyridin-5-yl)ethan-1-one (13e)***

Compound **13e** was synthesized from intermediates **11** and **12d** following a similar procedure to that of compound **13a**.^1^H NMR (400 MHz, CD_3_OD) δ 7.65 (s, 1H), 7.51 (d, *J* = 0.8 Hz, 1H), 7.12 (d, *J* = 8.6 Hz, 1H), 6.88 – 6.35 (m, 2H), 4.66 – 4.38 (m, 1H), 4.24 (dd, *J* = 20.8, 7.3 Hz, 2H), 3.93 (d, *J* = 1.3 Hz, 3H), 3.86 (dt, *J* = 25.7, 5.8 Hz, 2H), 3.80 – 3.61 (m, 4H), 3.26 – 3.04 (m, 8H), 2.99 – 2.79 (m, 4H), 2.56 – 2.15 (m, 6H), 2.13 – 2.00 (m, 3H), 1.96 – 1.65 (m, 7H), 1.64 – 1.54 (m, 2H), 1.39 – 1.19 (m, 4H). LC-MS *m*/*z* [M+H]^+^ 675.33.

***6-(3-(4-(5-acetyl-3-(7-(difluoromethyl)-6-(1-methyl-1H-pyrazol-4-yl)-3,4-dihydroquinolin-1(2H)-yl)-4,5,6,7-tetrahydro-1H-pyrazolo[4,3-c]pyridin-1-yl)piperidin-1-yl)azetidin-1-yl)-N-((1r,4r)-4-(3-chloro-4-cyanophenoxy)cyclohexyl)pyridazine-3-carboxamide (2)***

Step e: To a solution of intermediate **13a** (28.3 mg, 0.05 mmol) and **10** (23.4 mg, 0.06 mmol) in DMF (5 mL), was added K_2_CO_3_ (13.8 mg, 0.1 mmol). The mixture was stirred at 65 ^o^C for 3 h. Monitor the reaction through LC/MS until the intermediate **13a** was completely consumed, and then the mixture was concentrated under reduced pressure. The crude residue was purified by pre-HPLC (25−100% MeCN/H_2_O in 70 min, the desired product started eluting at ~ 40% MeCN) to give the compound **2** (36.5 mg, yield: 80%) as yellow solid. ^1^H NMR (400 MHz, DMSO-*d*_6_) δ 8.65 (d, *J* = 8.2 Hz, 1H), 7.95 (d, *J* = 9.2 Hz, 1H), 7.87 (d, *J* = 8.8 Hz, 1H), 7.76 (s, 1H), 7.50 (s, 1H), 7.39 (d, *J* = 2.4 Hz, 1H), 7.14 (dd, *J* = 8.8, 2.4 Hz, 2H), 7.00 (d, *J* = 9.2 Hz, 1H), 6.81 (td, *J* = 51.6, 6.8 Hz, 1H), 6.79 (d, *J* = 3.7 Hz, 1H), 4.61 – 4.25 (m, 7H), 4.15 (d, *J* = 21.6 Hz, 2H), 3.93 – 3.80 (m, 4H), 3.81 – 3.40 (m, 6H), 3.23 – 3.05 (m, 2H), 2.95 – 2.73 (m, 4H), 2.34 – 2.15 (m, 4H), 2.15 – 2.05 (m, 4H), 2.02 – 1.93 (m, 3H), 1.90 (d, *J* = 13.1 Hz, 2H), 1.65 (q, *J* = 12.2 Hz, 2H), 1.52 (q, *J* = 11.5 Hz, 2H). LC-MS *m*/*z* [M+H]^+^ 919.59.

***6-(4-(5-acetyl-3-(7-(difluoromethyl)-6-(1-methyl-1H-pyrazol-4-yl)-3,4-dihydroquinolin-1(2H)-yl)-4,5,6,7-tetrahydro-1H-pyrazolo[4,3-c]pyridin-1-yl)-[1,4'-bipiperidin]-1'-yl)-N-((1r,4r)-4-(3-chloro-4-cyanophenoxy)cyclohexyl)pyridazine-3-carboxamide (3)***

Compound **3** was synthesized from intermediates **13b** and **10** following a similar procedure to that of compound **2**. ^1^H NMR (400 MHz, DMSO-*d*_6_) δ 8.65 (d, *J* = 8.2 Hz, 1H), 7.88 (dd, *J* = 9.1, 5.8 Hz, 2H), 7.76 (s, 1H), 7.51 (s, 1H), 7.46 (d, *J* = 9.6 Hz, 1H), 7.40 (d, *J* = 2.4 Hz, 1H), 7.17 – 7.10 (m, 2H), 6.81 (td, *J* = 51.6, 7.2 Hz, 1H), 6.77 (d, *J* = 3.7 Hz, 1H), 4.68 (d, *J* = 13.1 Hz, 2H), 4.54 (q, *J* = 9.5, 7.6 Hz, 1H), 4.49 – 4.39 (m, 1H), 4.14 (d, *J* = 22.3 Hz, 2H), 3.93 – 3.81 (m, 4H), 3.80 – 3.68 (m, 2H), 3.59 (q, *J* = 6.2, 5.6 Hz, 5H), 3.21(q, *J* = 10.3 Hz, 2H), 3.04 (t, *J* = 12.7 Hz, 2H), 2.92 – 2.71 (m, 4H), 2.30 (d, *J* = 12.8 Hz, 2H), 2.23 – 2.05 (m, 8H), 2.05 – 1.84 (m, 5H), 1.65 (q, *J* = 12.3, 11.5 Hz, 4H), 1.52 (q, *J* = 11.6 Hz, 2H). LC-MS *m*/*z* [M+H]^+^ 947.35.

***6-(3-((4-(5-acetyl-3-(7-(difluoromethyl)-6-(1-methyl-1H-pyrazol-4-yl)-3,4-dihydroquinolin-1(2H)-yl)-4,5,6,7-tetrahydro-1H-pyrazolo[4,3-c]pyridin-1-yl)piperidin-1-yl)methyl)azetidin-1-yl)-N-((1r,4r)-4-(3-chloro-4-cyanophenoxy)cyclohexyl)pyridazine-3-carboxamide (4)***

Compound **4** was synthesized from intermediates **13c** and **10** following a similar procedure to that of compound **2**. ^1^H NMR (400 MHz, DMSO-*d*_6_) δ 8.59 (d, *J* = 7.4 Hz, 1H), 7.88 (dd, *J* = 9.1, 7.7 Hz, 2H), 7.77 (s, 1H), 7.51 (s, 1H), 7.39 (d, *J* = 2.4 Hz, 1H), 7.17 – 7.10 (m, 2H), 6.93 (d, *J* = 9.7 Hz, 1H), 6.82 (td, *J* = 51.6, 7.2 Hz, 1H), 6.82 – 6.77 (m, 1H), 4.59 – 4.49 (m, 1H), 4.45 – 4.36 (m, 1H), 4.32 (t, *J* = 8.5 Hz, 2H), 4.16 (d, *J* = 21.8 Hz, 2H), 3.97 (t, *J* = 7.3 Hz, 2H), 3.91 – 3.81 (m, 4H), 3.75 (d, *J* = 17.6 Hz, 2H), 3.67 – 3.56 (m, 4H), 3.56 – 3.41 (m, 2H), 3.39 – 3.27 (m,1H), 3.17 (q, *J* = 11.7 Hz, 2H), 2.92 – 2.73 (m, 4H), 2.30 (q, *J* = 13.5 Hz, 2H), 2.15 (d, *J* = 15.9 Hz, 3H), 2.09 (s, 3H), 2.02 – 1.93 (m, 2H), 1.90 (d, *J* = 12.1 Hz, 2H), 1.65 (q, *J* = 12.2 Hz, 2H), 1.52 (q, *J* = 12.1, 11.7 Hz, 2H). LC-MS *m*/*z* [M+H]^+^ 933.62.

***6-(9-(4-(5-acetyl-3-(7-(difluoromethyl)-6-(1-methyl-1H-pyrazol-4-yl)-3,4-dihydroquinolin-1(2H)-yl)-4,5,6,7-tetrahydro-1H-pyrazolo[4,3-c]pyridin-1-yl)piperidin-1-yl)-3-azaspiro[5.5]undecan-3-yl)-N-((1r,4r)-4-(3-chloro-4-cyanophenoxy)cyclohexyl)pyridazine-3-carboxamide (5)***

Compound **5** was synthesized from intermediates **13d** and **10** following a similar procedure to that of compound **2**. ^1^H NMR (400 MHz, DMSO-*d*_6_) δ 8.60 (d, *J* = 8.1 Hz, 1H), 7.87 (d, *J* = 8.8 Hz, 1H), 7.83 (d, *J* = 9.5 Hz, 1H), 7.77 (s, 1H), 7.51 (s, 1H), 7.40 (d, *J* = 2.4 Hz, 1H), 7.37 (d, *J* = 9.6 Hz, 1H), 7.14 (dd, *J* = 8.8, 2.4 Hz, 2H), 6.82 (td, *J* = 51.6, 7.2 Hz, 1H), 6.81 – 6.76 (m, 1H), 4.54 (p, *J* = 5.6 Hz, 1H), 4.49 – 4.39 (m, 1H), 4.15 (d, *J* = 21.8 Hz, 2H), 3.92 – 3.82 (m, 4H), 3.80 – 3.65 (m, 6H), 3.60 (q, *J* = 5.1 Hz, 4H), 3.30 – 3.13 (m, 3H), 2.92 – 2.72 (m, 4H), 2.32 (d, *J* = 14.4 Hz, 2H), 2.21 – 2.05 (m, 6H), 2.02 – 1.94 (m, 3H), 1.94 – 1.78 (m, 6H), 1.73 – 1.57 (m, 6H), 1.53 (t, *J* = 11.4 Hz, 2H), 1.41 (s, 2H), 1.29 – 1.13 (m, 2H). LC-MS *m*/*z* [M+H]^+^ 1015.31.

***6-(9-((4-(5-acetyl-3-(7-(difluoromethyl)-6-(1-methyl-1H-pyrazol-4-yl)-3,4-dihydroquinolin-1(2H)-yl)-4,5,6,7-tetrahydro-1H-pyrazolo[4,3-c]pyridin-1-yl)piperidin-1-yl)methyl)-3-azaspiro[5.5]undecan-3-yl)-N-((1r,4r)-4-(3-chloro-4-cyanophenoxy)cyclohexyl)pyridazine-3-carboxamide (6)***

Compound **6** was synthesized from intermediates **13e** and **10** following a similar procedure to that of compound **2**. ^1^H NMR (400 MHz, DMSO-*d*_6_) δ 8.58 (d, *J* = 8.2 Hz, 1H), 7.86 (d, *J* = 8.8 Hz, 1H), 7.83 (d, *J* = 9.6 Hz, 1H), 7.76 (s, 1H), 7.51 (s, 1H), 7.39 (d, *J* = 2.4 Hz, 1H), 7.36 (d, *J* = 9.7 Hz, 1H), 7.14 (dd, *J* = 8.8, 2.4 Hz, 2H), 6.81 (td, *J* = 51.6, 6.6 Hz, 1H), 6.78 (d, *J* = 2.6 Hz, 1H), 4.60 – 4.49 (m, 1H), 4.44 – 4.33 (s, 1H), 4.15 (d, *J* = 20.6 Hz, 2H), 3.92 – 3.80 (s, 4H), 3.79 – 3.39 (m, 10H), 3.20 – 3.04 (m, 2H), 3.01 (s, 2H), 2.93 – 2.72 (m, 4H), 2.36 (q, *J* = 14.4 Hz, 2H), 2.23 – 2.04 (m, 6H), 2.02 – 1.94 (m, 2H), 1.90 (d, *J* = 11.9 Hz, 2H), 1.85 – 1.70 (m, 3H), 1.71 – 1.45 (m, 8H), 1.41 (s, 2H), 1.27 – 1.10 (m, 4H). LC-MS *m*/*z* [M+H]^+^ 1029.27.

**Scheme 2: Synthesis of Compound 7 and DALTAC-1 (JZY3032).**

***Reagents and conditions*:** (a) HATU, DMF, Et_3_N, rt, 15 min; (b) DIPEA, DMF, 55 °C, 30 min; (c) Et_3_N, Sodium triacetoxyborohydride, DCM, rt, 3 h.

Intermediate **16** was prepared according to the reported procedures.^1^

Intermediate **17** was prepared according to the reported procedures.^2^

***N-((1r,4r)-4-((3-chloro-4-cyanophenyl)(methyl)amino)cyclohexyl)-6-(piperazin-1-yl)pyridazine-3-carboxamide (18)***

Step a: 6-chloropyridazine-3-carboxylic acid **9** (5.0 g, 31.54 mmol) was dissolved in DMF (100 mL), HATU (12.0 g, 31.54) and Et_3_N (4.8 g, 47.31 mmol) was added. The mixture was stirred at room temperature for 2 min, then intermediate **17** (8.32 g, 31.54 mmol) was added in to the mixture. The resulting mixture was stirred at room temperature for an additional 13 min; after that, the mixture was diluted with water and extracted with DCM. The organic phases were combined and concentrated under reduced pressure. The crude residue was used directly in the next step without further purification.

Step b: The residue obtained above was redissolved in DMF (80 mL), and then piperazine **14** (9.5 g, 110.39 mmol) was added. The mixture was stirred at 55 °C for 30 min. After that, all volatile materials were removed in a rotary evaporator and purified by silica gel column chromatography (0–20% MeOH/DCM) to afford intermediate **18** (8.6 g, yield: 60%, two steps).  ^1^H NMR (400 MHz, DMSO-*d*_6_) δ 8.59 (d, *J* = 8.3 Hz, 1H), 7.94 (d, *J* = 9.5 Hz, 1H), 7.62 (d, *J* = 9.0 Hz, 1H), 7.46 (d, *J* = 9.6 Hz, 1H), 6.95 (d, *J* = 2.5 Hz, 1H), 6.83 (dd, *J* = 9.1, 2.6 Hz, 1H), 3.96 (t, *J* = 5.3 Hz, 4H), 3.91 – 3.84 (m, 1H), 3.83 – 3.71 (m, 1H), 3.60 (q, *J* = 4.7 Hz, 1H), 3.23 (t, *J* = 5.3 Hz, 4H), 2.86 (s, 3H), 1.92 (d, *J* = 10.6 Hz, 2H), 1.82 – 1.63 (m, 6H).

***N-((1r,4r)-4-(3-chloro-4-cyanophenoxy)cyclohexyl)-6-(piperazin-1-yl)pyridazine-3-carboxamide (15)***

Intermediate **15** was synthesized with a similar procedure to that of intermediate **18**. ^1^H NMR (400 MHz, DMSO-*d*_6_) δ 8.68 (d, *J* = 8.3 Hz, 1H), 7.89 (dd, *J* = 17.4, 9.1 Hz, 2H), 7.43 (d, *J* = 9.6 Hz, 1H), 7.40 (d, *J* = 2.4 Hz, 1H), 7.14 (dd, *J* = 8.8, 2.5 Hz, 1H), 4.53 (td, *J* = 10.1, 5.0 Hz, 1H), 3.86 (t, *J* = 5.2 Hz, 4H), 3.74 (s, 1H), 3.21 – 3.09 (m, 3H), 2.18 – 2.06 (m, 2H), 1.95 – 1.85 (m, 2H), 1.72 – 1.59 (m, 2H), 1.59 – 1.45 (m, 2H).

***6-(4-(((1r,4r)-4-(5-acetyl-3-(7-(difluoromethyl)-6-(1-methyl-1H-pyrazol-4-yl)-3,4-dihydroquinolin-1(2H)-yl)-4,5,6,7-tetrahydro-1H-pyrazolo[4,3-c]pyridin-1-yl)cyclohexyl)methyl)piperazin-1-yl)-N-((1r,4r)-4-((3-chloro-4-cyanophenyl)(methyl)amino)cyclohexyl)pyridazine-3-carboxamide (DALTAC-1)***

Step c: To a solution of intermediate **16** (54 mg, 0.1 mmol) and **18** (59 mg, 0.13 mmol) in DCM at room temperature, was added Et_3_N (20.2 mg, 0.2 mmol). The mixture was stirred at room temperature for 30 min. Then sodium triacetoxyborohydride (31.8 mg, 0.15 mmol) was added to this reaction, and the mixture was stirred at room temperature for 3 h. Monitor the reaction through LC/MS until the intermediate **16** was completely consumed, and then the mixture was concentrated under reduced pressure. The crude residue was purified by pre-HPLC (20−100% MeCN/H_2_O in 60 min, the desired product started eluting at ~ 41% MeCN) to give **DALTAC-1** (63 mg, yield: 65%). ^1^H NMR (400 MHz, DMSO-*d*_6_) δ 8.50 (d, *J* = 8.3 Hz, 1H), 7.84 (d, *J* = 9.4 Hz, 1H), 7.76 (s, 1H), 7.61 (d, *J* = 9.0 Hz, 1H), 7.50 (d, *J* = 0.8 Hz, 1H), 7.35 (d, *J* = 9.7 Hz, 1H), 7.11 (s, 1H), 6.95 (d, *J* = 2.6 Hz, 1H), 6.83 (dd, *J* = 9.1, 2.5 Hz, 1H), 6.81 – 6.79 (m, 1H), 6.78 (td, *J* = 55.2, 6.8 Hz, 1H), 4.15 (d, *J* = 17.2 Hz, 2H), 4.07 – 3.96 (m, 1H), 3.87 (s, 3H), 3.86 – 3.64 (m, 8H), 3.63 – 3.55 (m, 2H), 2.92 – 2.78 (m, 7H), 2.74 (s, 1H), 2.48 (s, 2H), 2.19 (s, 2H), 2.08 (s, 2H), 2.03 – 1.87 (m, 10H), 1.84 (d, *J* = 11.8 Hz, 2H), 1.79 – 1.57 (m, 7H), 1.16 – 1.00 (m, 2H).^13^C NMR (101 MHz, DMSO-*d*_6_) δ 169.27, 162.80, 160.56, 153.46, 148.01, 145.27, 142.36, 138.27, 137.69, 137.12, 135.18, 131.09, 129.77, 128.88 (t, J = 20.6 Hz, 1C), 126.81 (d, J = 63.9 Hz, 1C), 121.05, 118.95, 118.21, 114.25 (t, J = 235.9 Hz, 1C), 113.10, 112.33, 111.32, 110.29, 106.12, 105.87, 96.35, 64.74, 57.52, 56.24 (2C), 53.18, 49.57, 47.75 (2C), 44.81, 43.31, 39.03 (2C), 38.46, 32.22, 31.59 (2C), 31.45 (2C), 30.36, 28.20 (2C), 27.38, 22.47, 22.17, 22.12, 21.85. LC-MS *m*/*z* [M+H]^+^ 974.30.

***6-(4-(((1r,4r)-4-(5-acetyl-3-(7-(difluoromethyl)-6-(1-methyl-1H-pyrazol-4-yl)-3,4-dihydroquinolin-1(2H)-yl)-4,5,6,7-tetrahydro-1H-pyrazolo[4,3-c]pyridin-1-yl)cyclohexyl)methyl)piperazin-1-yl)-N-((1r,4r)-4-(3-chloro-4-cyanophenoxy)cyclohexyl)pyridazine-3-carboxamide (7)***

Compound **7** was synthesized from intermediates **15** and **16** following a similar procedure to that of **DALTAC-1**. ^1^H NMR (400 MHz, DMSO-d6) δ 8.70 (d, J = 8.1 Hz, 1H), 7.96 (d, J = 9.5 Hz, 1H), 7.87 (d, J = 8.8 Hz, 1H), 7.76 (s, 1H), 7.52 (d, J = 8.5 Hz, 2H), 7.40 (d, J = 2.4 Hz, 1H), 7.14 (dd, J = 8.8, 2.5 Hz, 1H), 7.11 (s, 1H), 6.81(td, J = 54.8, 7.1 Hz, 1H), 6.79 (d, J = 2.4 Hz, 1H), 4.63 – 4.44 (m, 4H), 4.16 (d, J = 17.9 Hz, 2H), 4.10 – 4.00 (m, 1H), 3.94 – 3.80 (m, 4H), 3.78 – 3.64 (m, 4H), 3.58 (t, J = 5.7 Hz, 2H), 3.45 (t, J = 12.7 Hz, 2H), 3.24 – 2.97 (m, 4H), 2.89 – 2.72 (m, 4H), 2.09 (s, 4H), 2.01 – 1.84 (m, 11H), 1.66 (q, J = 12.2, 11.8 Hz, 2H), 1.52 (q, J = 11.3 Hz, 2H), 1.29 – 1.13 (m, 2H). LC-MS *m*/*z* [M+H]^+^ 961.63.

**Scheme 3: Synthesis of Neg-1.**

***^a^Reagents and conditions***: (a) 2-(Trimethylsilyl)ethoxymethyl chloride, NaH, DMF, 0 °C to rt, 3 h; (b) 1,4-Dioxane, Ruphos pd G2*,* Ruphos, Sodium *tert*-butoxide, 100 °C, N_2,_ overnight; (c) NBS, DCM, 0 °C, 30 min; (d) Pd(OAc)_2_, Pph_3_ ,K_2_CO_3_, 1,4-Dioxane : H_2_O (5:2), 100 °C, N_2_, 3 h; (e) [Formic acid](https://www.sigmaaldrich.com/US/en/substance/formicacid460364186), rt, 4 h; (f) Acetone, Sodium triacetoxyborohydride, DCM, rt, 30 min; (g) Tetrabutylammonium fluoride solution (1 M, in THF), THF, reflux, overnight; (h) Cs_2_CO_3_, DMF, 80 °C, overnight, (i) Diisobutylaluminium hydride (25% in toluene), DCM, −78 °C, 40% yield;  (j) Et_3_N, Sodium triacetoxyborohydride, DCM, rt, 2 h.

***tert-butyl 3-iodo-1-((2-(trimethylsilyl)ethoxy)methyl)-1,4,6,7-tetrahydro-5H-pyrazolo[4,3-c]pyridine-5-carboxylate (20)***

Step a: A solution of *tert*-butyl 3-iodo-1,4,6,7-tetrahydro-5*H*-pyrazolo[4,3-c]pyridine-5-carboxylate **19** (25.0 g, 71.6 mmol) in [anhydrous](https://www.sigmaaldrich.com/US/en/product/sial/227056) DMF (300 mL) at 0 °C, was added NaH (3.4 g, 85.9 mmol) in portions. After stirring at 0 °C for 30 min, 2-(Trimethylsilyl)ethoxymethyl chloride (14.3 g, 85.9 mmol) was slowly added into the mixture. After that, the mixture was warmed to room temperature and stirred for an additional 2.5 h. Monitor the reaction through LC/MS until the intermediate **19** was completely consumed, and then the mixture was quenched with ice water and extracted with EtOAc. The organic phases were combined and concentrated under reduced pressure. The residue was purified by flash column chromatography (0–60% EtOAc/*n*-hexane) to give intermediate **20** (27.5 g, yield: 80%) as white solid. ^1^H NMR (400 MHz, DMSO-*d*_6_) δ 5.35 (d, *J* = 16.3 Hz, 2H), 4.16 (d, *J* = 26.6 Hz, 2H), 3.61 (t, *J* = 5.8 Hz, 2H), 3.58 – 3.46 (m, 2H), 2.65 (dt, *J* = 37.0, 5.8 Hz, 2H), 1.42 (d, *J* = 1.3 Hz, 9H), 0.89 – 0.75 (m, 2H), -0.04 (d, *J* = 5.2 Hz, 9H).

***tert-butyl 3-(7-(difluoromethyl)-3,4-dihydroquinolin-1(2H)-yl)-1-((2-(trimethylsilyl)ethoxy)methyl)-1,4,6,7-tetrahydro-5H-pyrazolo[4,3-c]pyridine-5-carboxylate (22)***

Step b: A mixture of Intermediate **20**(4.8 g, 10 mmol), 7-(difluoromethyl)-1,2,3,4-tetrahydroquinoline **21** (2.2 g, 12 mmol), RuPhos Pd G2 (777 mg, 1.0 mmol), RuPhos (447 mg, 1.0 mmol), and Sodium *tert*-butoxide (2.4 g, 25 mmol) in 1,4-dioxane (120 mL) was degassed and purged 3 times with N_2_, and then the mixture was stirred at 100 °C for 12 h. After that, the mixture was cooled, diluted with DCM, filtered through Celite, the filter cake was washed with DCM, and the filtrate was concentrated under reduced pressure. The residue was purified by flash column chromatography (0–5% MeOH/DCM) to give intermediate **22** (3.4 g, yield: 64%) as yellow solid. ^1^H NMR (400 MHz, DMSO-*d*_6_) δ 7.14 (d, *J* = 7.7 Hz, 1H), 6.83 (d, *J* = 7.3 Hz, 2H), 6.80 (td, *J* = 55.2, 6.8 Hz, 2H), 6.60 (s, 1H), 5.30 (s, 2H), 3.98 (s, 2H), 3.65 – 3.52 (m, 6H), 2.79 (dt, *J* = 28.3, 6.1 Hz, 4H), 1.95 (p, *J* = 6.2 Hz, 2H), 1.36 (s, 9H), 0.83 (t, *J* = 7.2 Hz, 2H), -0.06 (s, 9H).

***tert-butyl 3-(6-bromo-7-(difluoromethyl)-3,4-dihydroquinolin-1(2H)-yl)-1-((2-(trimethylsilyl)ethoxy)methyl)-1,4,6,7-tetrahydro-5H-pyrazolo[4,3-c]pyridine-5-carboxylate (23)***

Step c: To a solution of intermediate **22** (3.0 g, 5.6 mmol) in DCM (80 mL) was added NBS (997 mg, 5.6 mmol) in portions under ice-bath condition. After 30 min, the reaction mixture was diluted with DCM, washed with aqueous Na_2_S_2_O_3_ followed by brine, and the organic layers were concentrated under reduced pressure. The resulting residue was purified by flash column chromatography (0−5% MeOH/DCM) to give intermediate **23** (2.8 g, yield: 82%) as a yellow solid. ^1^H NMR (400 MHz, DMSO-*d*_6_) δ 7.37 (s, 1H), 6.92 (t, *J* = 54.2 Hz, 1H), 6.73 (s, 1H), 5.31 (s, 2H), 4.02 (s, 2H), 3.64 – 3.53 (m, 6H), 2.87 – 2.73 (dt, *J* = 28.3, 6.1 Hz, 4H), 1.94 (p, *J* = 6.2 Hz, 2H), 1.37 (s, 9H), 0.82 (t, *J* = 7.2, 2H), -0.06 (s, 9H).

***tert-butyl 3-(7-(difluoromethyl)-6-(1-methyl-1H-pyrazol-4-yl)-3,4-dihydroquinolin-1(2H)-yl)-1-((2-(trimethylsilyl)ethoxy)methyl)-1,4,6,7-tetrahydro-5H-pyrazolo[4,3-c]pyridine-5-carboxylate (25)***

Step d: A mixture of intermediate **23** (1.4 g, 2.28 mmol), 1-methyl-4-(4,4,5,5-tetramethyl-1,3,2-dioxaborolan-2-yl)-1H-pyrazole **24** (570.0 mg, 2.74 mmol), Pd(OAc)_2_ (51.1 mg, 0.23 mmol), Pph_3_ (119.5 mg, 0.46) and K_2_CO_3_ (629.3 mg, 4.56 mmol) were added into 50 mL of 1,4-Dioxane : H_2_O (5:2). The mixture was degassed and purged 3 times with N_2_, then stirred at 100 °C for 3 h. After that, the mixture was cooled, filtered through Celite, the filter cake was washed with DCM, and the filtrate was concentrated under reduced pressure. The residue was purified by flash column chromatography (0−5% MeOH/DCM) to give intermediate **25** as white solid (1.1 g, yield: 80%)**.** ^1^H NMR (400 MHz, DMSO-*d*_6_) δ 9.06 (s, 1H), 7.79 – 7.74 (m, 1H), 7.53 – 7.48 (m, 1H), 7.17 – 7.11 (m, 1H), 6.83 – 6.78 (m, 1H), 6.76 (td, *J* = 55.2, 6.8 Hz, 1H) 5.34 (d, *J* = 24.6 Hz, 2H), 4.02 (s, 2H), 3.88 (d, *J* = 2.1 Hz, 3H), 3.64 – 3.53 (m, 6H), 2.81 (dt, *J* = 30.9, 6.1 Hz, 4H), 1.97 (t, *J* = 5.8 Hz, 2H), 1.36 (s, 9H), 0.90 – 0.79 (m, 2H), -0.01 – -0.06 (m, 9H).

***7-(difluoromethyl)-6-(1-methyl-1H-pyrazol-4-yl)-1-(1-((2-(trimethylsilyl)ethoxy)methyl)-4,5,6,7-tetrahydro-1H-pyrazolo[4,3-c]pyridin-3-yl)-1,2,3,4-tetrahydroquinoline (26)***

Step e: Intermediate **25** (1.0 g, 1.63 mmol) was added into [formic acid](https://www.sigmaaldrich.com/US/en/substance/formicacid460364186) (8 mL). The mixture was stirred at rt for 4 h. The reaction was monitored by LC-MS until completion. The mixture was concentrated and purified by pre-HPLC to give intermediate **26** (587 mg, yield: 70%). ^1^H NMR (400 MHz, DMSO-*d*_6_) δ 7.78 (s, 1H), 7.51 (t, *J* = 1.7 Hz, 1H), 7.16 (s, 1H), 6.96 – 6.66 (m, 2H), 5.37 (s, 2H), 3.84 (s, 2H), 3.63 – 3.56 (m, 4H), 3.47 – 3.37 (m, 2H), 3.03 (t, *J* = 6.0 Hz, 2H), 2.85 (t, *J* = 6.4 Hz, 2H), 1.98 (q, *J* = 5.9 Hz, 2H), 0.87 (t, *J* = 8.6, 2H), -0.015 (s, 9H).

***7-(difluoromethyl)-1-(5-isopropyl-1-((2-(trimethylsilyl)ethoxy)methyl)-4,5,6,7-tetrahydro-1H-pyrazolo[4,3-c]pyridin-3-yl)-6-(1-methyl-1H-pyrazol-4-yl)-1,2,3,4-tetrahydroquinoline (27)***

Step f: A solution of intermediate **26** (514 mg, 1.0 mmol) in DCM (5 mL) at room temperature was added acetone (580 mg, 10.0 mmol). The mixture was stirred at room temperature for 30 min. Then sodium triacetoxyborohydride (318 mg, 1.5 mmol) was added to this reaction, and the mixture was stirred at room temperature for 3 h. Monitor the reaction through LC/MS until the intermediate **26** was completely consumed and then the mixture was concentrated under reduced pressure. The crude residue was purified by pre-HPLC to give intermediate **27** (456 mg, yield: 82%). ^1^H NMR (400 MHz, DMSO-*d*_6_) δ 7.78 (s, 1H), 7.52 (d, *J* = 0.8 Hz, 1H), 7.16 (s, 1H), 6.87 (s, 1H), 6.82 (td, *J* = 55.1 Hz, 6.8 Hz, 1H), 5.46 – 5.31 (m, 2H), 4.01 – 3.95 (m, 2H), 3.94 – 3.89 (m, 1H), 3.88 (s, 3H), 3.81 – 3.73 (m, 1H), 3.72 – 3.52 (m, 4H), 3.40 – 3.28 (m, 1H), 3.23 – 3.15 (m, 1H), 3.12 – 3.00 (m, 1H), 2.86 (t, *J* = 6.4 Hz, 2H), 1.99 (p, *J* = 6.1 Hz, 2H), 1.27 (dd, *J* = 10.6, 6.5 Hz, 6H), 0.88 (dt, *J* = 8.9, 6.6 Hz, 2H), -0.06 (s,9H).

***7-(difluoromethyl)-1-(5-isopropyl-4,5,6,7-tetrahydro-1H-pyrazolo[4,3-c]pyridin-3-yl)-6-(1-methyl-1H-pyrazol-4-yl)-1,2,3,4-tetrahydroquinoline (28)***

Step g: Intermediate **27** (400 mg, 0.72 mmol) was dissolved in THF (8 mL). Followed by 1 mL of tetrabutylammonium fluoride solution (1 M, in THF) was added into the mixture. The mixture was stirred and refluxed at 66 °C overnight. Monitor the reaction through LC/MS until the intermediate **27** was completely consumed, and then the mixture was concentrated under reduced pressure. The crude residue was purified by pre-HPLC to give intermediate **28** (159 mg, yield: 52%) as white solid. ^1^H NMR (400 MHz, DMSO-*d*_6_) δ 7.77 (d, *J* = 0.8 Hz, 1H), 7.51 (d, *J* = 0.8 Hz, 1H), 7.14 (s, 1H), 6.87 (s, 1H), 6.81 (td, *J* = 55.2 Hz, 6.8 Hz, 1H), 3.95 – 3.90 (m, 2H), 3.87 (s, 3H), 3.70 – 3.53 (m, 4H), 3.37 – 3.25 (m, 1H), 3.21 – 3.09 (m, 1H), 3.01 (dd, *J* = 16.9, 4.4 Hz, 1H), 2.85 (t, *J* = 6.4 Hz, 2H), 1.99 (p, *J* = 6.5 Hz, 2H), 1.29 (dd, *J* = 8.0, 6.6 Hz, 6H).

***methyl (1r,4r)-4-(3-(7-(difluoromethyl)-6-(1-methyl-1H-pyrazol-4-yl)-3,4-dihydroquinolin-1(2H)-yl)-5-isopropyl-4,5,6,7-tetrahydro-1H-pyrazolo[4,3-c]pyridin-1-yl)cyclohexane-1-carboxylate (30)***

Step h: A solution of Intermediate **28** (150 mg, 0.35 mmol), Cs_2_CO_3_ (287.8 mg, 0.88 mmol) and methyl (1s,4s)-4-((methylsulfonyl)oxy)cyclohexane-1-carboxylate **29** (181.7 mg, 0.77 mmol) in DMF (5 mL). The mixture was stirred at 80 °C for overnight. Monitor the reaction through LC/MS until the intermediate **28** was completely consumed. Then the mixture was concentrated under reduced pressure. The crude residue was purified by pre-HPLC to give intermediate **30** (69 mg, yield: 35%) as yellow solid. (Two isomers are generated by substitution at the two nitrogen atoms of the pyrazole. In C18 reversed-phase chromatography, the desired compound has a slightly longer retention time than the other isomer). ^1^H NMR (400 MHz, DMSO-*d*_6_) δ 7.75 (d, *J* = 0.8 Hz, 1H), 7.49 (d, *J* = 0.8 Hz, 1H), 7.07 (s, 1H), 6.78 (s, 1H), 6.78 (td, *J* = 55.2 Hz, 6.8 Hz, 1H), 4.05 – 3.95 (m, 1H), 3.87 (s, 3H), 3.61 (s, 3H), 3.59 – 3.53 (m, 2H), 3.16 (s, 2H), 2.88 – 2.78 (m, 3H), 2.72 (s, 4H), 2.38 (tt, *J* = 12.4, 8.8, 3.6 Hz, 1H), 2.06 – 1.76 (m, 8H), 1.53 (qd, *J* = 13.0, 3.8 Hz, 2H), 0.97 (d, *J* = 6.5 Hz, 6H).

***(1r,4r)-4-(3-(7-(difluoromethyl)-6-(1-methyl-1H-pyrazol-4-yl)-3,4-dihydroquinolin-1(2H)-yl)-5-isopropyl-4,5,6,7-tetrahydro-1H-pyrazolo[4,3-c]pyridin-1-yl)cyclohexane-1-carbaldehyde (31)***

Step i: Intermediate **31** (60 mg, 0.10 mmol) was dissolved in anhydrous DCM (15 mL) and the solution was degassed and charged with N_2_ 3 times. The mixture was stirred at -78 ^o^C for 5 min followed by diisobutylaluminum hydride (25% in toluene, 0.24 mL, 0.42 mmol) was added dropwise into the mixture. The reaction mixture was stirred at -78 ^o^C for an additional 1.5 h. The reaction was then quenched with aqueous potassium sodium tartrate, after which the mixture was warmed to room temperature and stirred for an additional 30 minutes. The resulting mixture was diluted with DCM and filtered through Celite, and the filter cake was washed with DCM. The combined organic layers were concentrated under reduced pressure. The crude residue was used directly in the next step without further purification. LC-MS *m*/*z* [M+H]^+^ 537.31.

***N-((1r,4r)-4-((3-chloro-4-cyanophenyl)(methyl)amino)cyclohexyl)-6-(4-(((1r,4r)-4-(3-(7-(difluoromethyl)-6-(1-methyl-1H-pyrazol-4-yl)-3,4-dihydroquinolin-1(2H)-yl)-5-isopropyl-4,5,6,7-tetrahydro-1H-pyrazolo[4,3-c]pyridin-1-yl)cyclohexyl)methyl)piperazin-1-yl)pyridazine-3-carboxamide (Neg-1)***

Step j: Compound **Neg-1** was synthesized from intermediate **31** and intermediate **18** following a similar procedure to that of **DALTAC-1** (yield 30%, two steps). ^1^H NMR (400 MHz, DMSO-*d*_6_) δ 8.50 (d, *J* = 8.4 Hz, 1H), 7.85 (d, *J* = 9.3 Hz, 1H), 7.76 (s, 1H), 7.61 (d, *J* = 8.9 Hz, 1H), 7.51 (s, 1H), 7.36 (d, *J* = 9.7 Hz, 1H), 7.12 (s, 1H), 6.99 – 6.58 (m, 4H), 4.20 – 3.47 (m, 14H), 3.27 – 3.10 (m, 1H), 3.10 – 2.97 (m, 1H), 2.86 (s, 5H), 2.76 – 2.65 (m, 1H), 2.60 – 2.53 (m, 1H), 2.19 (s, 2H), 2.05 – 1.53 (m, 18H), 1.52 – 0.68 (m, 12H). LC-MS *m*/*z* [M+H]^+^ 974.45.

**Scheme 4: Synthesis of Neg-2.**

***Reagents and conditions*:** (a) K_2_CO_3_, DMF, 70°C, 4 h; (b) DCM, TFA, reflex; (c) HATU, DMF, Et_3_N, rt; (d) DIPEA, DMF, 55°C; (e) Et_3_N, Sodium triacetoxyborohydride, DCM, rt.

***(1r,4r)-4-phenoxycyclohexan-1-amine (34)***

Step a: A solution of phenol **32** (420.0 mg, 4.4 mmol), K_2_CO_3_ (924.9 mg, 6.7 mmol) and compound **33** (943.0 mg, 4.4 mmol) in DMF (12 mL). The mixture was stirred at 70 °C for 4 h. After complete conversion, the mixture was diluted with H_2_O, extracted with EtOAc, dried, and concentrated.

Step b: The above crude product was dissolved in 10 mL of DCM : TFA (4:1) and stirred at room temperature for an additional 4 h. Monitor the reaction through LC/MS until the reaction was completed. Then the mixture was concentrated and purified by flash column chromatography (0–20% MeOH/DCM) to give intermediate **34** (337 mg, yield: 40%) as white solid. ^1^H NMR (400 MHz, DMSO-*d*_6_) δ 7.31 – 7.23 (m, 2H), 6.99 – 6.88 (m, 3H), 4.34 – 4.12 (m, 1H), 3.16 – 3.00 (m, 1H), 2.15 – 2.04 (m, 2H), 2.04 – 1.94 (m, 2H), 1.56 – 1.35 (m, 4H).

***N-((1r,4r)-4-phenoxycyclohexyl)-6-(piperazin-1-yl)pyridazine-3-carboxamide (35)***

Step c and d: Compound **35** was synthesized from intermediate **34** following a similar procedure to that of intermediate **18** (yield 30%, two steps). ^1^H NMR (400 MHz, DMSO-*d*_6_) δ 8.61 (d, *J* = 8.3 Hz, 1H), 7.91 (d, *J* = 9.5 Hz, 1H), 7.42 (d, *J* = 9.6 Hz, 1H), 7.33 – 7.22 (m, 2H), 6.97 – 6.93 (m, 2H), 6.93 – 6.89 (m, 1H), 4.29 (tt, *J* = 10.2, 4.1 Hz, 1H), 3.94 – 3.80 (m, 5H), 3.14 (t, *J* = 5.1 Hz, 4H), 2.09 (d, *J* = 5.9 Hz, 2H), 1.91 (d, *J* = 13.0 Hz, 2H), 1.62 (q, *J* = 12.9 Hz, 2H), 1.49 (q, *J* = 10.9, 9.3 Hz, 2H).

***6-(4-(((1r,4r)-4-(5-acetyl-3-(7-(difluoromethyl)-6-(1-methyl-1H-pyrazol-4-yl)-3,4-dihydroquinolin-1(2H)-yl)-4,5,6,7-tetrahydro-1H-pyrazolo[4,3-c]pyridin-1-yl)cyclohexyl)methyl)piperazin-1-yl)-N-((1r,4r)-4-phenoxycyclohexyl)pyridazine-3-carboxamide (Neg-2)***

Step e: Compound **Neg-2** was synthesized from intermediate **35** and intermediate **16** following a similar procedure to that of compound **DALTAC-1** (yield 65%). ^1^H NMR (400 MHz, DMSO-*d*_6_) δ 8.56 (dd, *J* = 8.3, 2.3 Hz, 1H), 7.83 (dd, *J* = 9.5, 1.9 Hz, 1H), 7.76 (s, 1H), 7.50 (s, 1H), 7.35 (dd, *J* = 9.7, 3.6 Hz, 1H), 7.31 – 7.24 (m, 2H), 7.11 (d, *J* = 3.0 Hz, 1H), 6.98 – 6.88 (m, 4H), 6.82 – 6.77 (m, 1H), 4.29 (tt, *J* = 10.4, 4.2 Hz, 1H), 4.23 – 4.07 (m, 2H), 4.07 – 3.95 (m, 1H), 3.87 (s, 4H), 3.79 – 3.63 (m, 5H), 3.63 – 3.55 (m, 2H), 2.89 – 2.81 (m, 3H), 2.49 – 2.44 (m, 2H), 2.34 (d, *J* = 7.6 Hz, 1H), 2.18 (dd, *J* = 7.4, 3.8 Hz, 1H), 2.15 – 2.05 (m, 4H), 2.01 – 1.86 (m, 8H), 1.86 – 1.69 (m, 3H), 1.62 (q, *J* = 12.7, 3H), 1.54 – 1.42 (m, 3H), 1.33 – 1.18 (m, 5H). LC-MS *m*/*z* [M+H]^+^ 902.63.

**Scheme 5: Synthesis of Compounds JZY3222 and ARi (JZY3221).**

***Reagents and conditions*:** (a) Cs_2_CO_3_, DMF, 80 °C, 8h; (b) HATU, DMF, Et_3_N, rt, 15 min.

Intermediate **36** was prepared according to the reported procedures.^1^

***1-(1-cyclohexyl-3-(7-(difluoromethyl)-6-(1-methyl-1H-pyrazol-4-yl)-3,4-dihydroquinolin-1(2H)-yl)-1,4,6,7-tetrahydro-5H-pyrazolo[4,3-c]pyridin-5-yl)ethan-1-one (JZY3222)***

Step a: To a solution of intermediate **36** (43 mg, 0.1 mmol), Cs_2_CO_3_ (81.5 mg, 0.25 mmol) in DMF (5 mL) was added bromocyclohexane **37** (48.9 mg, 0.3 mmol). The mixture was stirred at 75 ^o^C for 7 h. After that, the mixture was cooled, filtered through Celite, the filter cake was washed with DCM, and the filtrate was concentrated under reduced pressure. The residue was purified by pre-HPLC: CH_3_CN/H_2_O from 35% to 100% in 60 min, flow rate (60 ml/min). The desired product started eluting at ~ 54% MeCN (Two isomers are generated by substitution at the two nitrogen atoms of the pyrazole. In C18 reversed-phase chromatography, the desired compound has a slightly longer retention time than the other isomer). ^1^H NMR (400 MHz, DMSO-*d*_6_) δ 7.76 (s, 1H), 7.50 (s, 1H), 7.10 (s, 1H), 6.81 (d, *J* = 4.2 Hz, 1H), 6.78 (td, *J* = 55.2 Hz, 6.8 Hz, 1H), 4.14 (d, *J* = 20 Hz, 2H) 4.05 – 3.94 (m, 1H), 3.87 (s, 3H), 3.77 – 3.65 (m, 2H), 3.62 – 3.54 (m, 2H), 2.84 (q, *J* = 5.3 Hz, 3.26H), 2.72 (t, *J* = 5.6 Hz, 0.74H), 2.08 (s, 2H), 2.01 – 1.93 (m, 3H), 1.92 – 1.61 (m, 7H), 1.39 (q, *J* = 12.6, 12.2 Hz, 2H), 1.25 – 1.12 (m, 1H). LC-MS *m*/*z* [M+H]^+^ 509.33.

***N-((1r,4r)-4-((3-chloro-4-cyanophenyl)(methyl)amino)cyclohexyl)pyridazine-3-carboxamide (JZY3221)***

Step b: Compound **JZY3221** was synthesized from intermediates **17** and **38** following a similar procedure to that of intermediate **10**. ^1^H NMR (400 MHz, DMSO-*d*_6_) δ 9.41 (dd, *J* = 5.0, 1.7 Hz, 1H), 9.01 (d, *J* = 8.3 Hz, 1H), 8.21 (dd, *J* = 8.4, 1.7 Hz, 1H), 7.91 (dd, *J* = 8.4, 5.1 Hz, 1H), 7.62 (d, *J* = 9.0 Hz, 1H), 6.95 (d, *J* = 2.5 Hz, 1H), 6.84 (dd, *J* = 9.1, 2.5 Hz, 1H), 3.99 – 3.86 (m, 1H), 3.84 – 3.73 (m, 1H), 2.86 (s, 3H), 2.00 – 1.88 (m, 2H), 1.84 – 1.65 (m, 6H).

**
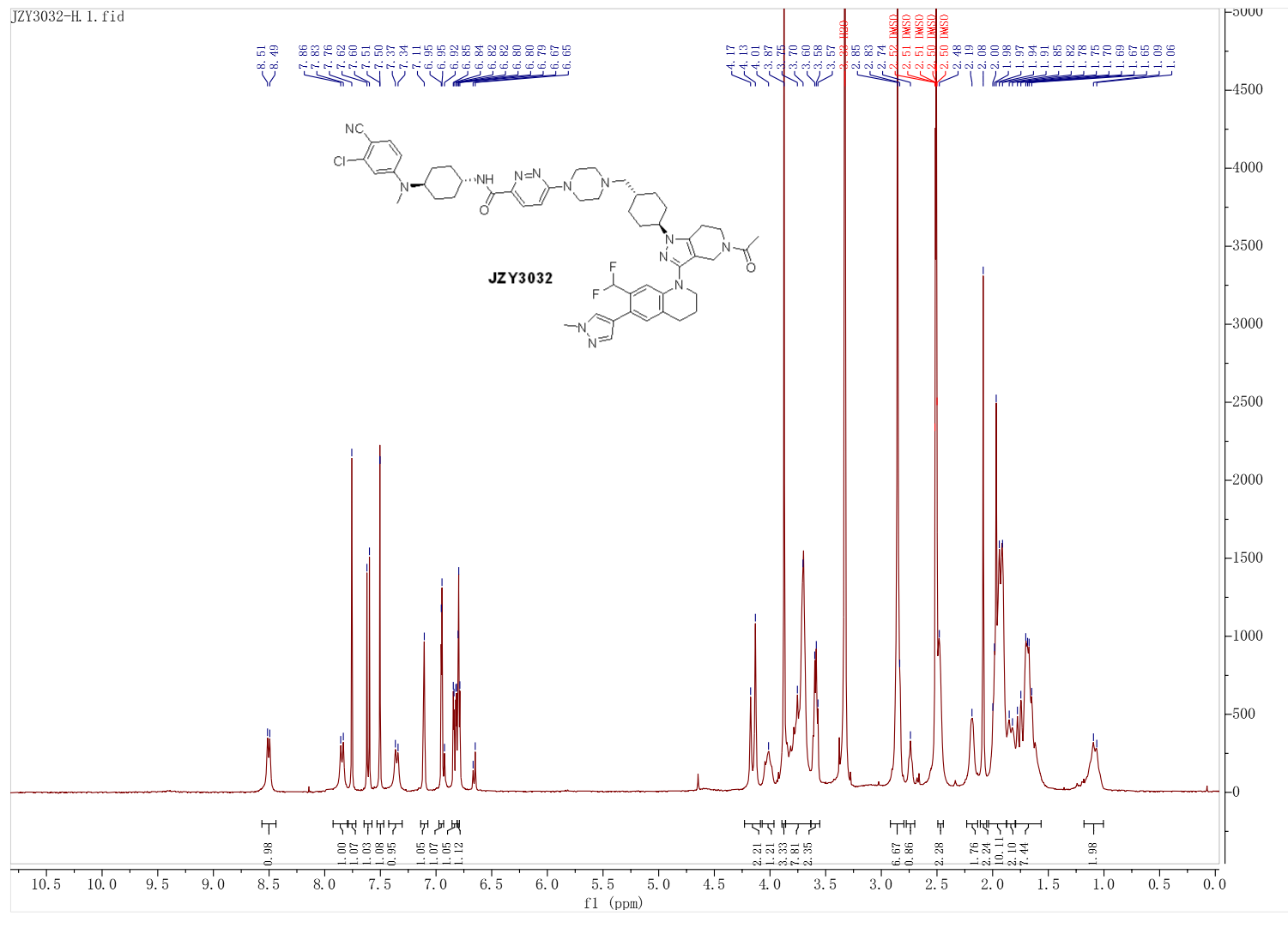
The ^1^H NMR, ^13^C NMR, and LC-MS traces of Compound DALTAC-1 (JZY3032)**

**
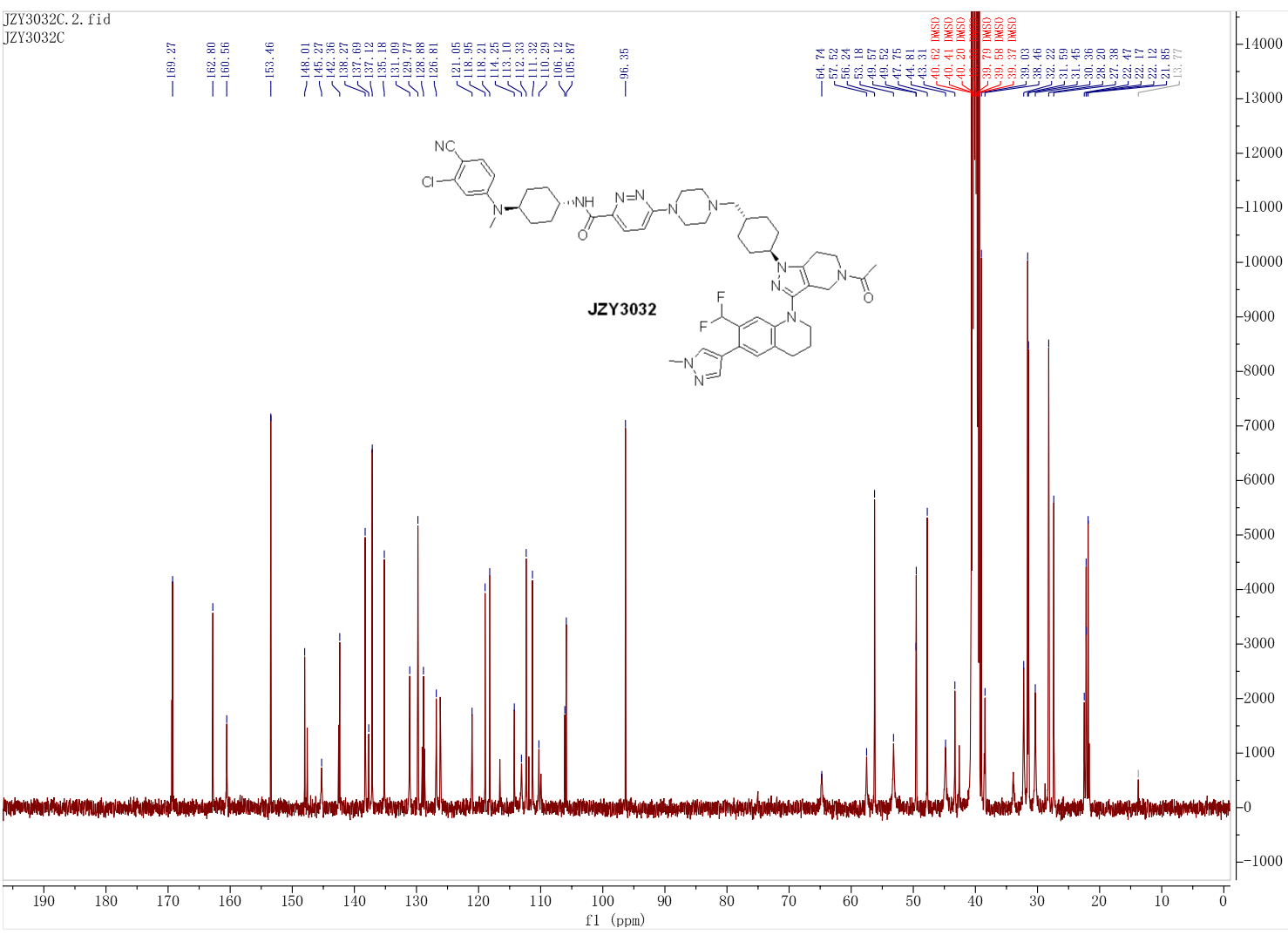
**

**
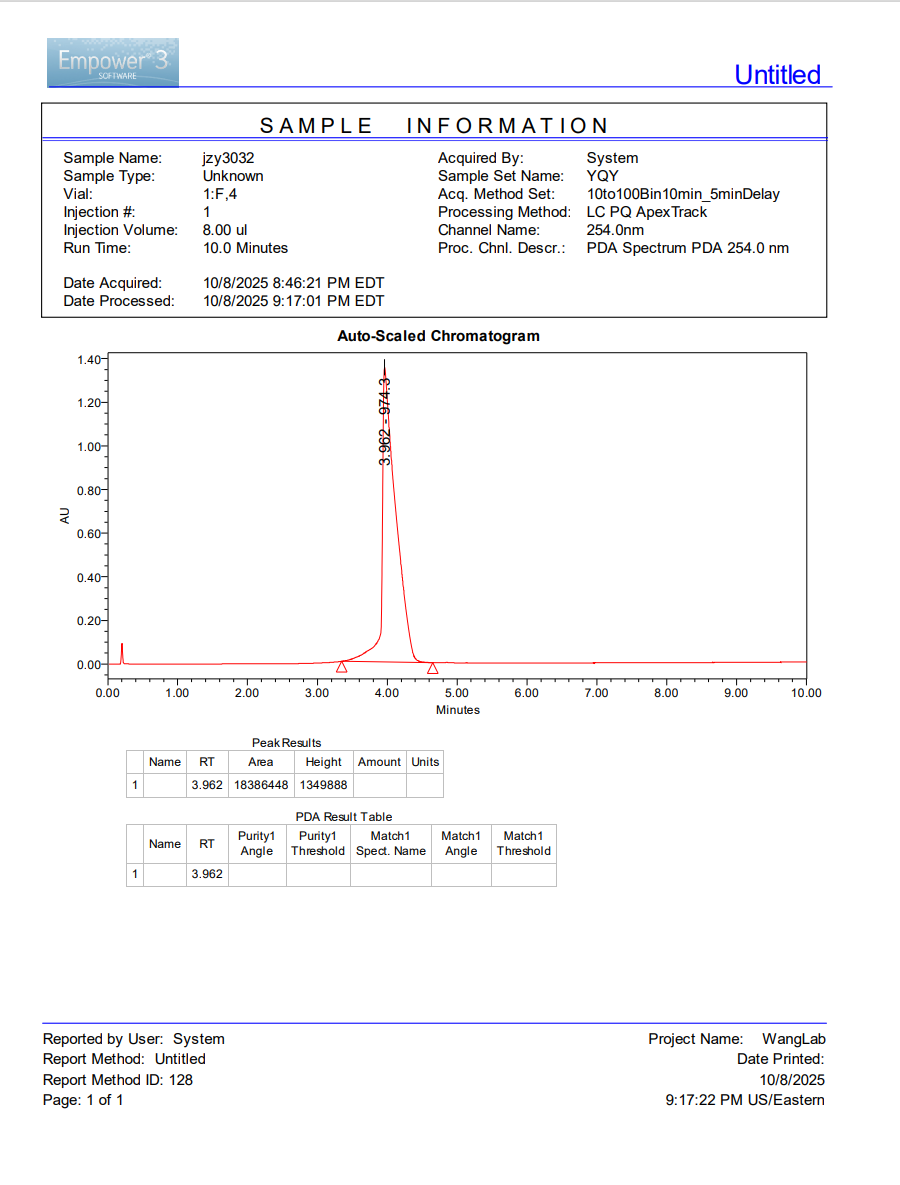
**

**The ^1^H NMR traces of Compound 1-7, Neg-1, Neg-2, JZY3222, and ARi (JZY3221).**

**
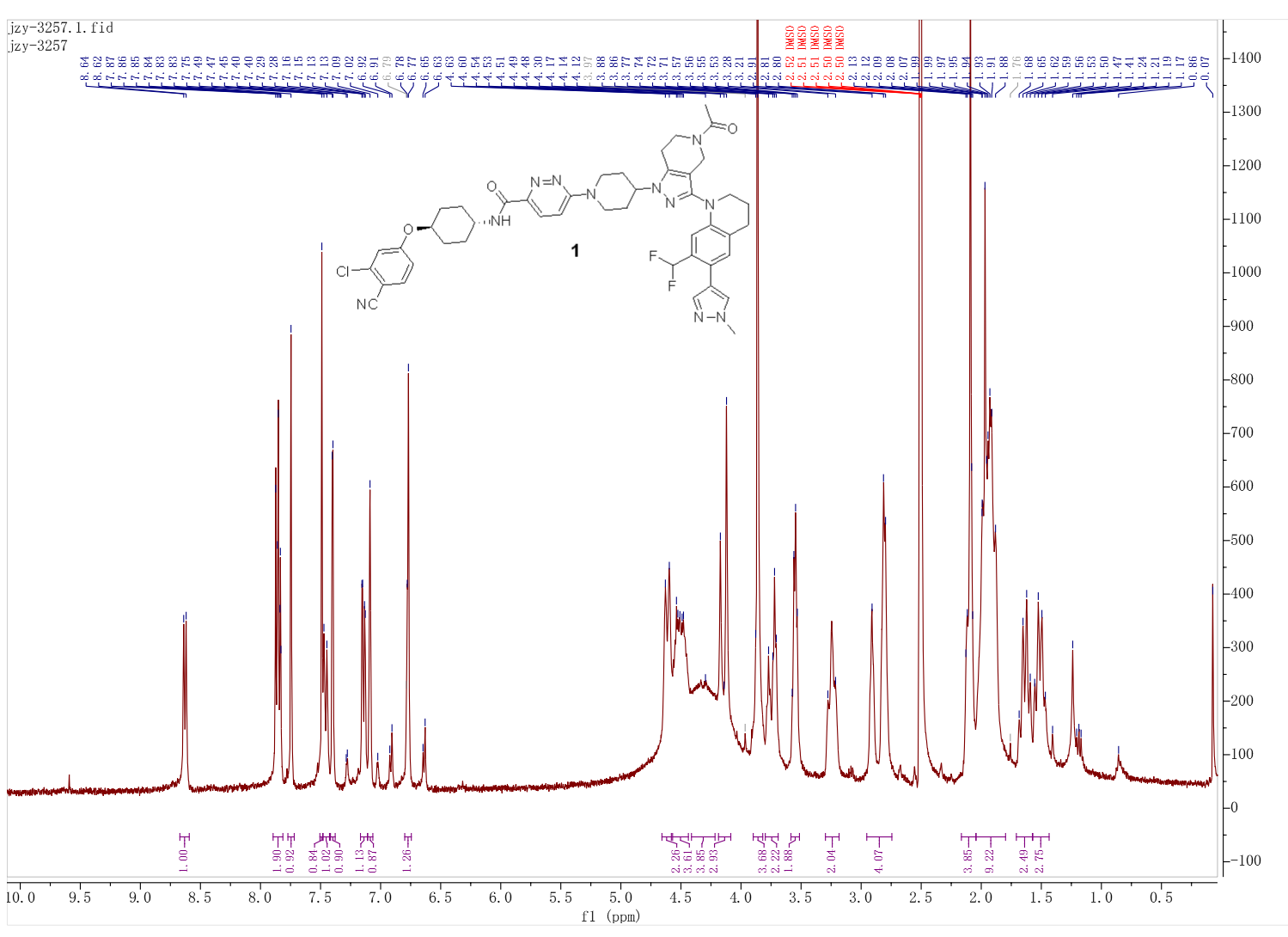
**

**
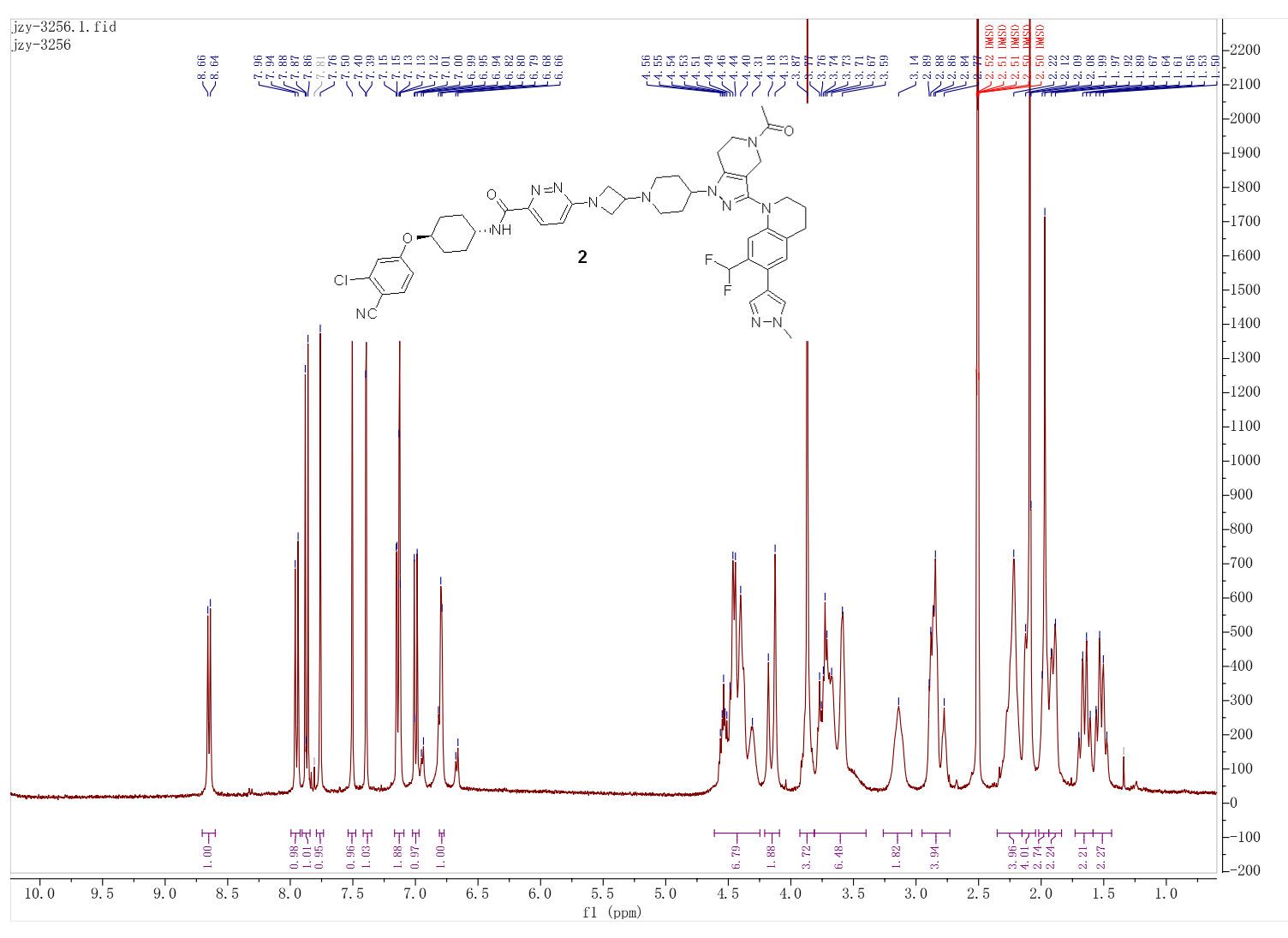
**

**
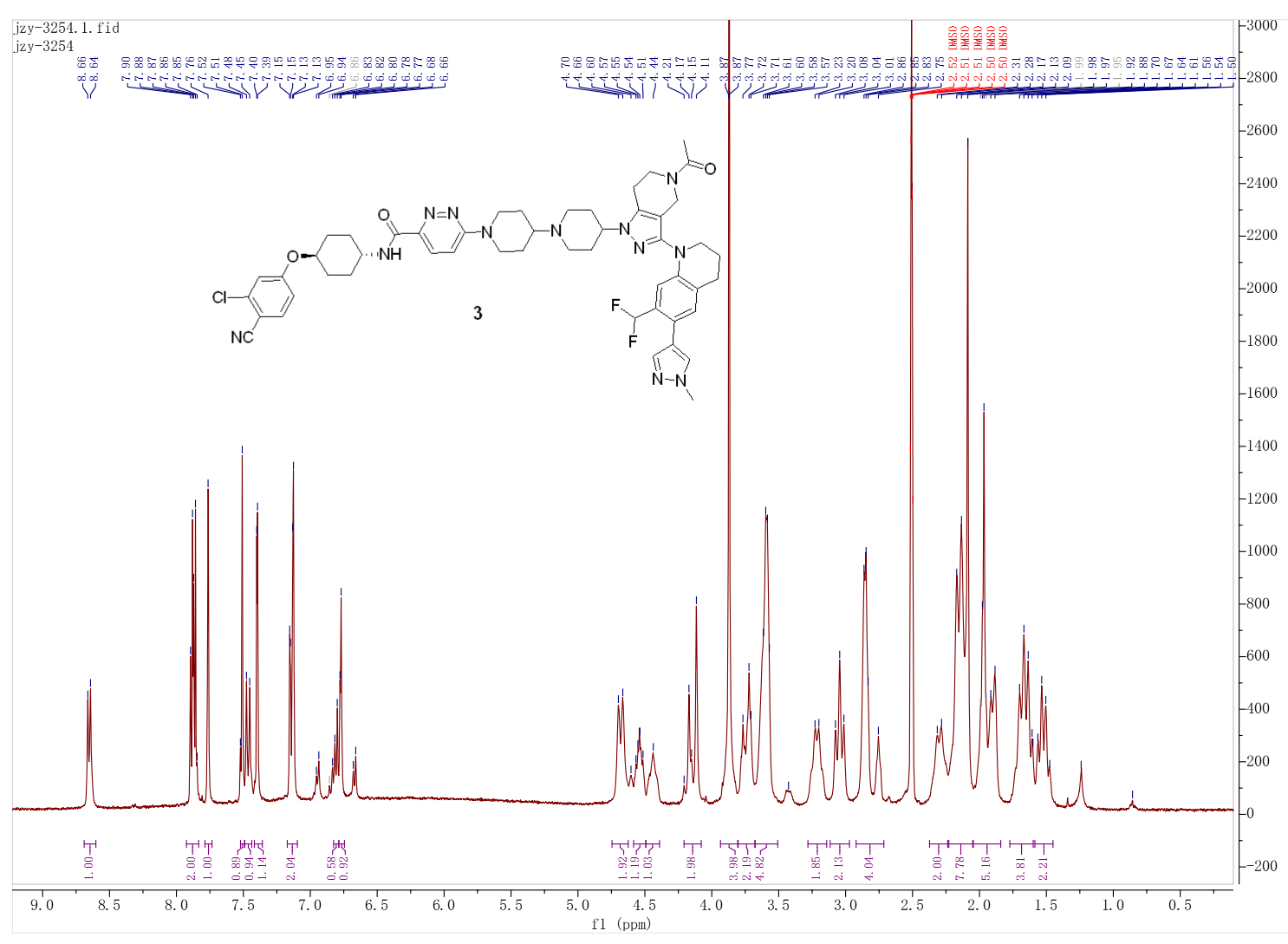
**

**
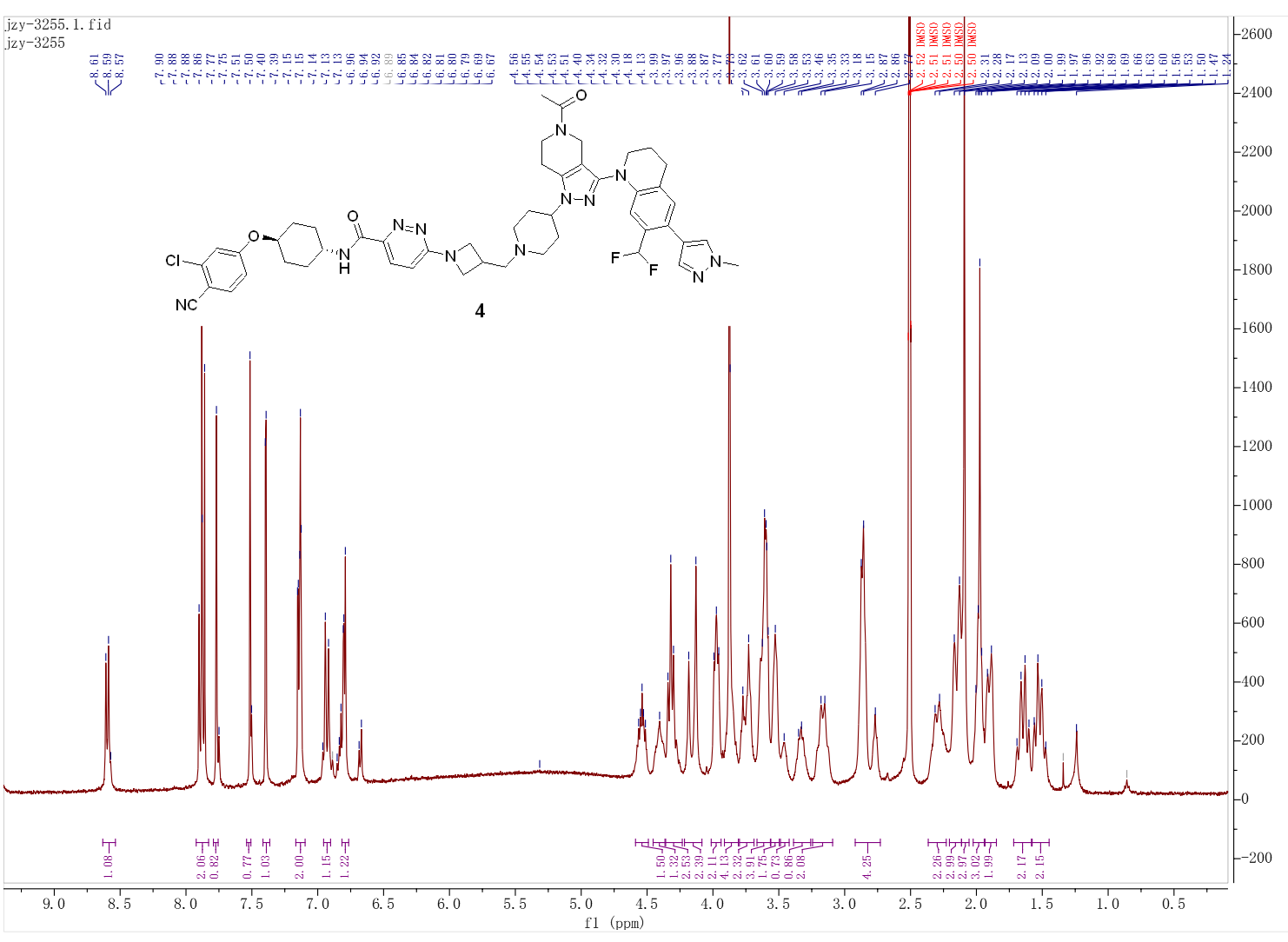

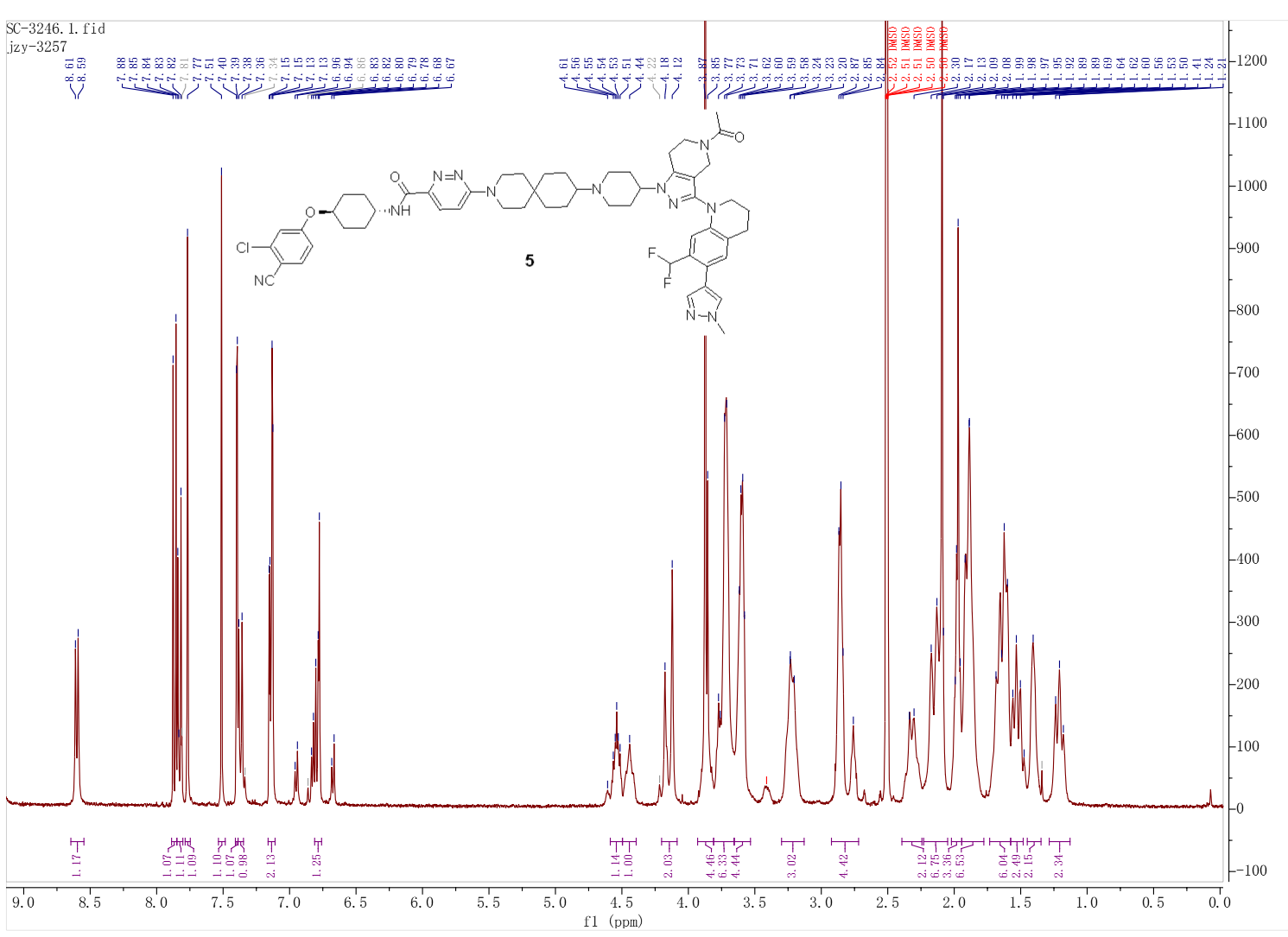
**

**
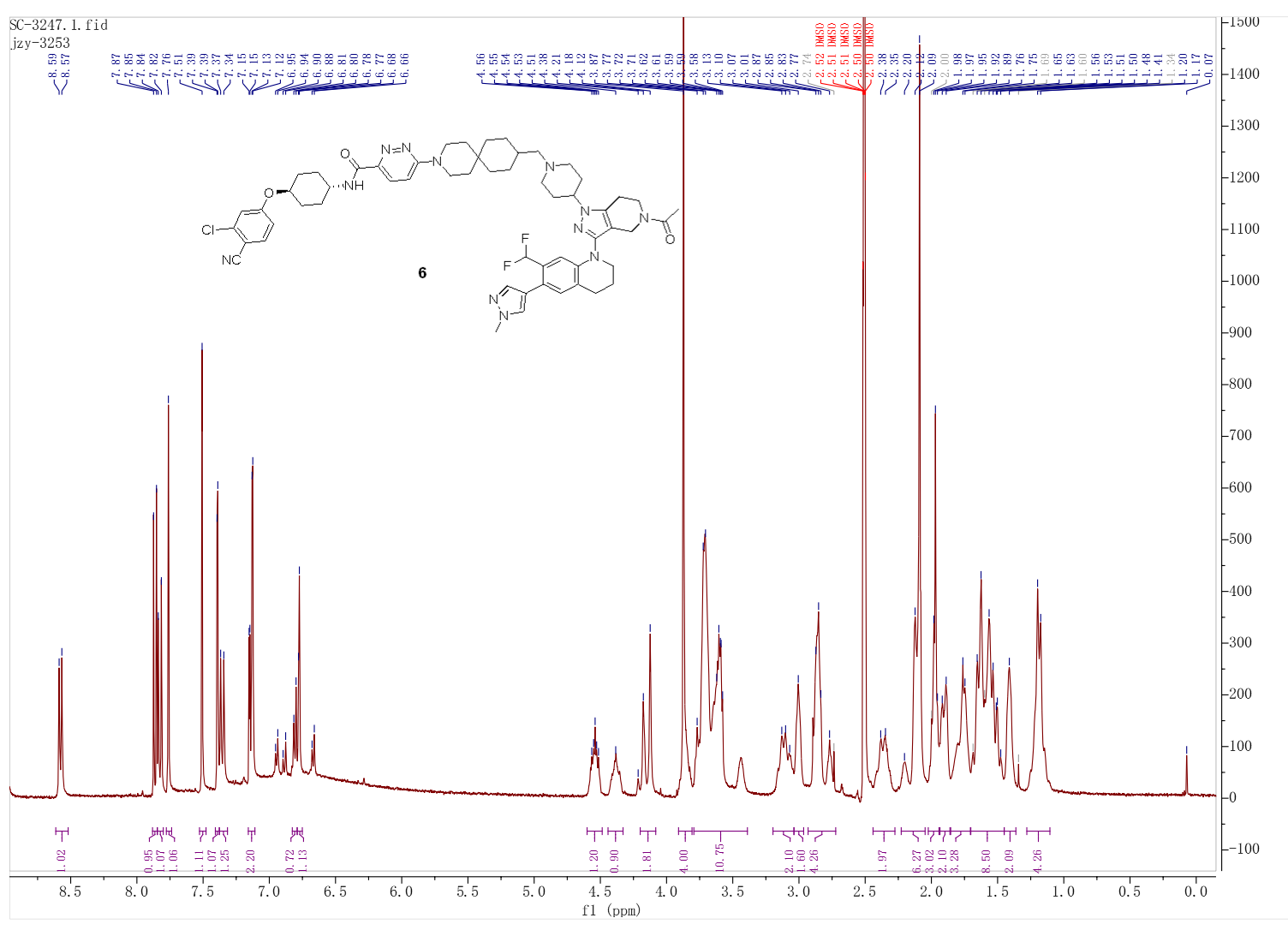

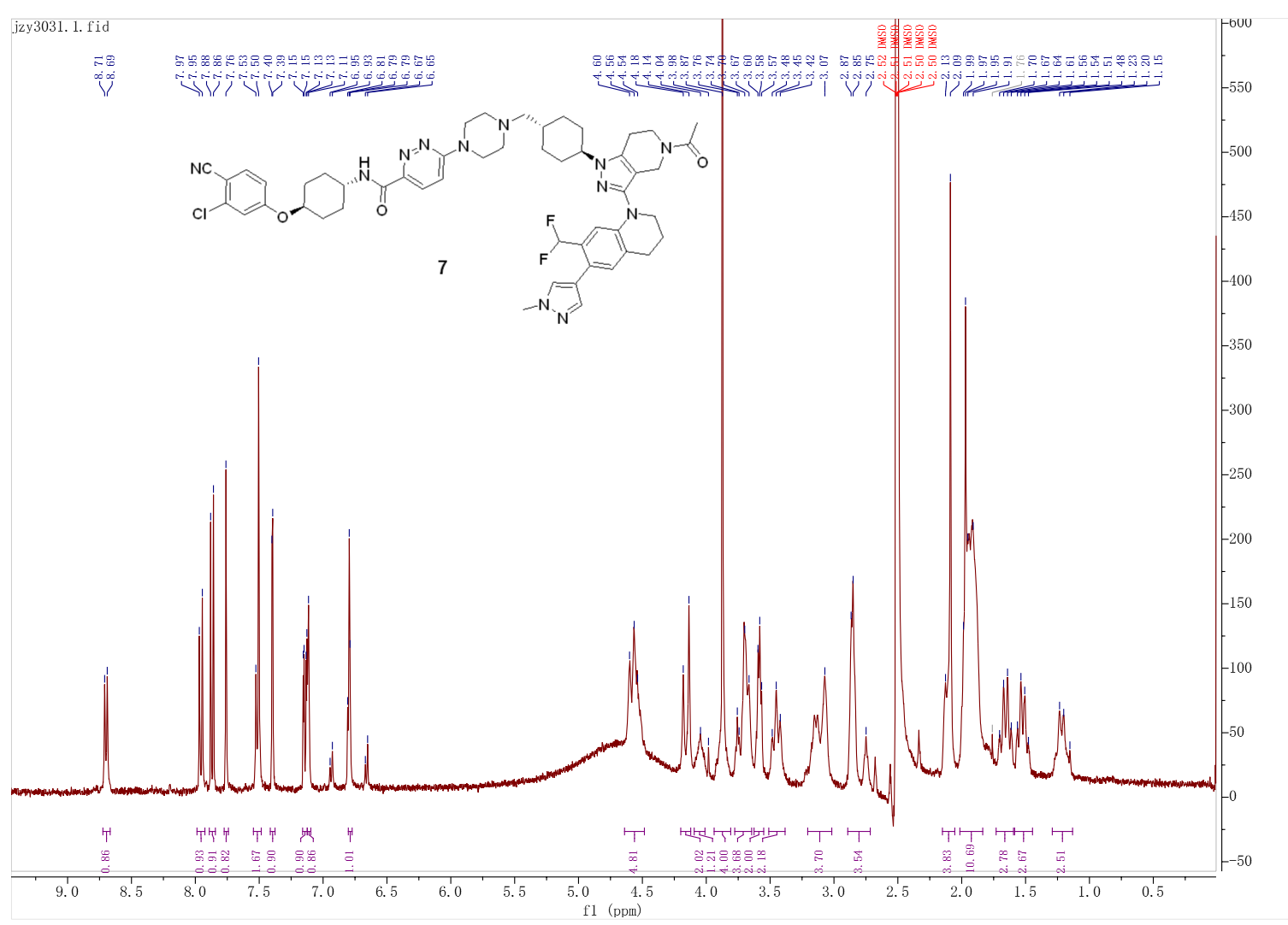
**

**
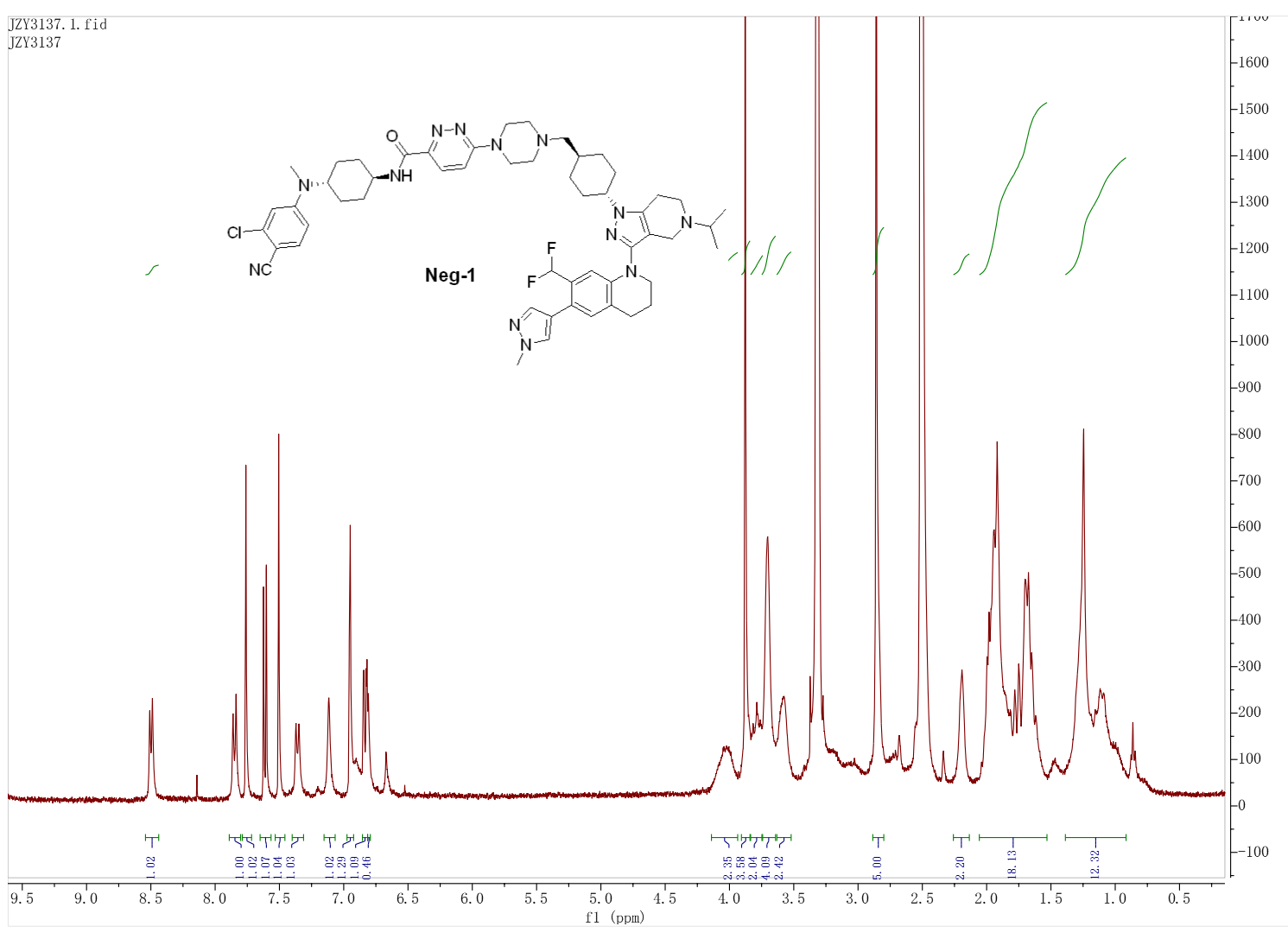
**

**
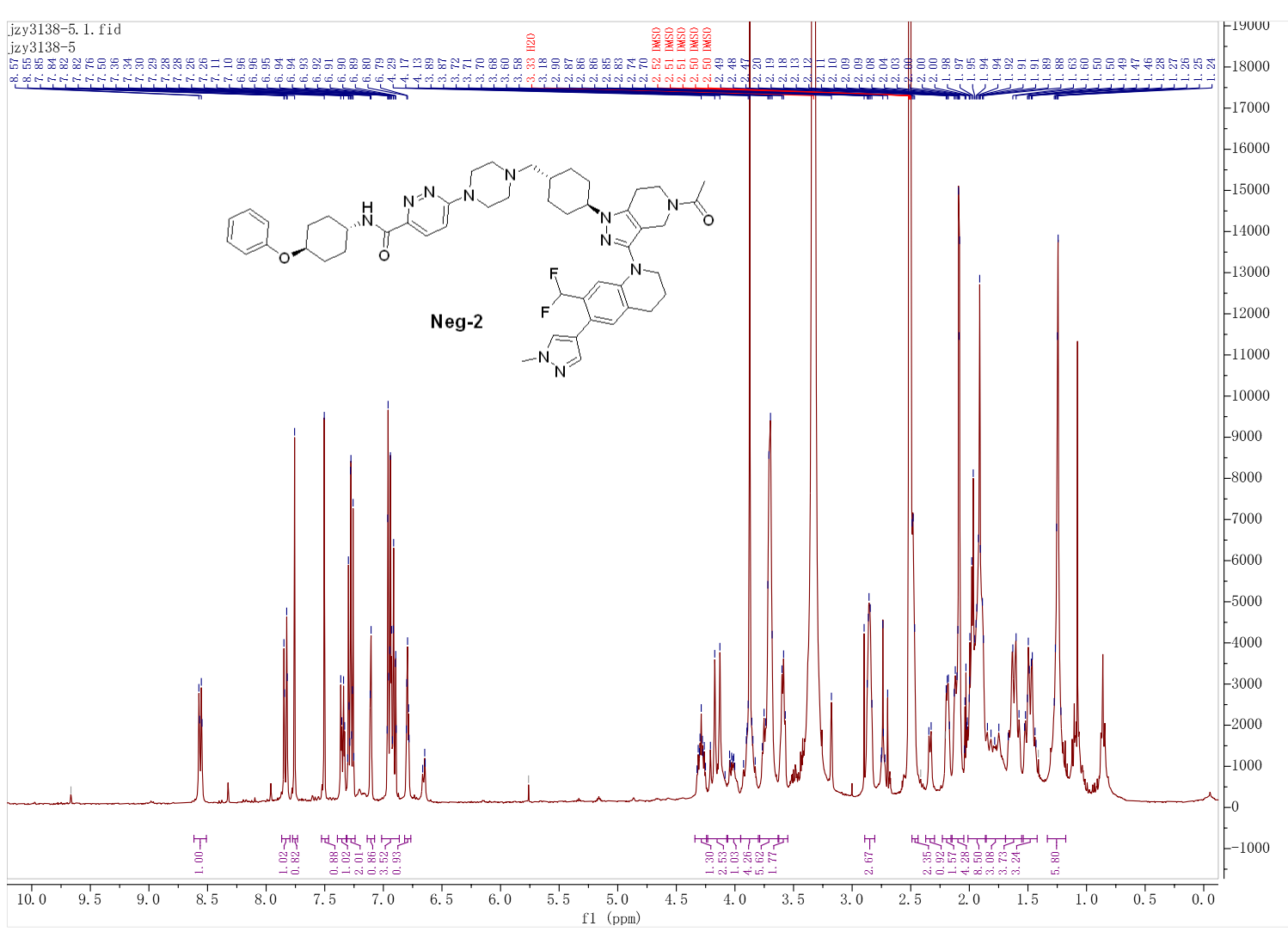
**
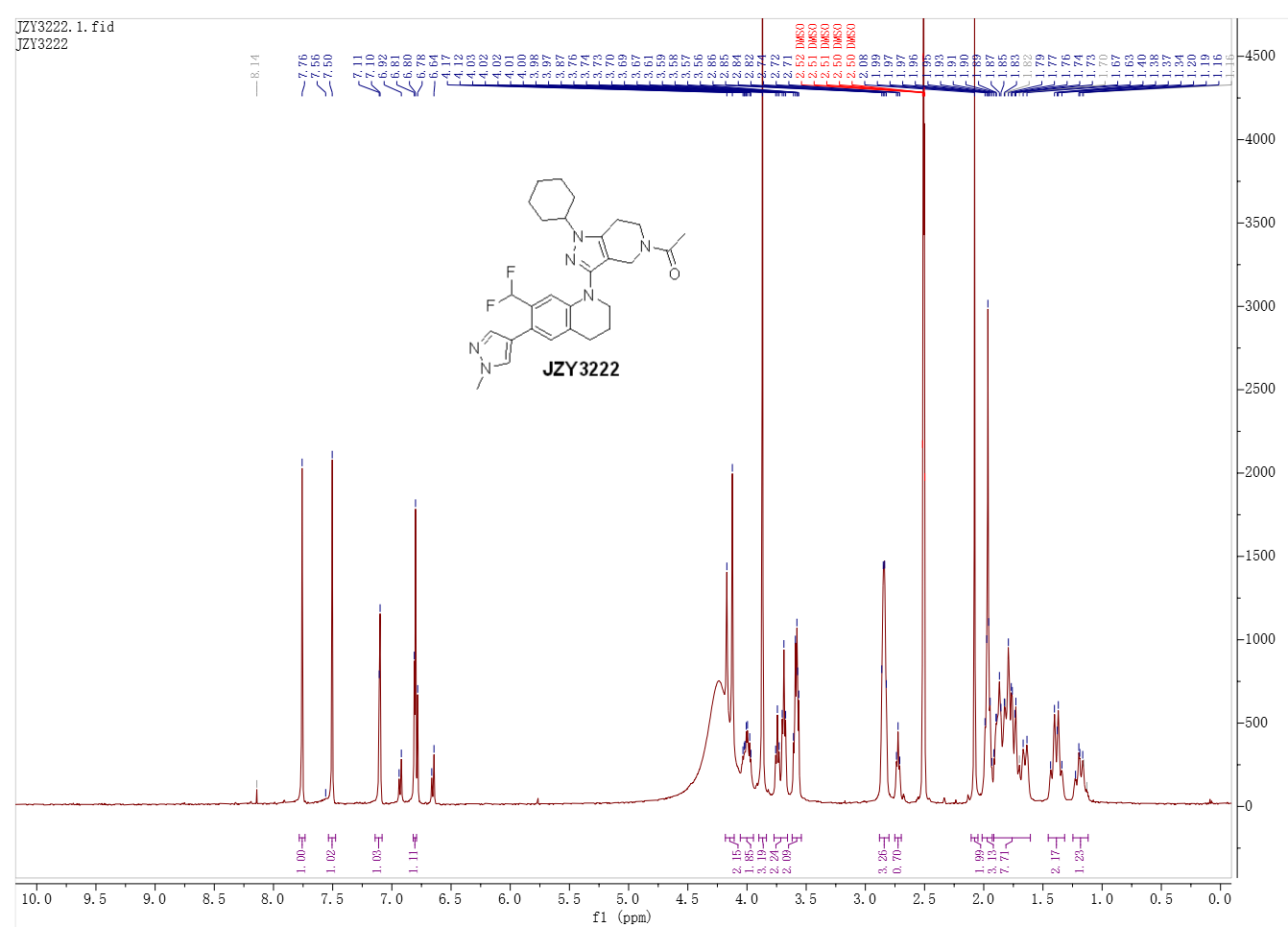


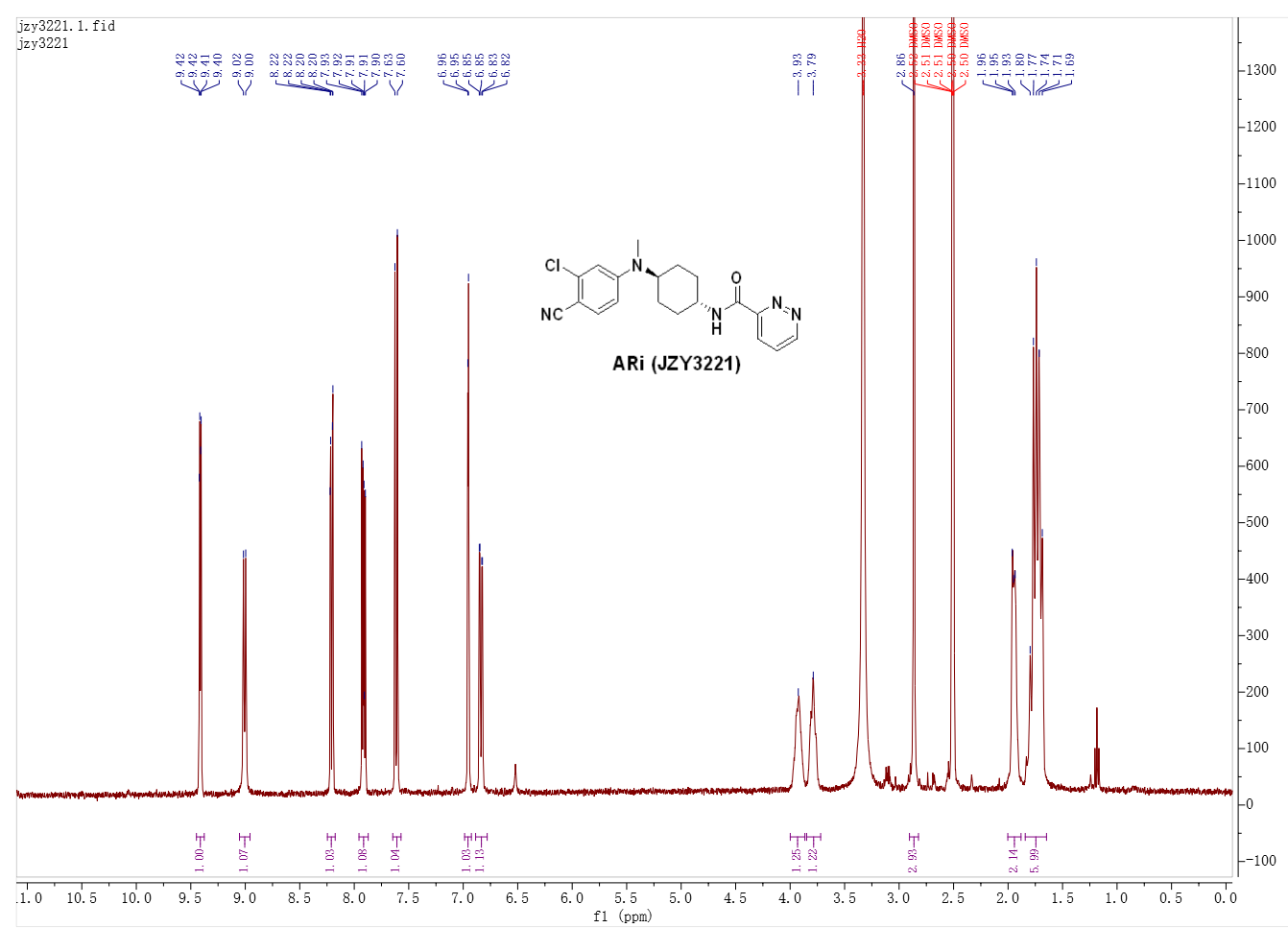


**References**

1. Chen, Z. *et al.* Discovery of CBPD-409 as a highly potent, selective, and orally efficacious CBP/p300 PROTAC degrader for the treatment of advanced prostate cancer. *J Med Chem*, ***67***, 5351-5372 (2024). <https://doi.org/10.1021/acs.jmedchem.3c01789>.

2. Xiang, W. *et al.* Discovery of ARD-2585 as an exceptionally potent and orally active PROTAC degrader of androgen receptor for the treatment of advanced prostate cancer. *J Med Chem*, ***64***, 13487-13509 (2021). https://doi.org/10.1021/acs.jmedchem.1c00900.
